# Activity-dependent development of vocal circuits in the neonatal rodent forebrain

**DOI:** 10.1038/s44319-026-00798-1

**Published:** 2026-05-19

**Authors:** Shih-Yun Chen, Hao-Yu Pang, Pao-Wen Fan, Guan-Ying Wu, Wan-Ting Lin, Fu-Chin Liu, Hsiao-Ying Kuo

**Affiliations:** 1 Institute of Neuroscience, National Yang Ming Chiao Tung University, Taipei, Taiwan; 2 Institute of Anatomy and Cell Biology, National Yang Ming Chiao Tung University, Taipei, Taiwan

**Keywords:** Neuroscience

## Abstract

Vocal communication is fundamental for social interaction across species, yet the neural mechanisms that shape vocal circuit development remain poorly understood despite their relevance to neurodevelopmental disorders. Here, we investigate vocal circuit development in mice using isolation-induced ultrasonic vocalizations (USVs) in neonates. An activity-tagging approach identifies the ventromedial prefrontal cortex (vmPFC) as a cortical region strongly activated during USV emission. We find a predictable temporal correlation between vmPFC activity and USV emission using in vivo fiber photometry. Selective activation and inhibition of vmPFC neurons establishes a causal role of vmPFC in vocalization. Interestingly, chronic activation of vmPFC neurons not only increases *Foxp2*, a gene implicated in childhood speech apraxia, but also Vglut1-labeled synapses in the striatum, suggesting that activity-dependent increases in Foxp2 may promote corticostriatal synaptogenesis. Consistent with this finding, neonatal vmPFC activation partially rescues USV deficits in Foxp2 heterozygous mutant mice. Collectively, our results identify the vmPFC-striatal circuit as a key regulator of neonatal vocalization and suggest that Foxp2 may mediate activity-dependent development of vocal circuits.

## Synopsis

The vmPFC-striatal circuit promotes neonatal vocalization and is associated with increased striatal Foxp2 expression and corticostriatal synaptogenesis. Chronic activation of this pathway during early development shapes circuit maturation and partially rescues vocal deficits in genetic models of impaired vocal communication.

- vmPFC neuronal activity is temporally aligned to USV onset, and neural dynamics can reliably distinguish and predict vocal events.
- Activation of vmPFC neurons is sufficient to increase vocal output.
- Chronic vmPFC activation during development enhances putative corticostriatal synapses and Foxp2 expression, accompanied by increased vocal output and partial rescue of vocal deficits in *Foxp2* haploinsufficient mice.

## Abstract (web summary)

The vmPFC-striatal circuit promotes neonatal vocalization and is associated with increased striatal Foxp2 expression and corticostriatal synaptogenesis. Chronic activation of this pathway during early development shapes circuit maturation and partially rescues vocal deficits in genetic models of impaired vocal communication.

## Graphical abstract

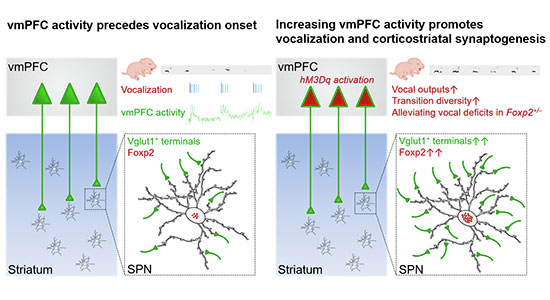

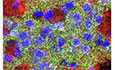

## Introduction

Vocal communication is generated from complex neural circuits that confer vocal diversity, facilitating intraspecific social bonding and emotional expression in mammals. Patients with neurodevelopmental disorders show substantial difficulties in vocal communication in early life, ranging from autism spectrum disorders, intellectual disability, and childhood apraxia of speech (Konopka and Roberts, 2016; Sztainberg and Zoghbi, 2016). Understanding the development of vocal circuits and their molecular underpinnings is essential for developing therapeutic approaches for neurological disorders associated with impaired vocalization.

Classical models of mammalian vocal control propose two partially distinct neural pathways (Jürgens, 2009). The first pathway originates from limbic cortical regions, such as the anterior cingulate cortex (ACC), and relays through the periaqueductal gray (PAG) to brainstem premotor circuits that control vocal motor neurons. The second pathway involves motor cortical projections that can more directly influence brainstem premotor neuron pools and may bypass the PAG. While the classical model emphasizes two partially distinct pathways, subsequent rodent studies suggest that multiple forebrain regions can modulate vocal production through interactions with PAG-dependent circuits. In rodents, studies have revealed additional forebrain structures that modulate vocalization through PAG-dependent gating mechanisms. The brainstem pathway relays vocal signals for vocalization, with the PAG serving as a vocal gate that integrates neural information from the forebrain circuits (Chen et al, 2021; Michael et al, 2020; Tschida et al, 2019; Xiao et al, 2023). The PAG then regulates the activity of the medullary premotor circuits, including reticular formation and the nucleus retroambiguus, and the motoneuron pools to control vocal properties and to coordinate respiration (Jürgens, 2009; Tschida et al, 2019; Concha-Miranda et al, 2022).

Regarding forebrain circuits, recent functional studies in rodents have revealed nuanced modulatory roles for various cortical and subcortical regions during vocalization. The preoptic area and amygdala dynamically regulate the vocal gate PAG, based on contextual and emotional states (Chen et al, 2021; Michael et al, 2020; Xiao et al, 2023). Although the precise contributions to vocalization of cortical regions remain controversial (Hammerschmidt et al, 2015), increasing evidence suggests modulatory functions of the cortex in vocal control (Ivanenko et al, 2020; Sharif et al, 2024). Specifically, the ACC serves as the integrating center of the limbic system and modulates the initiation of social vocalization (Gan-Or and London, 2023). A previous study has illuminated the importance of the medial prefrontal cortex in vocal initiation, underscoring its key role in modulating the vocal center of the brainstem through extensive neural projections (Bennett et al, 2019). In particular, the development of the wiring and refinement of vocal circuits remains elusive.

The molecular mechanisms underlying the establishment of vocal circuits have been examined in cortico-basal ganglia circuits, a key hub that is involved in the integration of sensory and motor information by the brain. The striatum of the basal ganglia receives cortical inputs from the motor, sensory, association, and limbic cortices (Hintiryan et al, 2016; Hunnicutt et al, 2016). Clinical studies have identified mutations of *FOXP2*, a gene enriched in cortico-basal ganglia circuits, are pathologically involved in childhood apraxia of speech (Turner et al, 2013; Vargha-Khadem et al, 1998). Compelling animal studies further revealed that Foxp2-mediated transcriptional regulation is required for vocalization (Fujita et al, 2008; Shu et al, 2005; Teramitsu and White, 2006), especially in developing corticostriatal circuits (Chen et al, 2016; Enard et al, 2009; Kuo et al, 2023; Kuo and Liu, 2017; Sia et al, 2013). *Foxp2* mutations in mice impair non-language vocalizations, including isolation-induced ultrasonic vocalizations (USVs). Specifically, the call rate and duration of isolation-induced USVs are reduced in *Foxp2*^*R552H*^ heterozygote and homozygote (Fujita et al, 2008; Gaub et al, 2010; Groszer et al, 2008; Shu et al, 2005), and *Foxp2* conditional knockout mice (Kuo et al, 2023). Additionally, other USV features, such as the number of elements, frequency jump, and peak sound pressure, are also impaired in *Foxp2* mutant mice (Chen et al, 2016; Gaub et al, 2010; Kuo et al, 2023). However, the mechanistic relationship between Foxp2 and mouse USVs remains debated, as alterations in pup USVs in *Foxp2* mutants may also reflect developmental or motor effects rather than direct changes in vocal circuits (Gaub et al, 2010; Pranic et al, 2022). Consistently, a previous study revealed that region-specific *Foxp2* knockout did not cause vocal impairment in call number or temporal features (Urbanus et al, 2020). Interestingly, Foxp2 has been shown to regulate synaptic remodeling through spinogenesis and neurite outgrowth (Chen et al, 2016; Kuo et al, 2023; Schulz et al, 2010), both are known to be activity-dependent. These observations suggest that neuronal activity may regulate Foxp2 expression, which in turn controls synaptic development and remodeling in vocal circuits.

The dysfunction of corticostriatal circuits has been linked to deficits in speech and vocalization in humans, songbirds, and mutant mice (Enard et al, 2009; Graham and Fisher, 2013; Groszer et al, 2008; Haesler et al, 2004; Murugan et al, 2013; Teramitsu and White, 2006; Vargha-Khadem et al, 1998; Watkins et al, 1999). Despite the important role of corticostriatal circuits in the development of vocal communication, it is yet unknown whether there are specific ensembles of corticostriatal neurons that are engaged in the regulation of vocal communication. In the present study, we used an unbiased activity-tagging strategy to identify cortical regions involved in isolation stress-induced USVs in neonatal mice. We then focused on corticostriatal neurons of the ventromedial prefrontal cortex (vmPFC) that were activated by neonatal USV. Optical imaging with fiber photometry revealed dynamic temporal patterns of neuronal activity in the vmPFC during USV. Furthermore, chemogenetic manipulation with the designer receptor activated by the designer drug (DREADD) demonstrated a causal relationship between vmPFC activity and USV emission. Finally, we showed that activation of the vmPFC not only increased Foxp2 levels but also increased the putative glutamatergic synapses of striatal neurons.

Taken together, our study has identified USV-associated cell ensembles, particularly in the vmPFC-striatal circuit, in the generation of neonatal USV production, and suggests that Foxp2, a gene linked to speech and language, can be regulated by neuronal activity in these ensembles.

## Results

### Trapping vocalization-related corticostriatal ensembles

**Figure 1:**
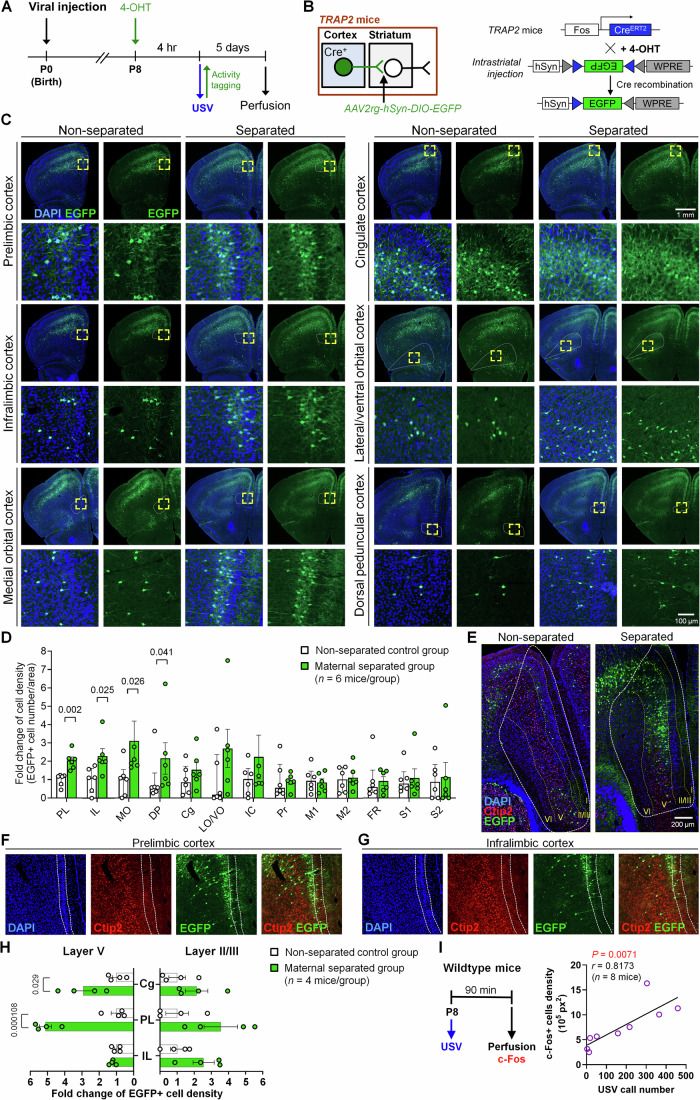
Unbiased labeling of USV-associated ensembles in the ventromedial prefrontal cortex. (**A**) Schematic illustration of the experimental design. (**B**) Viral strategies employed to tag activated neurons projecting to the striatum in the TRAP2 mice during USV emission. (**C**) Neurons activated during 4-OHT exposure were labeled with EGFP. Images were obtained from coronal brain sections spanning multiple anterior-posterior levels, and representative sections containing EGFP-positive neurons in each anatomical region are shown. For each quantified brain region, regions of interest (ROIs) corresponding to anatomically defined cortical subregions are indicated by dashed outlines. Insets (yellow dashed boxes) indicate the locations of higher-magnification images. In a subset of panels, multiple ROIs were derived from the same tissue section; however, these ROIs were carefully defined manually to be spatially non-overlapping. Image reuse within the same section was performed solely to visualize different anatomical regions. For quantitative analyses, ROIs were defined to be mutually exclusive, and no individual cell was counted more than once. Some ROIs shown here are derived from the same sections as those presented in Fig. EV2, but correspond to distinct, non-overlapping regions. (**D**) Quantitative analysis reveals that a higher number of EGFP-positive (+) neurons is found in the prelimbic cortex (PL), infralimbic cortex (IL), medial orbital cortex (MO), and dorsal peduncular cortex (DP) in the maternal separated group, compared with controls across 13 analyzed nuclei. Data were obtained from 12 mice across 4 independent experimental cohorts. (**E**) High-magnification confocal images show EGFP+ cells in the medial prefrontal cortex for both groups. Ctip2-positive cells are predominantly localized in Layer Vb. (**F**, **G**) EGFP-positive cells are observed in both Layer II/III and Layer V of the PL (**F**) and IL (**G**) in the separated group. Dashed lines indicate the boundaries of lamination. (**H**) More EGFP+ corticostriatal neurons are found in Layer V of PL and the cingulate cortex (Cg) in the maternal separation group. Data were obtained from 8 mice across 3 independent experimental cohorts. (**I**) Schematic experimental design and quantification. The number of c-Fos+ cells is positively correlated with the number of USV emissions in wild-type mice. Data were obtained from 8 mice across 4 independent experimental cohorts. Independent *t*-test is used for IL, Pr, M1, M2 in (**D**, **H**); Mann–Whitney *U* test is used for PL, MO, DP, Cg, LO/VO, IC, FR, S1, S2 in (**D**). Pearson correlation is used in (**I**). For the datasets analyzed using parametric analyses, data were presented as mean ± s.e.m. For datasets analyzed using nonparametric analyses, data were presented as median ± interquartile range. Source data are available online for this figure.

**Figure EV1:**
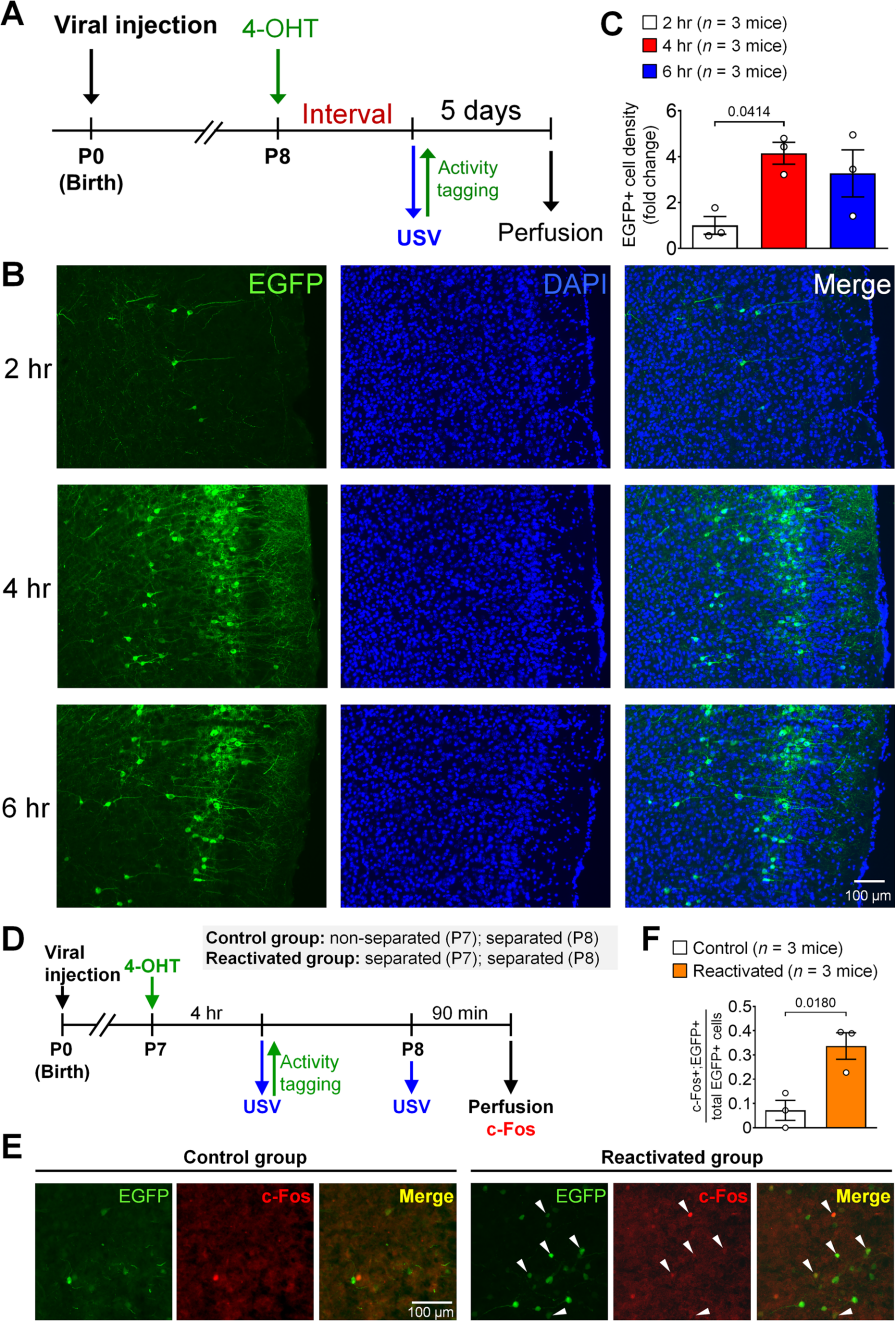
Optimal interval of 4-OHT injection and reactivated neurons in the vmPFC for USV experiment. (**A**) To determine the optimal interval for detecting activated cells during USV emission, we injected 4-OHT into P8 TRAP2 mice whose striatum had been injected with *AAVrg-hSyn-DIO-EGFP* at P0. The mice were separated from the dam 2, 4, or 6 h after 4-OHT injection, and then perfused 5 days later. (**B**) We found fewer EGFP-positive (+) neurons in the prelimbic cortex (PL) in the 2-h interval group compared with the 4-h and 6-h interval groups. (**C**) Quantification of EGFP+ neurons. (**D**) At P7, all mice received an injection of 4-OHT. Four hours after injection, the mice were separated from their mother to trigger isolation stress-induced USV. Littermates that remained with the dam served as non-separated controls. At P8, both groups underwent a 5-min maternal separation and were perfused 90 min later for the detection of c-Fos expression. Cellular reactivation was quantified by measuring the proportion of c-Fos+ cells within the EGFP+ population. (**E**) Reactivated cells (arrowheads) are identified by co-expression of EGFP (green) and c-Fos (red). (**F**) Quantification of reactivated neurons. The proportion of c-Fos+ cells within the EGFP+ population was significantly higher in the reactivated group compared with the control group. One-way ANOVA is used in (**C**). Independent *t*-test is used in (**F**).

To unbiasedly “capture” the corticostriatal neurons activated during vocal communication, we used the genetic activity-dependent tagging strategy to target activated neurons. The targeted recombination of active populations (TRAP2) mice carry the *2A-iCreERT2* allele at the *Fos* locus, in which Cre recombinase is expressed in activated neurons without disrupting endogenous *Fos* expression in a tamoxifen-dependent manner (Allen et al, 2017). Retrograde *AAV2rg-hSyn-DIO-EGFP* viruses were injected into the striatum to label the activated upstream neurons of the striatum during maternal separation (Fig. 1A,B). To determine the optimal temporal window for activity-dependent labeling, 4-hydroxytamoxifen (4-OHT) was administered at different intervals relative to USV exposure (2, 4, or 6 h; Fig. EV1A). Consistent with the short half-life of 4-OHT (6 h in adult mice), EGFP labeling was markedly increased at the 4-h interval compared with 2 h and showed a slight reduction at 6 h (Fig. EV1B,C). Therefore, 4-OHT was administered 4 h before the USV assay in subsequent experiments to tag USV-recruited neuronal ensembles with minimal confounding activity. To assess whether the tagged neurons represent ensembles that can be reactivated during subsequent vocalization, we performed a reactivation assay (Fig. EV1D–F). At P7, mice received 4-OHT and were separated from the dam 4 h later to induce isolation stress-induced USVs and label activated neurons. Littermates that remained with the dam served as non-separated controls. On the following day (P8), both groups were subjected to maternal separation to induce USV emission again, and brains were collected 90 min later for c-Fos immunostaining. We observed EGFP and c-Fos-double-positive neurons in the vmPFC of the reactivated group, indicating reactivation of previously tagged neuronal ensembles. Quantification showed that the proportion of c-Fos-positive (+) cells within the EGFP+ population was significantly higher in the reactivated group compared with controls (Fig. EV1D–F).

**Figure EV2:**
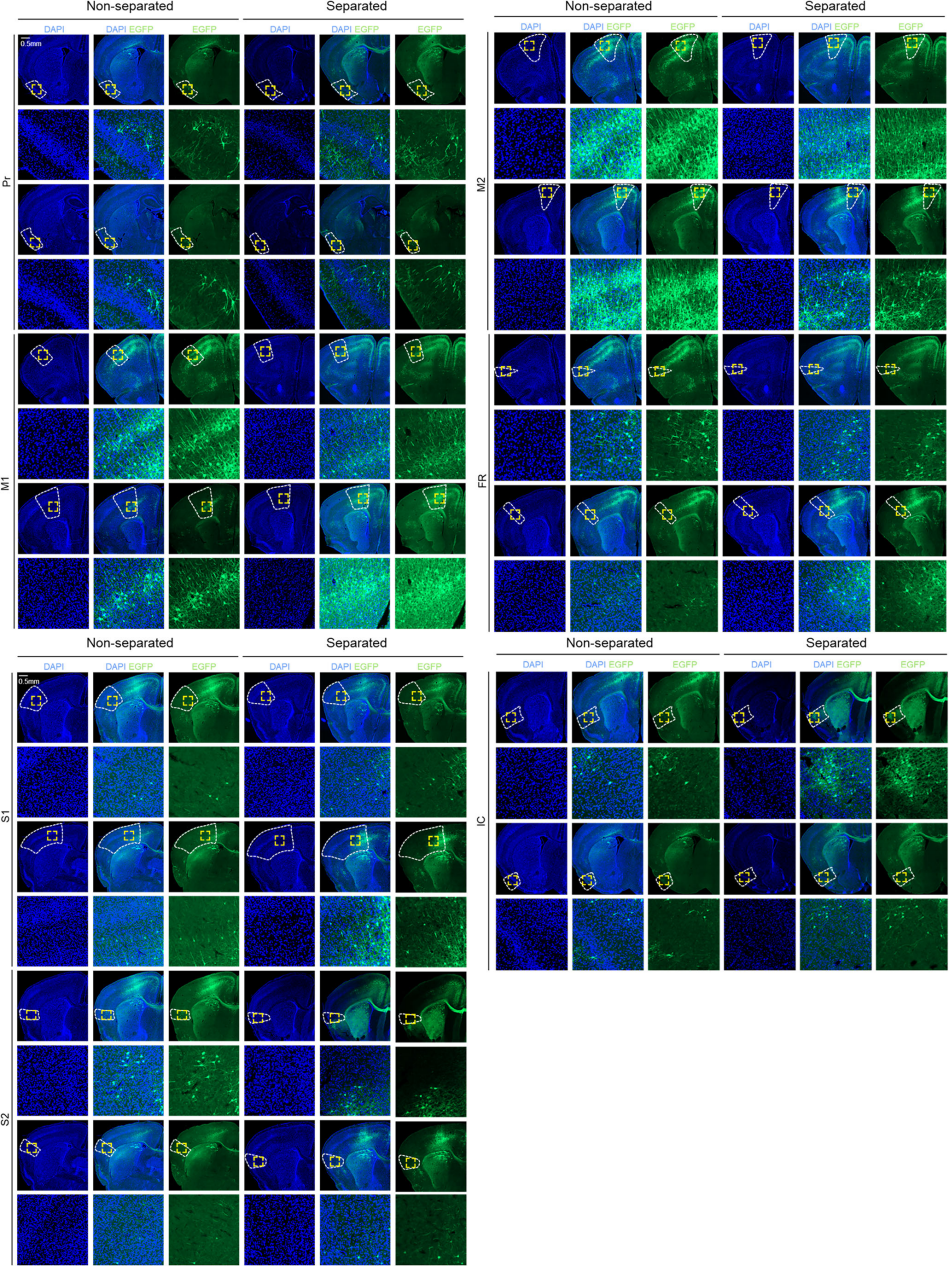
Localization of vocalization-related corticostriatal ensembles in neonatal mice. EGFP signals, identified by GFP immunostaining, indicate the activated neurons during USV emission. EGFP-positive neurons were quantified among 13 cortical regions, including prelimbic, infralimbic, medial orbital, cingulate, lateral/ventral orbital, dorsal peduncular (shown in Fig. 1), piriform (Pr), primary motor (M1), secondary motor (M2), frontal area (FR), primary somatosensory (S1), secondary somatosensory (S2), and insular (IC) cortices. This figure provides additional representative images. In a subset of panels, images are derived from the same sections as those shown in Fig. 1C but correspond to distinct, non-overlapping regions of interest.

Retrograde Cre-dependent viruses, *AAV2rg-hSyn-DIO-EGFP*, were injected into the bilateral striatum of TRAP2 mice at P0 (Fig. 1A,B). At P8, activated corticostriatal ensembles were tagged during isolation-induced USVs 4 h after 4-OHT administration (Fig. 1A,B). In the non-separated control group, TRAP2 pups were not isolated from their dam after 4-OHT administration. We found EGFP+ corticostriatal ensembles in several cortical regions, including the prefrontal cortex, motor cortex, insular cortex, piriform cortex, and somatosensory cortex (Figs. 1C and EV2). Compared to the control group, increased numbers of EGFP+ neurons were detected in subregions of the prefrontal cortex, including the prelimbic cortex (PL), infralimbic cortex (IL), medial orbital cortex (MO), and dorsal peduncular cortex (DP). No significant differences in EGFP+ neurons were found between groups in other cortical regions, including the cingulate cortex and primary motor cortex (Figs. 1C,D and EV2). These results identify the ventromedial prefrontal cortex (vmPFC), including PL, IL, MO, and DP, as potential regions engaged in USV emissions.

To parse out populations of USV activity-trapped neurons in the vmPFC, we performed dual immunostaining for Ctip2 and GFP to reveal the lamination of EGFP+ corticostriatal ensembles in the vmPFC. Ctip2 is a marker of corticofugal neurons, and the expression level of Ctip2 is higher in Layer Vb before P14 (Molyneaux et al, 2007; Ueta et al, 2014). We found that retrograde-labeled EGFP+ corticostriatal neurons of the USV-associated ensemble were located in both Layer II/III and Layer V of PL, IL, and Cg (Fig. 1E), which is consistent with the previous study that corticostriatal projections arise from layers II/III and V of the cerebral cortex (Sohur et al, 2014). Additionally, we observed a higher density of retrograde-labeled EGFP+ cells in Layer V of PL and Cg (Fig. 1F–H), which is in good accord with the prominent corticostriatal projections in Layer V of the cortex (Reiner et al, 2010). These findings allow us to target Layer V neurons in the optical imaging and chemogenetic experiments.

To further examine the relationship between vocal output and vmPFC neuronal activation, we quantified c-Fos expression in the vmPFC following isolation-induced USV emission. The density of c-Fos+ cells in the vmPFC showed a strong positive correlation with the total number of USV calls across animals (Fig. 1I). This relationship provides independent support that vmPFC neuronal activity is tightly associated with USV emission, indicating the involvement of this region in USV production.

### Neuronal activity of vmPFC neurons correlates with isolation-induced vocalization

**Figure 2:**
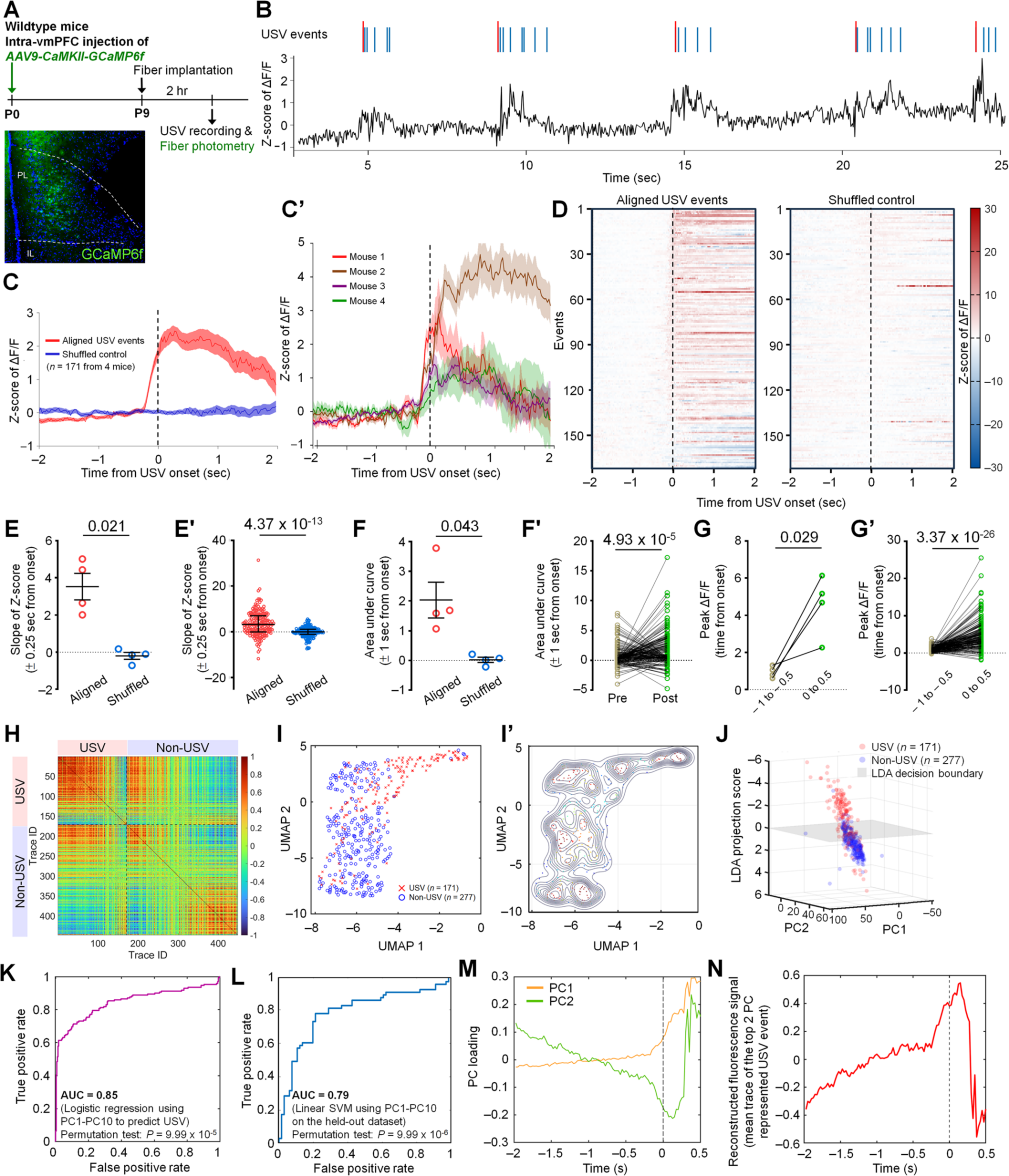
Temporal correlation between calcium transient activity in the ventromedial prefrontal cortex (vmPFC) and ultrasonic vocalization (USV) emission. (**A**) Schematic experimental design (top). The needle tract shows the depth of fiber implantation targeting the prelimbic region (PL), delineated with a dashed line (down). (**B**) Representative 20-s period of fiber photometry signal (Z-scored ΔF/F) with USV events overlaid as raster ticks (blue) and USV onsets with inter-event intervals more than 2 s highlighted in red. (**C**, **C’**) Calcium transient activity recorded during USV emission is aligned to the USV onset. For comparison, the randomly selected time points used as pseudo-events are used as shuffled controls after aligning the time points (blue line in **C**). Data are shown, including 171 events and pseudo-events from 4 mice, and averaged peri-event signals in average (**C**) and individual mice (**C’**). Solid lines represent the mean, and shaded areas indicate ± SEM in each line. (**D**) The normalized signals of each event are aligned and shown in a heat map. The warmer color represents the stronger signals. (**E**, **E’**) The slope of calcium traces within ± 0.25 s of USV onset was significantly higher in the aligned group compared to the shuffled control, as shown in the averages per mouse (**E**; *n* = 4 mice/group) and when calculated across individual events (**E’**; *n* = 171 events/group). (**F**, **F’**) Area under the curve (AUC) analyses reveal higher values in the aligned group compared to the shuffled control within ±1 s of USV onset at the mouse level (**F**; *n* = 4 mice/group). Higher AUC is found within 1 s after USV emission compared to the 1 s preceding USV onset in the aligned group at the event level (**F’**; *n* = 171 events/group). (**G**, **G’**) Peak activity is significantly greater in the aligned group during the 0 to 0.5-s timeframe compared to the ‒1 to ‒0.5-s window. Data are shown as mouse-level averages (**G**; *n* = 4 mice/group) and across individual events (**G’**; *n* = 171 events/group). (**H**) Pearson correlation matrix of all trace patterns (‒1.975 to +0.5 s) was reconstructed from the first 10 principal components (PC). Traces are sorted by linear discriminant analysis (LDA) projection score within each group. Warmer colors indicate a higher pairwise Pearson correlation between traces. (**I**) Uniform manifold approximation and projection (UMAP) using PC1-10, showing clustering patterns of USV and non-USV samples. (**I’**) 2D-contour density plot derived from the UMAP projection shows the density of event numbers. (**J**) Three-dimensional visualization of PCA and LDA projection scores distinguishing USV and non-USV events. The gray horizontal plane shows the LDA decision boundary. (**K**) Receiver operating characteristic (ROC) curve of a logistic regression classifier trained on the first 10 PC. The AUC reflects high discriminative power in predicting USV occurrence from neural trace patterns. (**L**) ROC curve of a linear support vector machine (SVM) trained on PCA-derived PC1-PC10 features, and evaluated on an independently untrained dataset. AUC indicates reliable discriminative performance. (**M**) PC1 and PC2 temporal loadings across time (‒1.975 to +0.5 s of USV onset). PC1 reflects prominent loading changes around USV onset, while PC2 captures a gradual suppression followed by rebound. (**N**) Full reconstruction of the average USV trace pattern using a linear combination of only the top two predictive PCs. The resulting signal captures characteristic dynamics preceding and immediately following USV onset. Data were obtained from 4 mice across 4 independent experimental sessions. Paired *t*-test is used in (**E**–**G**); Wilcoxon signed rank test is used in (**E’**–**G**’); Mann–Whitney *U* test is used in (**J**). For the datasets analyzed using parametric analyses, data were presented as mean ± s.e.m. For datasets analyzed using nonparametric analyses, data were presented as median ± interquartile range. Source data are available online for this figure.

**Figure EV3:**
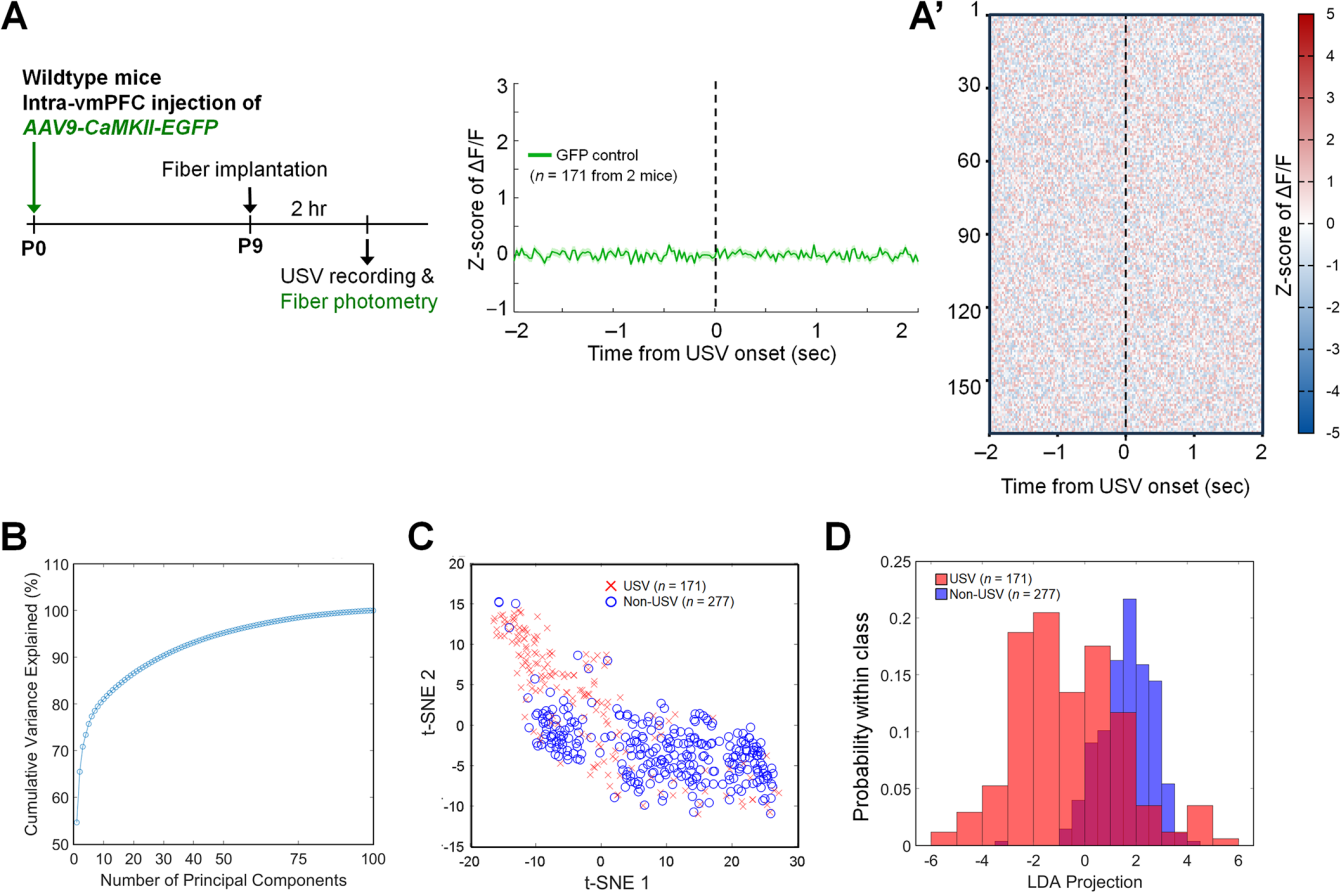
Dimensionality reduction and discriminative projection of vmPFC trace patterns during USV and non-USV periods. (**A**–**A’**) Schematic experimental design and the peri-event GFP signals recorded during fiber photometry. The green line shows the mean value, and the shaded green region indicates the standard error of the mean. The normalized signals of each event are aligned and shown in a heat map. The warmer color represents the stronger signals. (**B**) Cumulative variance explained by the top 100 principal components (PC) derived from USV and non-USV groups. (**C**) The t-SNE projection using PC1-PC10, showing the distribution of trace patterns in an alternative nonlinear projection. (**D**) Histogram of LDA projection scores using PC1-PC10.

To study the activity pattern of vmPFC during USV emission, we performed optical imaging of GCaMP6-based calcium indicators by injecting *AAV9-CaMKII-GCaMP6f* viruses into the vmPFC of P0 wild-type mice. At P9, the calcium transient activity of vmPFC projection neurons was recorded during USVs (Fig. 2A). The vmPFC calcium dynamics were then aligned to USV onset, revealing temporally structured changes in activity surrounding vocalization events (Fig. 2B). To avoid overlap between adjacent vocalization-related signals, we restricted the data analysis to USV events with inter-event intervals longer than 2 s from the preceding one (red-labeled events in Fig. 2B). We observed a sharp increase in calcium transients immediately before USV emission (Fig. 2C,D). To confirm the specificity of population-level responses in response to USV emission, we generated a shuffled control dataset by randomly assigning timestamps and analyzing their corresponding calcium transients. This shuffled control group allowed us to compare the observed calcium transients with those that would otherwise occur by chance (Fig. 2C,D). We found that the slope of calcium traces (±0.25 s from onset) was significantly higher in the aligned USV group compared to the shuffled control group (Fig. 2E–E’). As for the change in neuronal activity before and after USV onset, both the area under the curve (1 s from onset, AUC) of the calcium transient activity and the peak fluorescence activity following USV onset were significantly higher than those preceding USV (Fig. 2F–G’). As a control for potential fluorescence artifacts, fiber photometry recordings were performed in mice injected with *AAV9-CaMKII-EGFP* in the same neuronal population. GFP signals showed no change during USV production, indicating that the observed GCaMP signals reflect calcium-dependent neuronal activity (Fig. EV3A–A’). Collectively, the dynamic pattern of vmPFC neuronal activity shows a strong temporal correlation with USV emission.

### Distinct vmPFC neural dynamics differentiate and predict USV events

To subsequently examine that the calcium transient patterns detected in USV and non-USV periods represented distinct states of neuronal activity, we performed unsupervised principal component analysis (PCA) to analyze the temporal features of calcium transients. We analyzed the peri-event calcium traces from ‒2 to +0.5 s relative to USV onset (*n* = 171, the same dataset in Fig. 2A–F), and another 277 silent periods longer than 2.5 s were considered non-USV group. Cumulative variance explained by each principal component (PC), showing that the first 10 PC elements account for over 85% of the total variance (Fig. EV3B; also see eigenvalue of the top 10 PC and vector of weight of each time point of each PC in Dataset EV1). This result indicates that most of the dominant structures in the original high-dimensional data can be effectively summarized in lower PC, allowing substantial dimensionality reduction without significant loss of information. We next constructed the Pearson correlation matrix of USV and non-USV trace patterns based on the first 10 PC components (Fig. 2H). The USV group (top-left quadrant) shows stronger and more uniform intra-group correlations than the non-USV group (bottom-right quadrant), while inter-group correlations (off-diagonal quadrants) are generally weaker. These results indicate that PCA effectively captures distinct dynamic signatures for distinguishing USV and non-USV events.

To visualize the separability of the activity trace patterns of the USV and non-USV groups, we analyzed the top 10 PC elements for the distribution of USV and non-USV groups in a low-dimensional space by uniform manifold approximation and projection (UMAP) and t-distributed stochastic neighbor embedding (t-SNE) (Figs. 2I,I’ and EV3C). UMAP embedding revealed partial separation between USV and non-USV groups in the nonlinear low-dimensional space. The corresponding 2D contour density plot further highlighted differences in the distribution of activity patterns between the two groups (Fig. 2I,I’). A similar separation was observed in the t-SNE embedding, supporting the distinguishability of trace patterns between the two groups (Fig. EV3C).

To investigate if the top 10 PC we identified are sufficient to distinguish the trace patterns and for further prediction, we next analyzed the trace patterns of the USV and non-USV groups using supervised linear discriminant analysis (LDA). We found that the histogram of LDA projection scores using top 10 PC showed a significant bimodal separation with a large effect size (Cohen’s *d* = ‒1.393), reflecting distinct peri-USV calcium transient dynamics. The supervised LDA classifier achieved 81.47% cross-validated accuracy, indicating reliable classification performance of this model. Permutation test (10,000 shuffles) confirmed that the observed separation in the LDA scores is significantly unlikely to occur by chance (*P* < 0.000001), validating the robustness of the classification (Fig. EV3D). The LDA decision boundary (gray horizontal plane in Fig. 2J) was further projected onto the PC1-PC2 plane, and we found apparent separation between USV and non-USV groups along the LDA axis. This indicates that PCA-transformed temporal dynamics encode sufficient discriminative information for classification.

To evaluate whether the PC of activity trace patterns could accurately predict USV onset, we performed logistic regression using the top 10 PC. The resulting receiver operating characteristic (ROC) curve yielded an AUC of 0.85 (Permutation test: *P* < 0.001), reflecting a high discriminative power in predicting USV occurrence from neural trace dynamics (Fig. 2K). To further assess whether this predictive information generalizes beyond the training dataset, we trained a linear support vector machine (SVM) classifier using PC1-PC10 derived from the same PCA transformation and evaluated its performance on an independent held-out dataset that was not used for model fitting or parameter optimization. The held-out ROC analysis yielded an AUC of 0.79 (Permutation test: *P* < 0.001; Fig. 2L), with an overall accuracy of 70.5%. The classifier exhibited high precision (83.8%) and a low false-positive rate (9.1%), indicating that predicted USV events were reliable, although recall was moderate (49.2%), reflecting a relatively conservative classification at the selected decision boundary. Together, these results indicate that peri-USV neural dynamics contain information predictive of USV events, and that this predictive structure generalizes to independent datasets.

To further better understand the temporal features captured by the PC, we first examined the temporal changes in the PC1 and PC2 loadings, which reflect how each PC contributes across the observation window (Fig. 2M). We then identified the two most predictive PC contributed to the USV group, and reconstructed the fluorescence signal trace pattern around the USV onset based on the mean projections of these two PC (Fig. 2N). The representative trace revealed a steady ramp-up of activity preceding USV onset, followed by a rapid post-event decline afterward, which is closely similar to the average trace pattern of all USV events shown in Fig. 2C.

### Acute activation of vmPFC neurons promotes vocalization

**Figure 3:**
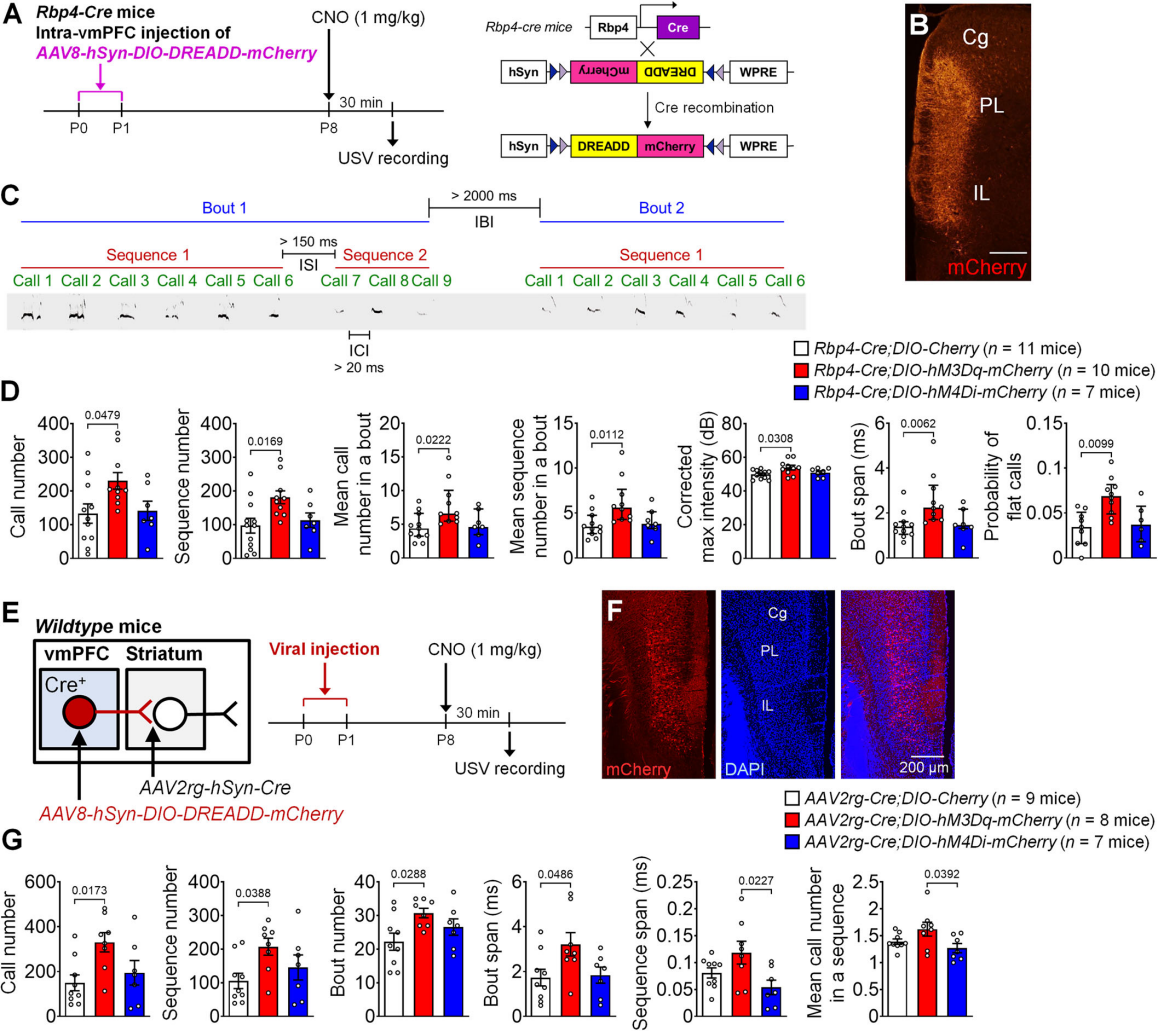
Acute activation of vmPFC neurons increases vocalization. (**A**) Schematic experimental design and anterograde viral infection strategy. (**B**) Representative image showing mCherry expression in vmPFC following *AAV8-hSyn-DIO-mCherry* injection. (**C**) Illustrated definition of USV structure. Vocalizations are segmented into calls and grouped into sequences within each bout. Consecutive calls separated by an inter-call interval (ICI) > 20 ms are defined as distinct calls. Sequences are separated by an inter-sequence interval (ISI) > 150 ms. Bouts are separated by an inter-bout interval (IBI) > 2000 ms. (**D**) Activation of vmPFC neurons increases call and sequence numbers, calls per bout, and sequences per bout during a 5-min recording session. Max amplitude and bout duration are also elevated, and the proportion of flat-type calls is increased. Data were obtained from 28 mice across 7 independent experimental cohorts. (**E**) Intersectional viral strategy to selectively manipulate vmPFC neurons projecting to the striatum. (**F**) Representative images show mCherry expression in vmPFC-striatal projection neurons. (**G**) Activation of vmPFC neurons increases the number of calls, sequences, and bouts, as well as the duration of a bout. The duration of a sequence and the mean call number in a sequence are higher in the inhibition group than in the activation group, with no difference between activation and control mice. Data were obtained from 24 mice across 4 independent experimental cohorts. Cg, cingulate cortex; PL, prelimbic cortex; IL, infralimbic cortex. Kruskal–Wallis one-way ANOVA followed by Dunn’s pairwise multiple comparisons test is used for the mean call number in a bout, mean sequence number in a bout, bout span, and probability of flat call in (**D**). One-way ANOVA with Tukey’s HSD post hoc test is used for other parameters in (**D**) and (**G**). For the datasets analyzed using parametric analyses, data were presented as mean ± s.e.m. For datasets analyzed using nonparametric analyses, data were presented as median ± interquartile range. Source data are available online for this figure.

**Figure EV4:**
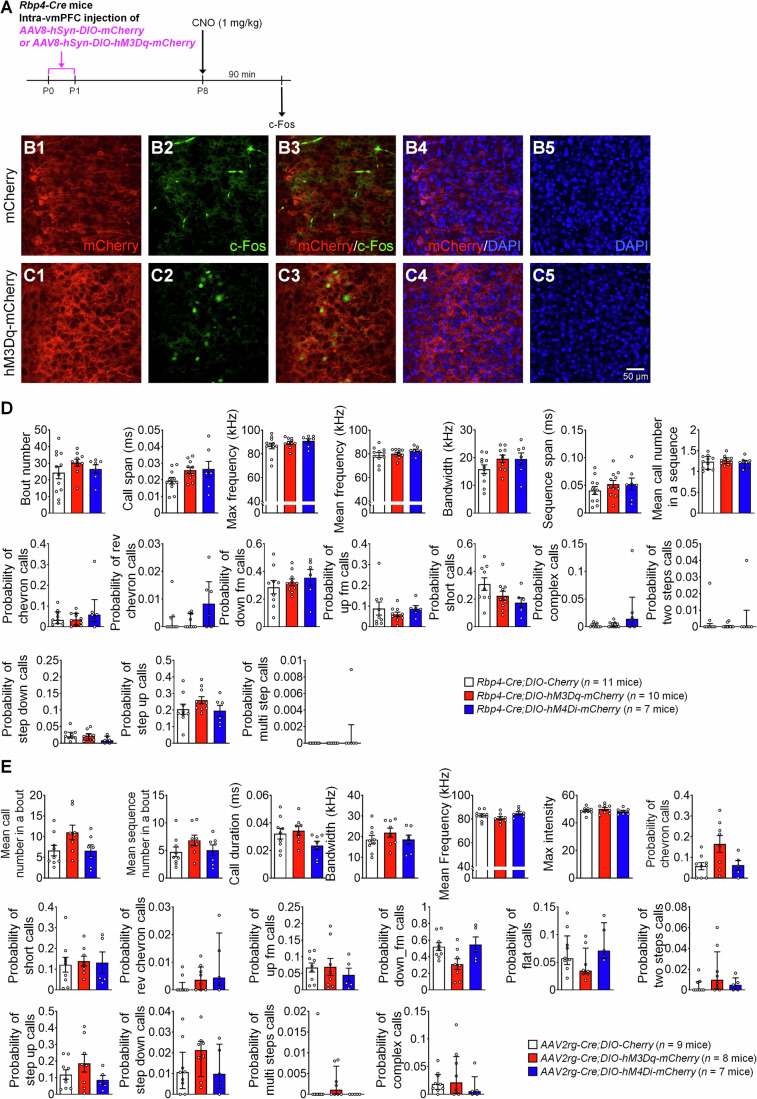
Validation of hM3Dq activation and analysis of USV properties in DREADD-manipulated mice. (**A**) The schematic experimental design. (**B**, **C**) Increased c-Fos expression was observed in vmPFC neurons expressing hM3Dq-mCherry (C1–C5) compared with neurons expressing the control virus (B1–B5) 90 min after CNO administration. (**D**) USV acoustic and syntactic parameters were not significantly altered in mice with acute vmPFC DREADD manipulation. (**E**) USV acoustic and syntactic parameters were not significantly altered in mice with acute manipulation of corticostriatal projection neurons.

Although the temporal pattern of vmPFC neuronal activity was correlated with USV emission (Fig. 2), it is unknown whether vmPFC neurons are causally involved in vocalization. To examine the causal relationship, we microinjected Cre-dependent designer receptors exclusively activated by designer drugs (DREADD) viruses, including *AAV8-hSyn-DIO-mCherry* (control group), *AAV8-hSyn-DIO-hM3Dq-mCherry* (hM3Dq group), and *AAV8-hSyn-DIO-hM4Di-mCherry* (hM4Di group), into the vmPFC of *Rbp4-Cre* mice at P0-P1, and manipulated the activity of DREADD-expressing vmPFC neurons with i.p. injections of designer drug (clozapine N-oxide, CNO) at P8 (Fig. 3A,B). Note that *Rbp4-Cre* driver expresses Cre recombinase in cortical layer V pyramidal neurons (MGI:4367067; KL100) that consist of pyramidal tract (PT) and intertelencephalic corticostriatal neurons (IT) (Gerfen et al, 2013). First, we activated hM3Dq-infected neurons with CNO and harvested the brains 90 min after CNO injection at P8. We then identified activated neurons induced by CNO with c-Fos immunohistochemistry (Fig. EV4A). We found more c-Fos+ cells in the vmPFC of the hM3Dq group (Fig. EV4C1–C5) than that in the control group (Fig. EV4B1–B5). After validation of the efficacy of DREADD manipulation, we i.p. injected CNO to manipulate vmPFC neurons 30 min before USV recording. USV temporal structure was analyzed by segmenting vocalizations into bouts, sequences, and individual calls based on temporal intervals (Fig. 3C). We found that hM3Dq activation of vmPFC neurons acutely increased the total number of calls and sequences compared with control mice. Acute vmPFC activation also increased the number of calls and sequences per bout, maximum intensity, and mean bout length (Figs. 3D and EV4D), suggesting enhanced vocal output and altered sequence organization. To investigate whether acute DREADD manipulation altered USV call-type usage, we quantified the proportion of each call type using VocalMat (Fonseca et al, 2021). The relative proportions of most call types were comparable across groups. However, only the proportion of flat-type calls was significantly increased in the hM3Dq activation group compared with controls, whereas hM4Di inhibition did not produce a significant change (Figs. 3D and EV4D). Additionally, hM4Di-mediated inhibition did not significantly alter these vocal output parameters. Together, these results indicate that vmPFC activation primarily enhances overall vocal output with minimal changes in call-type composition.

Although a high percentage of Cre-expressing neurons were labeled in layer V projection neurons in the *Rbp4-Cre* mice (Gerfen et al, 2013; Munz et al, 2023), only a subset of layer V neurons project to the striatum (Reiner et al, 2010). To directly test whether corticostriatal projection neurons contribute to USV production, we next selectively manipulated vmPFC neurons that project to the striatum using an intersectional retrograde viral strategy. *AAV2rg-hSyn-Cre* was injected into the striatum of wild-type mice, together with Cre-dependent DREADD viruses delivered to the vmPFC to specifically target corticostriatal projection neurons (Fig. 3E). Robust mCherry expression was observed in vmPFC, confirming successful labeling of corticostriatal neurons (Fig. 3F). Activation of these neurons significantly increased the total number of USV calls, sequences, and bouts compared with control animals, whereas inhibition showed no significant effect (Figs. 3G and EV4E). Activation also increased bout span. In addition, the sequence span and the mean number of calls per sequence were significantly higher in the hM3Dq group than in the hM4Di group. These results demonstrate that selective activation of vmPFC corticostriatal projection neurons is sufficient to increase USV production and alter its temporal organization.

### Chronic activation of layer V vmPFC neurons during postnatal development promotes vocalization and alters USV syntax

**Figure 4:**
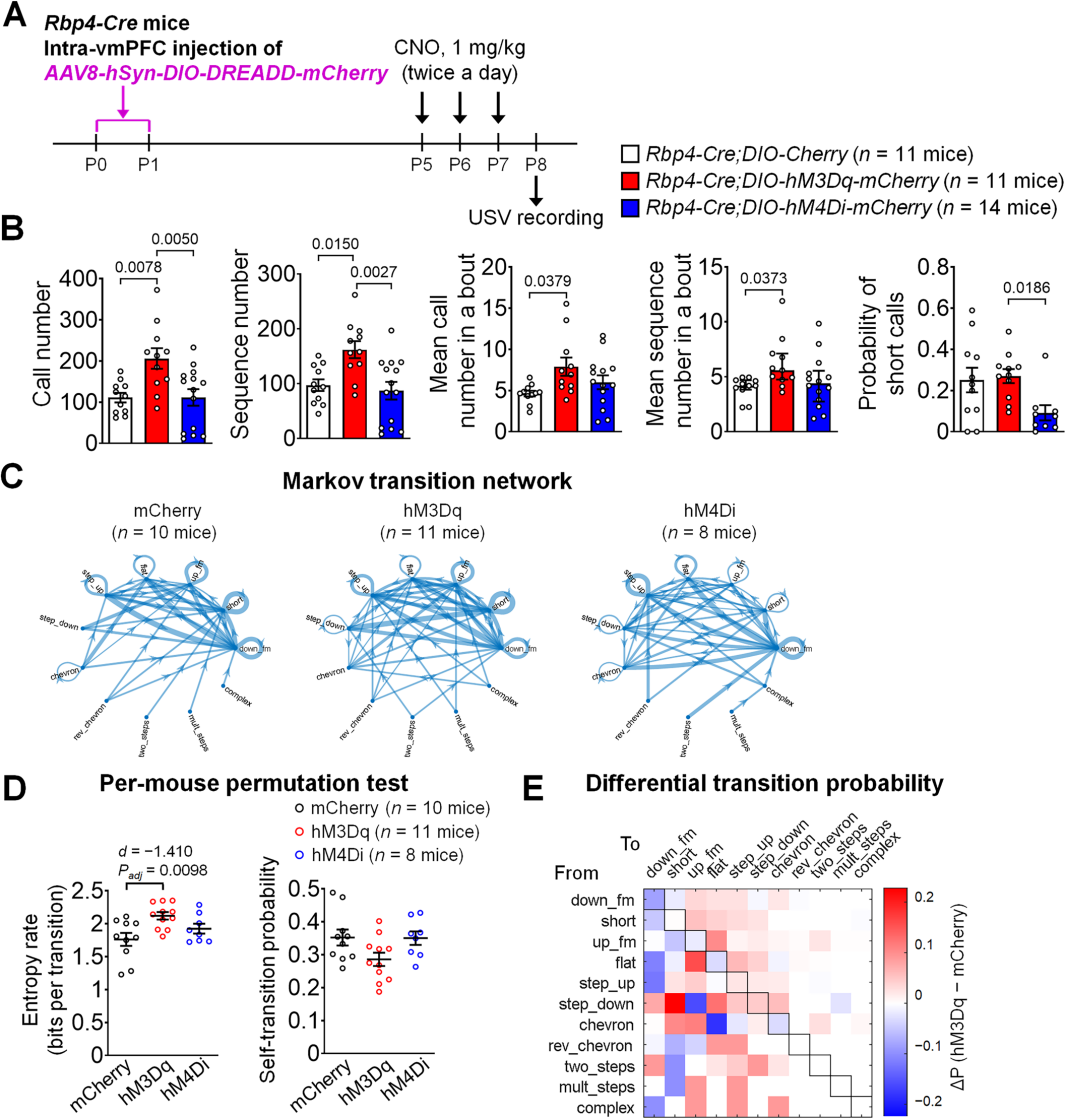
Chronic activation of vmPFC neurons modulates vocalization patterns. (**A**) Schematic experimental design. (**B**) Chronic activation of vmPFC neurons (hM3Dq) increases the total number of calls and sequences compared with mCherry and hM4Di groups. The numbers of calls per bout and sequences per bout are higher in the hM3Dq group than in the mCherry group. Mice with chronic inhibition (hM4Di) emit fewer short-type calls than the hM3Dq group. Data were obtained from 36 mice across 12 independent experimental cohorts. (**C**) Markov transition diagrams of USV syllable sequences in each group. Nodes represent call types, and directed edges represent transition probabilities between successive calls. Edge thickness is proportional to transition probability. Diagrams are based on group-averaged transition matrices. Data were obtained from 29 mice across 11 independent experimental cohorts. (**D**) Per-mouse permutation test of USV transition entropy and self-transition probability across groups. Entropy is higher in the hM3Dq group than in mCherry controls. The self-transition probability does not differ significantly among groups. (**E**) Differential transition probability matrix (ΔP; hM3Dq − mCherry). Rows indicate the preceding call type (From), and columns indicate the subsequent call type (To). Diagonal boxes indicate self-transitions. Warm colors represent increased transition probability in hM3Dq mice, whereas cool colors indicate decreased transitions relative to mCherry controls. One-way ANOVA with Tukey’s HSD post hoc test is used for the call number and sequence number in (**B**). Welch’s one-way ANOVA with Games-Howell post hoc test is used for the mean call number in a bout in (**B**). Kruskal–Wallis one-way ANOVA followed by Dunn’s pairwise multiple comparisons test is used for the mean sequence number in a bout and probability of short calls in (**B**). For the datasets analyzed using parametric analyses, data were presented as mean ± s.e.m. For datasets analyzed using nonparametric analyses, data were presented as median ± interquartile range. Source data are available online for this figure.

**Figure EV5:**
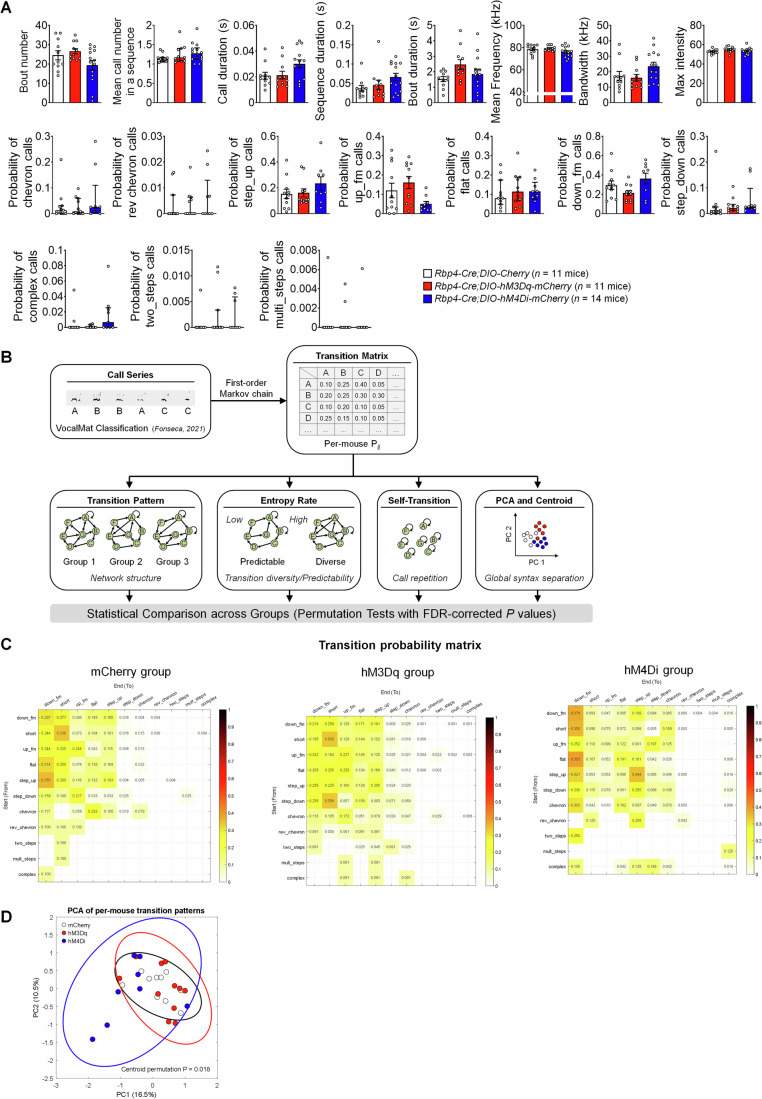
Analysis of USV properties and USV syntax in DREADD-manipulated mice. (**A**) USV acoustic and syntactic parameters were not significantly altered in mice with chronic vmPFC DREADD manipulation. (**B**) Schematic flowchart of Markov chain analysis. (**C**) Transition probability matrix of each group. (**D**) Principal component analysis (PCA) of transition patterns across groups. Group ellipses illustrate clustering in PC space.

To investigate the causal relationship between the wiring of corticostriatal circuits and vocalization in developing mice, we next manipulated vmPFC neuronal activity during postnatal wiring of corticostriatal connectivity to determine whether disrupting circuit activity alters vocalization. After injecting DREADD viruses into the vmPFC of *Rbp4-Cre* mice at P0-P1, CNO was chronically i.p. injected at P5-P7 twice a day to alter the neuronal activity of infected neurons during development. Note that viral construct expression can occur within 3 days following infection of neonatal brains (Cheetham et al, 2015; Kim et al, 2013). Isolation-induced USVs was then subsequently recorded for 5 min at P8 (Fig. 4A). Chronic vmPFC activation significantly increased the total number of calls and sequences, as well as the mean number of calls and sequences within bouts, compared with control mice. In contrast, chronic inhibition of vmPFC neurons reduced USV output relative to the activation group and decreased the probability of short calls (Figs. 4B and EV5A). No significant difference was observed between the hM4Di and control groups. These data suggest that manipulating the activity of neonatal vmPFC neurons during early development alters subsequent isolation-stress-induced USVs in neonatal mice.

To determine whether developmental manipulation of vmPFC activity also alters the syntactic organization of vocalizations across multiple hierarchical levels, we analyzed the sequential structure of USV calls using a first-order Markov chain-based transition analysis (Fig. EV5B). Transition networks constructed from individual call types showed broadly similar connectivity patterns across groups (Figs. 4C and EV5C). Consistent with this observation, per-mouse permutation testing revealed no significant differences in overall transition patterns among groups.

To further quantify global sequence organization, we calculated the entropy rate of the transition matrix, which reflects the diversity of call-to-call transitions. Chronic activation of vmPFC neurons significantly increased the entropy rate compared with controls after false discovery rate (FDR) correction for multiple comparisons (Fig. 4D), representing a transition toward a more diverse and less predictable vocal repertoire. To test whether total call number confounds the entropy rate analysis, we performed a multiple linear regression with total call number included as a covariate. The group effect remained significant after controlling for total call number (*P* = 0.037), indicating that the increase in entropy was not simply due to increased vocal output. We next examined the self-transition ratio of call sequences, which reflects the probability that consecutive calls are of the same type and therefore captures the tendency of sequences to repeat individual call types. In contrast to the entropy analysis, no significant differences in self-transition ratio were detected between groups after FDR correction for multiple comparisons, suggesting that vmPFC manipulation primarily altered the diversity of call-to-call transitions rather than the repetition of individual call types. To visualize how specific call-to-call transitions contributed to these changes, we examined differential transition probabilities (ΔP) between groups. This analysis revealed distributed increases and decreases across multiple call transitions in the activation group relative to controls (Fig. 4E), consistent with the increased transition diversity reflected by the entropy analysis.

Finally, we performed PCA-based comparisons of the global transition probability distribution. PCA of transition matrices revealed significant separation of group centroids in PC space (permutation test, *P* = 0.0183; Fig. EV5D), further supporting that developmental vmPFC manipulation alters the global structure of USV syntax. Together, these results suggest that vmPFC neuronal activity during early development modulates the sequential organization of neonatal vocalizations.

### Chronic activation of layer V vmPFC neurons promotes putative corticostriatal synapses in the neonatal striatum during postnatal development

**Figure 5:**
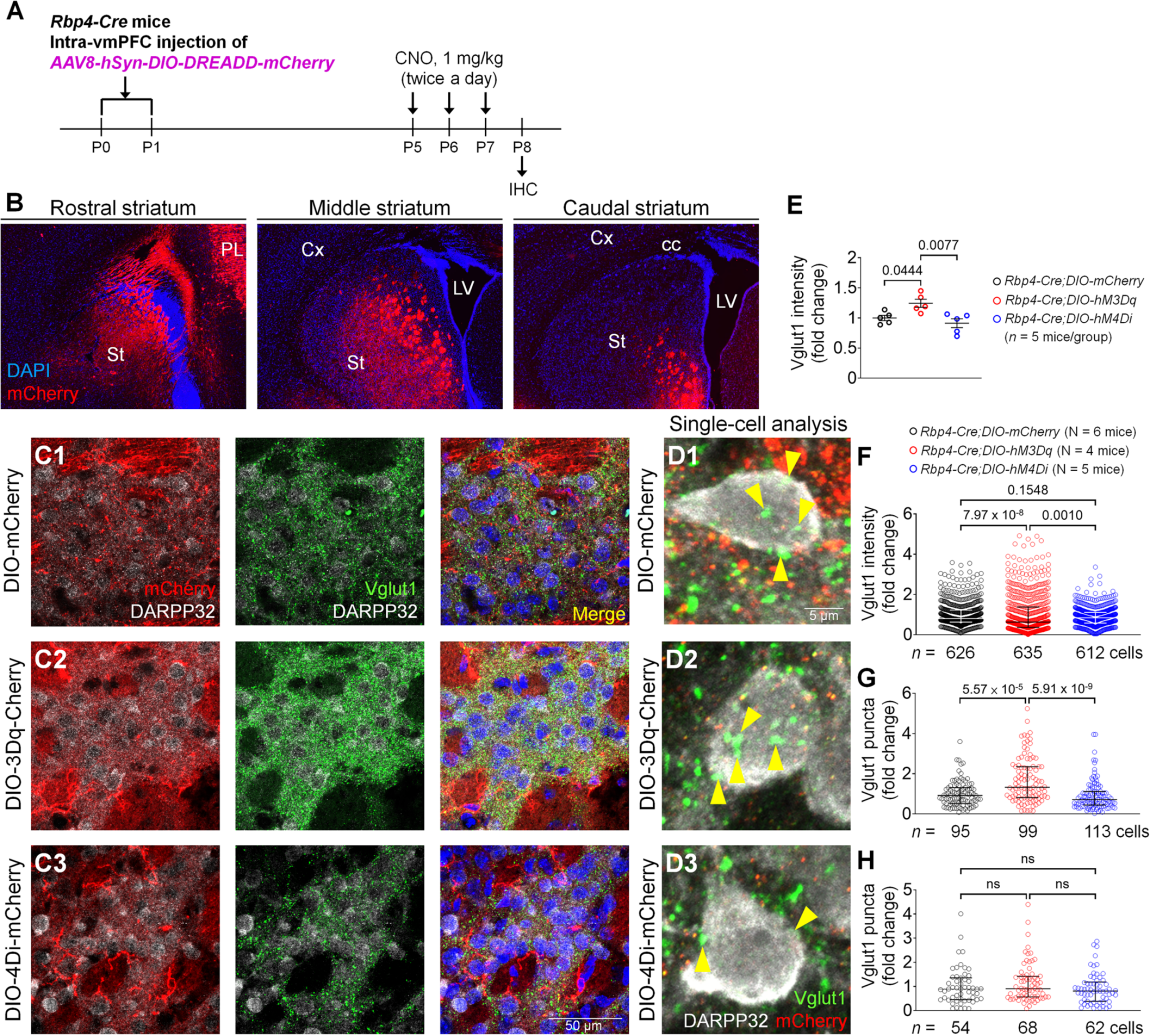
Chronic activation of vmPFC neurons during development enhances putative corticostriatal synapses in the neonatal striatum. (**A**) Schematic experimental design. (**B**) AAV-infected corticostriatal terminals are observed in the medial striatum across rostral to caudal levels. (**C**) The density of Vglut1-positive corticostriatal terminals on the DARPP32-positive spiny projection neuron (SPN) innervated by mCherry-expressing neurons is increased in hM3Dq mice (C2) compared with the control (C1) and hM4Di (C3) groups. (**D**) High-magnification image shows Vglut1-positive puncta (yellow arrowheads) contacting the surface of a single DARPP-32-positive SPN innervated by mCherry-positive axonal terminals. (**E**,** F**) Fold change of the Vglut1 intensity of the DARPP-32-positive SPN innervated by mCherry-positive terminals is increased in the hM3Dq group compared to the mCherry and hM4Di groups at the mouse (**E**) and cellular (**F**) levels. (**G**) The number of Vglut1-positive puncta on the DARPP-32-positive SPN innervated by mCherry-positive terminals is elevated in the hM3Dq group compared to the mCherry and hM4Di groups at the single-cell level. (**H**) No significant difference is observed in Vglut1-positive puncta on the DARPP-32-positive SPN not innervated by mCherry-positive terminals among groups. Data were obtained from 15 mice across 5 independent experimental cohorts. One-way ANOVA with Tukey’s HSD post hoc test for (**E**). Kruskal–Wallis one-way ANOVA followed by Dunn’s pairwise multiple comparisons test is used in (**F**, **G**, **H**). For the datasets analyzed using parametric analyses, data were presented as mean ± s.e.m. For datasets analyzed using nonparametric analyses, data were presented as median ± interquartile range. Source data are available online for this figure.

Previous studies have revealed that neural activity regulates synaptogenesis during the neonatal stage; corticostriatal inputs into the direct and indirect pathways of the striatum, respectively, promote and inhibit excitatory synaptogenesis of spiny projection neurons (SPN) (Kozorovitskiy et al, 2012). Furthermore, elevating cortical activity during early development increases corticostriatal connectivity (Peixoto et al, 2016). Given the significant influence of corticostriatal activity in synaptic wiring of corticostriatal circuits, we postulated that USV-related neuronal activity in the developing corticostriatal pathway may regulate neuronal connectivity. To test this hypothesis, we harvested the brains of pups that received chronic CNO administration after USVs (Figs. 4A and 5A), and immunohistochemically assessed Vglut1 expression in striatal neurons. Note that Vglut1 is mainly located in presynaptic excitatory terminals from the cortex, but not from the thalamus, in the striatum (Kuo and Liu, 2017; Nakamura et al, 2005). The mCherry+ axonal terminals of mPFC-infected corticostriatal neurons were predominantly observed in medial regions from rostral to caudal levels of the striatum (Fig. 5B), which was consistent with the previous report of the topography of corticostriatal axons (Hunnicutt et al, 2016). After chronic hM3Dq activation of vmPFC neurons, we found increased Vglut1 immunoreactivity in DARPP-32+ striatal neurons innervated by mCherry+ terminals (Fig. 5C2,E,F) compared to the control group (Fig. 5C1,E,F) at the mouse and cell levels. To precisely analyze the putative corticostriatal synapses, we also quantified the Vglut1+ puncta in each neuron innervated by mCherry+ or mCherry-negative terminals in SPN neurons, at the single-cell level. The number of Vglut1+ puncta was increased in DARPP-32+ neurons innervated by mCherry+ terminals in the hM3Dq group compared to the control group (Fig. 5G). For chronic hM4Di inhibition, however, no significant alterations were observed between the hM4Di and control groups (Fig. 5C3,D3,F,G). The specificity of chemogenetic manipulations was further demonstrated by the negative results of Vglut1 puncta in mCherry-negative terminals (Fig. 5H). Taken together, these results suggested that chronic activation of vmPFC neurons may facilitate USV emissions by promoting corticostriatal synaptogenesis in the neonatal striatum.

### Chronic activation of vmPFC neurons promotes striatal Foxp2 expression during postnatal development

**Figure 6:**
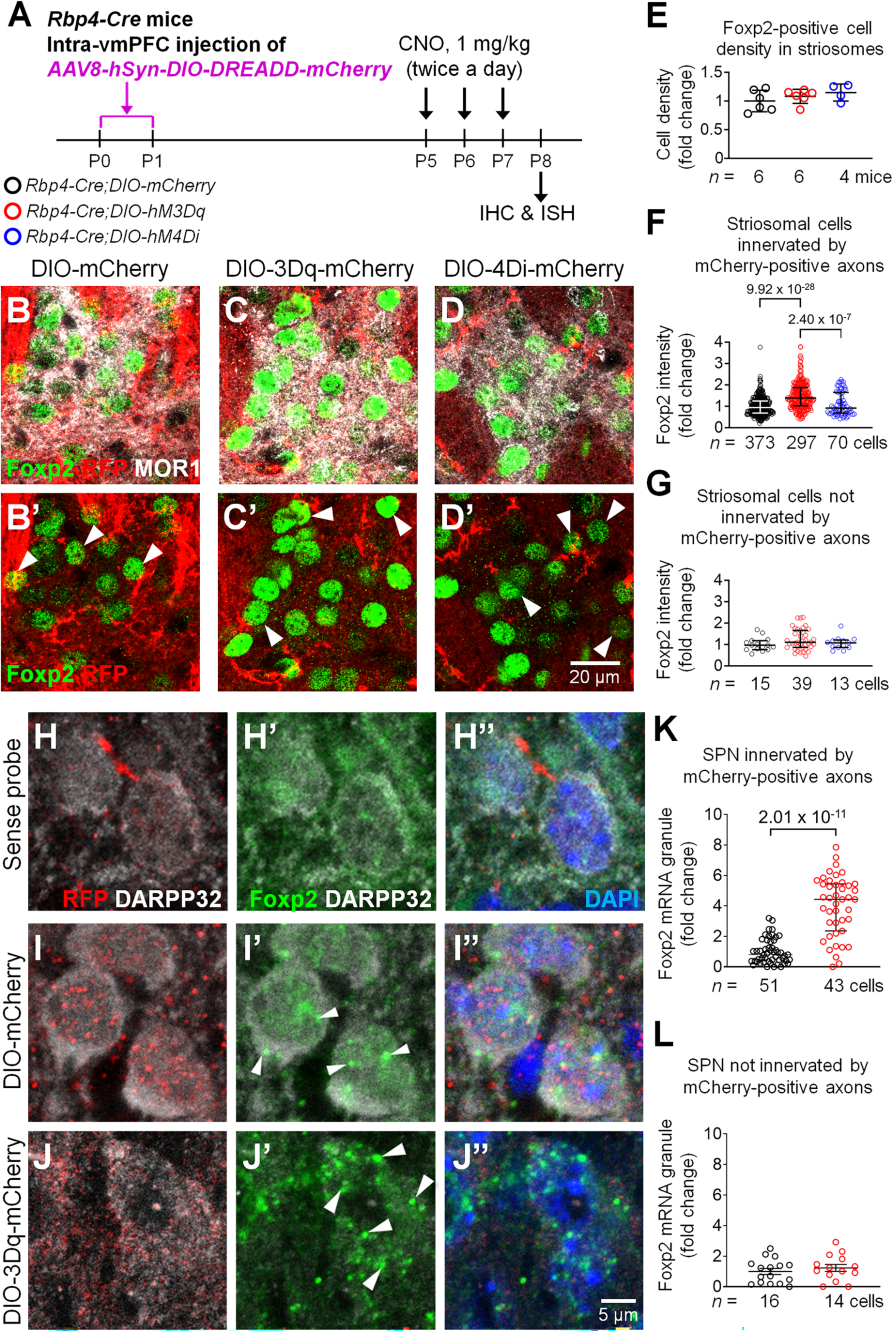
Chronic activation of vmPFC neurons promotes Foxp2 expression in the striatum. (**A**) Schematic experimental design. (**B**–**D’**) Foxp2 immunoreactivity (white arrowheads) is increased after chronic activation of vmPFC neurons in the hM3Dq group (**C**, **C’**) compared with the control group (**B**, **B’**) and the hM4Di group (**D**, **D’**). (**E**) The density of Foxp2-positive cells in striosomes is not changed among groups. (**F**) Quantitative data of (**B**–**D’**) from striosomal neurons innervated by mCherry-positive terminals. Foxp2 immunoreactive intensity in the hM3Dq group is higher than that in the mCherry and hM4Di groups. (**G**) Quantitative data from striosomal neurons not innervated by mCherry-positive terminals. No significant difference among groups. (**H**–**H”**) Striatal sections hybridized with the sense probe. (**I**–**J”**) More *Foxp2* mRNA granules (white arrowheads) are found in the hM3Dq group (**J**–**J”**) compared with the control group (**I**–**I”**). (**K**) Quantitative data of (**H**–**I”**). (**L**) Quantitative data of *Foxp2* mRNA granules from SPN that are not innervated by mCherry-positive terminals. Data were obtained from 16 mice across 6 independent experimental cohorts. One-way ANOVA with Tukey’s HSD post hoc test for (**E**). Kruskal–Wallis one-way ANOVA followed by Dunn’s pairwise multiple comparisons test is used in (**F**,** G**,** K**). For the datasets analyzed using parametric analyses, data were presented as mean ± s.e.m. For datasets analyzed using nonparametric analyses, data were presented as median ± interquartile range. Source data are available online for this figure.

Foxp2 is a key transcription factor in regulating synaptogenesis of developing vocal circuits, including the corticostriatal pathways (Adam et al, 2016; Chen et al, 2016; Enard et al, 2009; Groszer et al, 2008; Kuo et al, 2023). Since chronic activation of vmPFC neurons increased putative glutamatergic numbers as evidenced by enhanced Vglut1 expression, we hypothesized that chemogenetic activation of vmPFC neurons may upregulate Foxp2 levels, which in turn promote synaptogenesis and USV emission. To test this hypothesis, we assessed Foxp2 expression in the striatum of *Rbp4-Cre;AAV8-hSyn-DIO-DREADD-mCherry* mice. Chronic chemogenetic manipulation of vmPFC neurons was performed from P5 to P7 (Fig. 6A). Note that corticostriatal neurons predominantly project to striosomes, a subpopulation of the striatum, in early postnatal stages (Kuo and Liu, 2017; Nakamura et al, 2005; Nisenbaum et al, 1998). In addition, we are particularly interested in striosomal neurons innervated by DREADD+ corticostriatal neurons, as a previous study revealed the role of the striosomal population in vocalization (Kuo and Liu, 2017). We triple immunostained Foxp2, mu-opioid receptor 1 (MOR1, striosomal marker), and RFP (mCherry expressed by infected corticostriatal neurons), and quantified Foxp2 immunoreactivity in the striatum. The results showed that Foxp2 immunofluorescent signals were significantly upregulated in the hM3Dq group in MOR1+ striosomal neurons innervated by mCherry-expressing axonal terminals (Fig. 6B–F). Note that the Foxp2+ cell density was unchanged, suggesting the reduced intensity of Foxp2 may result from activity-dependent regulation of its expression levels, rather than neuronal loss or altered cell fate (Fig. 6E). No significant difference was observed among groups in Foxp2+ striatal neurons without mCherry-expressing axonal terminals (Fig. 6G).

To further investigate *Foxp2* mRNA at the transcription level, we also performed dual in situ hybridization and immunohistochemistry to detect *Foxp2* mRNA expression in DARPP-32+ striatal neurons innervated by mCherry+ axonal terminals. In the section hybridized by the sense riboprobe, no mRNA signal puncta were detected in SPN neurons (Fig. 6H–H”). We quantified the number of *Foxp2* mRNA granules within each SPN. The number of *Foxp2* mRNA granules was increased in the hM3Dq group compared to the control group (Fig. 6H–K), indicating higher *Foxp2* mRNA levels in the hM3Dq group. No significant difference in the number of *Foxp2* mRNA granules was found in SPN without mCherry+ axonal terminals between groups (Fig. 6L). Taken together, these findings suggest an activity-dependent modulation of Foxp2 expression in corticostriatal circuits during postnatal development. Cortical activity may promote the expression level of Foxp2, thereby facilitating synaptic wiring in vocal circuits.

### Chronic activation of vmPFC neurons during postnatal development alleviates abnormal vocalization of mice with *Foxp2* haploinsufficiency, potentially via upregulation of striatal Foxp2

**Figure 7:**
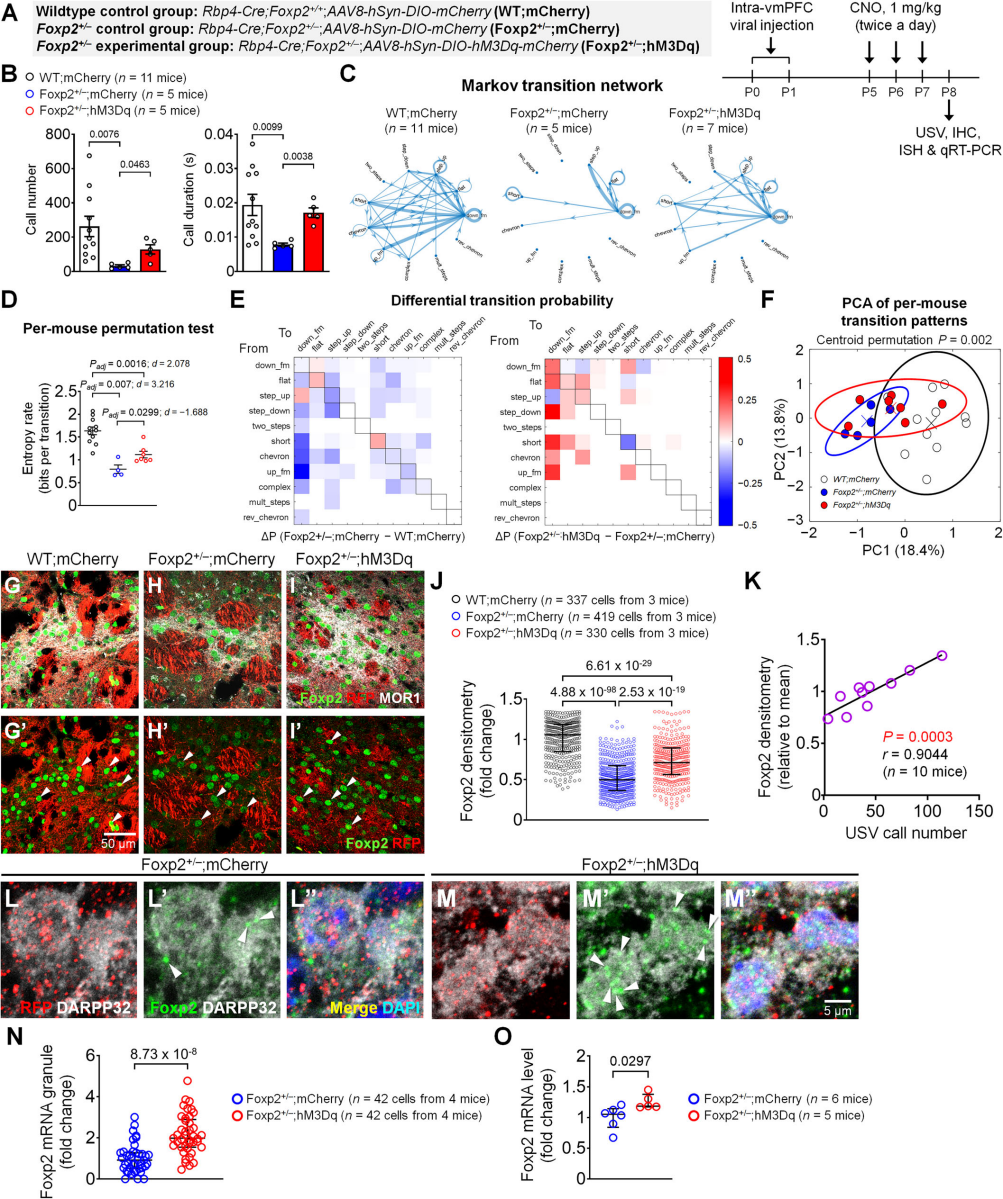
Abnormal USV emission and Foxp2 expression in *Foxp2* heterozygous mice were alleviated by chronic activation of vmPFC neurons during neonatal development. (**A**) Schematic experimental design. To make the figure easier to understand, the abbreviations for each group are shown in the top shaded box. (**B**) Both the number of USV events and duration are improved after increasing the neuronal activities of vmPFC neurons chronically. (**C**) Markov transition networks of USV syllable sequences. Nodes represent call types and directed edges indicate transition probabilities between calls. (**D**) Per-mouse permutation test of transition entropy. The entropy rate is partially rescued in Foxp2^+/−^;hM3Dq group. FDR adjusted *P* values and effect sizes (Cohen’s *d*) are shown. (**E**) Differential transition probability matrices (ΔP). Rows indicate the preceding call type (From), and columns indicate the subsequent call type (To). Warm colors denote increased transition probability and cool colors denote decreased probability relative to the comparison group. Diagonal boxes indicate self-transitions. Certain transitions that are reduced in Foxp2^+/−^;mCherry mice relative to *WT;mCherry* mice show increased probabilities following chronic vmPFC activation. (**F**) Principal component analysis (PCA) of transition patterns across groups. Group ellipses illustrate clustering in PC space. The centroid of each group is labeled with a cross. USV data were obtained from 23 mice across 7 independent experimental cohorts. (**G**–**I’**) Reduced Foxp2 intensity in Foxp2^+/−^;mCherry mice (**H**, **H’**) is partially rescued by chronic activation of vmPFC neurons in Foxp2^+/−^;hM3Dq group (**I**, **I’**), although the Foxp2 level of Foxp2^+/−^;hM3Dq group does not reach that of WT;mCherry group (**G**, **G’**). Arrowheads indicate Foxp2-expressing cells innervated by mCherry-positive vmPFC neurons. (**J**) Quantitative data of (**G**–**I’**) at the single-cell level. (**K**) Correlation between USV call number and Foxp2 intensity at the single-cell level in *Foxp2*^*+/−*^ mice (including Foxp2^+/−^;mCherry and Foxp2^+/−^;hM3Dq groups). Each dot represents one mouse. Pearson correlation analysis reveals a strong positive correlation (*r* = 0.90) between call number and Foxp2 densitometry. Data were obtained from 10 mice across 5 independent experimental cohorts. (**L**–**M”**) *Foxp2* mRNA granules (white arrowheads) are detected by in situ hybridization. Double immunostaining of RFP and DARPP-32 shows mCherry-positive axonal terminals from infected neurons and striatal spiny projection neurons, respectively. (**N**) Quantitative data of (**G**–**H”**) at the single-cell level. Data were obtained from 8 mice across 3 independent experimental cohorts. (**O**) qRT-PCR data show that *Foxp2* mRNA level is significantly higher in Foxp2^+/−^;hM3Dq group compared to Foxp2^+/−^;mCherry mice. Data were obtained from 11 mice across 3 independent experimental cohorts. Welch’s one-way ANOVA with Games-Howell post hoc test is used in (**D**). Kruskal–Wallis one-way ANOVA followed by Dunn’s pairwise multiple comparisons test is used in (**J**). Mann–Whitney *U* test is used in (**N**). Independent *t*-test is used in (**O**). For the datasets analyzed using parametric analyses, data were presented as mean ± s.e.m. For datasets analyzed using nonparametric analyses, data were presented as median ± interquartile range. Source data are available online for this figure.

To test if Foxp2 upregulation in the striatum is critical to vocalization, we manipulated neuronal activity of vmPFC neurons in the vmPFC of *Foxp2* heterozygous mice (*Foxp2*^*+/−*^) with DREADD during development. We designed a set of experiments in *Foxp2*^*+/−*^ mice because previous studies had revealed that *Foxp2*^*+/−*^ mice show abnormal vocalization throughout their life, especially a reduction in USV emissions (Castellucci et al, 2016; Chen et al, 2016; French et al, 2012; Kuo et al, 2023). We microinjected Cre-dependent hM3Dq viruses into the vmPFC of *Rbp4-Cre;Foxp2*^*+/−*^ mice, and activated vmPFC neurons chronically during P5-P7 with CNO administrations (Fig. 7A). First, we found that reduced event and duration of USVs in *Rbp4-Cre;Foxp2*^*+/−*^*;mCherry* mice (Foxp2^+/−^;mCherry group) were partially rescued in *Rbp4-Cre;Foxp2*^*+/−*^*;hM3Dq* mice (Foxp2^+/−^;hM3Dq group) (Fig. 7B), indicating chronic activation of vmPFC neurons could alleviate abnormal vocalization of *Foxp2*^*+/−*^ mice.

**Figure EV6:**
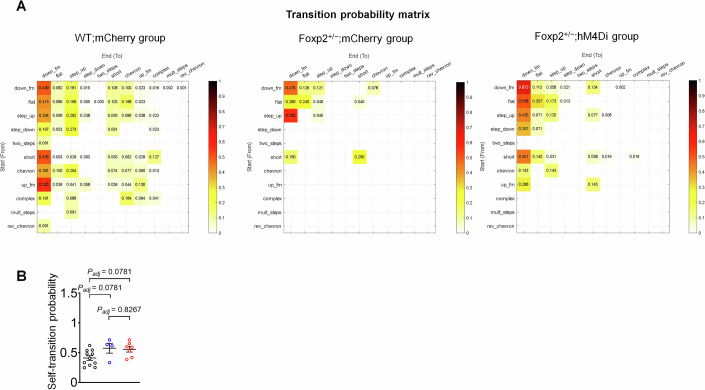
Analysis of USV properties and USV syntax in DREADD-manipulated mice. (**A**) Transition probability matrix of each group. (**B**) Per-mouse permutation test of USV self-transition probability across groups.

Although chronic vmPFC activation partially rescued the reduced call number and duration in *Foxp2*^*+/–*^ mice, we next examined whether chronic vmPFC activation could also ameliorate abnormalities in the sequential organization of USV calls. To address this question, we analyzed USV syntax using a first-order Markov chain framework to examine call-to-call transition structure (Fig. EV5B). Markov transition networks suggested visually distinct transition distributions across groups. In particular, transitions in the Foxp2^+/–^;mCherry group appeared less evenly distributed across call types. This pattern appeared partially restored in the Foxp2^+/–^;hM3Dq group (Fig. 7C). However, per-mouse permutation testing did not detect a significant difference in global transition pattern after FDR correction (WT;mCherry vs Foxp2^+/–^;mCherry: *P*_*adj*_ = 0.054; Fig. 7C; permutation, 50,000 shuffles; Fig. EV6A).

To quantify global sequence organization, we next computed the entropy rate of the transition matrix. Compared with WT mice, *Foxp2* haploinsufficiency markedly reduced the entropy rate. Chronic vmPFC activation significantly increased entropy relative to Foxp2^+/–^;mCherry controls, although entropy remained lower than WT levels (Fig. 7D). To test whether total vocal output confounded entropy-rate differences, we fit a multiple linear regression model including total call number as a covariate. Group effects remained significant *P* value after controlling for call number (Foxp2^+/–^;mCherry vs WT;mCherry: *P* = 0.000206; Foxp2^+/–^;hM3Dq vs WT;mCherry: *P* = 0.0021728), whereas total call number itself was not associated with entropy (*P* = 0.659), indicating that reduced entropy in *Foxp2*^*+/–*^ mice and its partial rescue by vmPFC activation are not driven by differences in call number. Because the self-transition ratio was not significantly different across groups (*P*_*adj*_ > 0.05; Fig. EV6B), the altered entropy rate is unlikely to reflect increased repetition of individual call types. We further examined differential transition probabilities (ΔP) to visualize how *Foxp2* haploinsufficiency and vmPFC activation reshaped specific call-to-call transitions. Relative to WT;mCherry group, *Foxp2*^*+/–*^ mice showed selective increases and decreases in transition probabilities across multiple call types (ΔP: Foxp2^+/–^;mCherry − WT;mCherry; Fig. 7E, left). Comparing Foxp2^+/–^;hM3Dq group to Foxp2^+/–^;mCherry group revealed a complementary pattern of ΔP changes, consistent with partial normalization of transition structure by vmPFC activation (ΔP: Foxp2^+/–^;hM3Dq − Foxp2^+/–^;mCherry; Fig. 7E, right). Finally, PCA of transition matrices showed significant separation of group centroids in PC space (centroid permutation test, 50,000 shuffles; Fig. 7F), supporting that *Foxp2* haploinsufficiency alters the global organization of USV syntax and that chronic vmPFC activation reorganizes this syntax-level structure in *Foxp2*^*+/–*^ mice.

Next, we detected and quantified Foxp2 expression levels in striatal neurons innervated by vmPFC-striatal axonal terminals using immunohistochemistry. We found that Foxp2 immunoreactivity in MOR1^+^ striosomal neurons innervated by RFP/mCherry+ terminals was increased in Foxp2^+/−^;hM3Dq group compared to Foxp2^+/−^;mCherry group (Fig. 7G–J). Additionally, to determine whether the partial rescue of USV production was associated with increased Foxp2 expression, we examined the relationship between Foxp2 protein levels and vocal output. Foxp2 protein intensity was quantified at the single-cell level in Foxp2^+/–^;mCherry and Foxp2^+/–^;hM3Dq mice, and Pearson correlation analysis was performed between Foxp2 expression and the total number of USV calls per mouse. We found that Foxp2 expression levels were positively correlated with USV call number (Fig. 7K).

We further performed dual in situ hybridization and immunohistochemistry to investigate the effect of vmPFC activity on *Foxp2* mRNA at the transcriptional level. We quantified *Foxp2* mRNA granules within DARPP-32 + SPN innervated by mCherry+ axonal terminals (Fig. 7L–M”). A significant increase in *Foxp2* mRNA granules was found in the hM3Dq group (Fig. 7M-M") compared to the control group (Fig. 7L-L",N). Upregulation of *Foxp2* at the transcriptional level was further confirmed by the quantitative real-time PCR, which showed a consistent increase in *Foxp2* mRNA in the Foxp2^+/–^;hM3Dq group compared to the Foxp2^+/–^;mCherry group (Fig. 7O). These findings suggest that activity-dependent regulation of striatal Foxp2 expression by vmPFC-striatal projections is involved in vocalization during postnatal development.

## Discussion

The present report, to the best of our knowledge, is the first study investigating the developmental wiring of vocal communication circuits in rodents. We employed an unbiased genetic activity-tagging strategy to identify corticostriatal ensembles in the vmPFC that regulate USV production in neonatal mice. We established a predictable temporal correlation between vocal emissions and dynamic calcium transient changes in the vmPFC. By manipulating vmPFC neurons with the DREADD system, we found that corticostriatal inputs during early development can impact vocal communication through regulating synaptogenesis and Foxp2 expression in the striatum. The critical role of corticostriatal inputs in regulating vocalization-associated Foxp2 expression was further elucidated in a *Foxp2* haploinsufficient animal model.

Activity-dependent maturation of cortico-basal ganglia circuits has been investigated in later stages during postnatal development. A previous study has shown that during the second postnatal week, activation and inhibition of corticostriatal inputs promote and inhibit excitatory spinogenesis of SPN, respectively. The prolonged inhibitory effects on spinogenesis caused by chronic suppression of corticostriatal inputs during P8-P15 persist into early adulthood, highlighting how early-life disruptions of neural activity cause long-lasting effects on circuit development (Kozorovitskiy et al, 2012). Notably, reducing corticostriatal connectivity by suppressing cortical activity rescues abnormalities of excitatory transmission in *Shank3B* knockout, a mouse model of autism (Peixoto et al, 2016). A follow-up study also identified a critical period later in adolescence for the developmental switch of cortical excitatory inputs to the striatum, particularly for input from the cingulate cortex (Peixoto et al, 2016). In the present study, we have investigated how corticostriatal activity from the vocalization-related vmPFC shapes synaptogenesis in the striatum during the first postnatal week. Chronic activation of vmPFC striatal neurons increased putative corticostriatal synapses, suggesting that this period represents a critical window for circuit development similar to those identified in subsequent postnatal stages. In contrast, chronic inhibition of vmPFC neurons did not significantly reduce either overall USV production or Vglut1-labeled putative synapses. While the mean call number tended to be lower after chronic vmPFC inhibition, the variability across animals was relatively high, and the difference did not reach statistical significance.

Previous studies reported that call number and acoustic features may be influenced by developmental delays or alterations in motor activity rather than direct disruption of vocal circuits (Gaub et al, 2010; Pranic et al, 2021). Additionally, region-specific *Foxp2* deletions do not fully recapitulate the USV deficits observed in global *Foxp2* mutants, suggesting that the relationship between Foxp2 expression and vocal behavior is complex (Urbanus et al, 2020). Our findings provide a complementary perspective on this issue. Rather than relying on developmental genetic models, we manipulated neuronal activity in vmPFC circuits during early postnatal development. Chronic activation of vmPFC neurons increased Foxp2 expression and partially rescued USV production in *Foxp2*^*+/−*^ mice. Moreover, Foxp2 expression levels across animals positively correlated with the degree of USV rescue. In addition to changes in call number, we also observed alterations in vocal sequence structure in *Foxp2*^*+/−*^ mice. Therefore, chronic activation of vmPFC neurons can modulate USV syntax in both wild-type and *Foxp2*^*+/−*^ mice. Notably, such syntax changes were also evident in *Foxp2*^*+/−*^ mice following vmPFC activation, suggesting that cortical activity may influence multiple dimensions of vocal behavior, including both call production and vocal sequence organization. These results raise the possibility that Foxp2 functions not only as a developmental transcription factor but also as a mediator of activity-dependent circuit plasticity, thereby influencing vocal communication.

Corticostriatal inputs enhance Foxp2 expression in the striatum during vocal circuit maturation, though the molecular effectors of activity-dependent Foxp2 regulation remain unknown. The POU domain-containing transcription factor POU3F2 (Brn2) is a candidate regulator of postnatal Foxp2 expression, given its sustained expression from the embryonic through the neonatal stage [Allen Developing Mouse Brain Atlas (RRID:SCR_002990) Pou3f2 - RP_090303_03_E04 – sagittal]. A previous study identified that *POU3F2* may positively regulate FOXP2 by binding to the intronic regulatory element of the *FOXP2* gene locus (Maricic et al, 2013), though functional validation is yet to be warranted. A later study showed that the activity-dependent transcription factor CREB (Moore et al, 1996) is sufficient to activate POU3F2 by binding to the promoter of *POU3F2* in GABAergic neurons (Kao et al, 2023). Further investigation is needed to determine whether corticostriatal regulation of Foxp2 expression is regulated through the CREB-mediated Brn2 signaling pathway.

The rodent USV is a functional readout of social communication and affective status, although less complex than in vocal learners. The calling types within a USV bout are different when mice encounter distinct social courtship contexts (Matsumoto and Okanoya, 2018). Playback of isolation-induced USVs from wild-type pups effectively triggers maternal approach behavior, while USVs emitted from autistic pups or scrambled USV sequences were not effective in eliciting maternal approaches (Takahashi et al, 2016). USV is an innate behavior, as pups emit USVs from birth when the development of corticostriatal connectivity is undergoing (Arriaga and Jarvis, 2013; Inaji et al, 2011). Note that the number of neonatal USVs increases along with the developmental maturation of corticostriatal projection, suggesting positive feedback between corticostriatal inputs and Foxp2 expression that underlies the maturation of vocal circuits.

Previous studies reveal that Foxp2 positively controls spinogenesis and neurite outgrowth of SPN (Chen et al, 2016; Kuo et al, 2023; Schulz et al, 2010), suggesting a role of Foxp2-mediated synaptic structural changes through recurrent feedforward activation through the basal ganglia circuits (Kozorovitskiy et al, 2012). Moreover, the mechanisms by which acute corticostriatal activation increases USV emission may differ from the mechanisms regulated by Foxp2 during chronic manipulation of corticostriatal activity. Rather than mediating acute effects of activity changes, Foxp2 can serve as a molecular effector that converts transient corticostriatal inputs to long-lasting synaptic remodeling during postnatal refinement of vocal circuits. Future time course studies tracking longitudinal alterations from early to late developmental stages may help elucidate how early corticostriatal activity may help refine the development of vocal circuits.

Studies in rodents and non-human primates have identified vocalization-related regions within the medial prefrontal cortex (mPFC) (Arriaga et al, 2012; Bennett et al, 2019; Hage and Nieder, 2013; Roy et al, 2016), although the mPFC is not equivalent to Broca’s area, which controls speech function in humans (Le Merre et al, 2021; Mark et al, 2018; Preuss and Wise, 2022). A previous study using the expression of immediate early genes as activity markers has shown that USVs induce immediate early gene expression in the primary motor cortex, premotor cortex, anterior cingulate cortex, and anterodorsal striatum of male mice, but not in the vmPFC (Arriaga et al, 2012). However, a later study using polysynaptic tracing revealed that the prelimbic area of vmPFC is part of the cortical network connected to the vocal motor organ in Alston’s singing mouse, suggesting a potential role for vmPFC in vocal production (Zheng et al, 2022). Our current study demonstrates that the neural activity patterns in the vmPFC associated with USV and non-USV periods are distinct and can be reliably separated through both unsupervised and supervised analytical approaches. These findings further uncover the potential involvement of vmPFC in vocal initiation. We subsequently demonstrate the causal role of vmPFC in establishing corticostriatal projection by promoting synaptogenesis in the striatum, suggesting an integrating function of vmPFC in affective status, social interaction, and vocal communication. However, future investigations are warranted to determine whether the increased Vglut1+ synapses observed in the striatum originate preferentially from the vmPFC. Another compelling question is how vmPFC-striatal projections integrate with midbrain and brainstem vocal circuits, particularly whether vmPFC-striatal neurons project to the vocal gate in the PAG, given that most of the USV-associated ensembles in the vmPFC consist of Ctip2+ pyramidal tract neurons.

In conclusion, our study uncovers a previously unexplored regulatory framework, indicating a key role for vmPFC-striatal ensembles in regulating vocal circuit development through striatal Foxp2 expression and synaptogenesis. The vmPFC-striatal circuit may represent a potential therapeutic target for the treatment of vocal disorders.

### Limitations

One limitation of the present study is that c-Fos is not a perfect indicator of neuronal activity, as some active neurons may not express Fos (false negatives), and Fos expression can occasionally occur in the absence of strong neuronal firing or be unrelated to the behaviors we studied (false positives). The TRAP2 experiment should therefore be interpreted as identifying candidate neuronal populations engaged during USV production rather than definitively labeling all neurons involved in vocalization.

Another limitation is the absence of simultaneous behavioral tracking during experimental sessions, which prevented direct quantification of movement or general arousal states. Because our analyses focused on vocalization-aligned neural dynamics and USV output, we cannot fully exclude the possibility that some observed effects may partially reflect motor- or arousal-related processes associated with vocal behavior. Notably, following chemogenetic manipulation, we did not observe overt changes in general behavioral responsiveness, posture, or body condition during testing sessions compared with the control group, suggesting that the primary effects were related to vocal output rather than gross alterations in overall activity or physical state.

The specificity of the genetic tools used to manipulate prefrontal projection neurons is another concern. Although *Rbp4-Cre* mice target a subset of layer V pyramidal neurons, these neurons are not exclusively corticostriatal and may also include projections to other subcortical regions. To address this concern, we performed an additional intersectional retrograde viral strategy that restricted DREADD expression to vmPFC neurons projecting to the striatum. This manipulation produced effects on USV production similar to those observed in the acute *Rbp4-Cre* experiments. Nevertheless, we cannot completely exclude the possibility that other vmPFC-fugal pathways may also contribute to the regulation of neonatal vocalizations.

It is also worth noting that *Foxp2* heterozygous mice have been reported to exhibit alterations in motor coordination at later developmental stages (Fujita et al, 2008; Shu et al, 2005). However, motor performance was not systematically assessed in neonatal pups in the present study. Therefore, while our findings support a role for vmPFC activity in modulating USV production, we cannot completely exclude the possibility that improvements in vocal output may partially reflect broader changes in motor function. Future studies incorporating quantitative motor assessments will be required to address this issue directly.

Finally, the present study did not employ temporally precise manipulations, such as optogenetic activation, which is technically challenging in neonatal brains, to test whether acute vmPFC stimulation is sufficient to trigger USVs. Future studies using temporally controlled approaches will be necessary to further delineate the causal dynamics of this circuit.

## Methods

**Table:** Reagents and tools table

Reagent/Resource	Reference or Source	Identifier or Catalog Number
**Experimental models**		
C57BL/6JNarl	National Laboratory Animal Center, Taiwan	National Laboratory Animal Center, Taiwan
TRAP2	The Jackson Laboratory	#030323
*Rbp4-Cre*	MMRRC	037128-UCD
*Foxp2* mutant	PMID: 19490899	Wolfgana Enard and Svante Pääbo
**Antibodies**		
chicken anti-GFP	Abcam	#ab13970
rabbit andi-Ctip2	Abclonal	#A20483
goat anti-Foxp2	Abcam	#ab1307
guinea pig anti-Vglut1	Millipore	#AB5905
rabbit anti-RFP	Abcam	#ab62341
mouse anti-RFP	Rockland	#200-301-379
rabbit anti-MOR1	Proteintech	#27625-1-AP
mouse anti-DARPP32	Santa Cruz	#sc-271111
donkey anti-chicken Alexa488	Jackson ImmunoResearch	#703-545-155
donkey anti-goat Alexa 488	Jackson ImmunoResearch	705-545-003
goat anti-guinea pig Alexa488	Invitrogen	A11073
goat anti-rabbit Alexa555	Invitrogen	#A21428
donkey anti-rabbit Alexa647	Jackson ImmunoResearch	#711-605-152
goat anti-mouse Alexa555	Invitrogen	#A21422
goat anti-mouse Alexa647	Jackson ImmunoResearch	#155-605-003
HRP-conjugated sheep anti-digoxigenin	Roche	AB_514500
**Oligonucleotides and other sequence-based reagents**		
PCR primer for TRAP2	The Jackson Laboratory	#030323
PCR primer for *Rbp4*-*Cre* mice	MMRRC	037128-UCD
PCR primer for *Foxp2* mutant	PMID: 19490899	Wolfgana Enard and Svante Pääbo
Foxp2 ISH probe	Allen brain atlas	Probe RP_071211_03_F06
**Chemicals, Enzymes and other reagents**		
clozapine-N-oxide	Sigma	#C0832
4-hydroxytamoxifen	Sigma	#H6278
**Software**		
Avisoft	Avisoft-RECORDER	Avisoft Bioacoustics
VocalMat	PMID: 33787490	Antonio HO Fonseca and Marcelo O Dietrich Lab
Imaris	Oxford Instruments	Bitplane 8.5
**Other**		
*AAV2rg-hSyn-DIO-EGFP*	Addgene	#50457
*AAV9-CaMKII-GCaMP6f*	Addgene	#100834
*AAV8-hSyn-DIO-mCherry*	Addgene	#50459
*AAV8-hSyn-DIO-hM3Dq-mCherry*	Addgene	#44361
*AAV8-hSyn-DIO-hM4Di-mCherry*	Addgene	#44362
*AAV9-CaMKIIa-EGFP*	Addgene	#50469
*AAV8-CaMKIIa-hM3Dq-mCherry*	Addgene	#50764
*AAV2rg-hSyn-Cre*	Addgene	#105553

### Animals and genotyping

All animals including C57BL/6JNarl (National Laboratory Animal Center, Taiwan), *Fos*^*2A-iCreER*^ mice (*TRAP2*, The Jackson Laboratory, #030323), *Rbp4-Cre* mice (MMRRC, 037128-UCD), and the *Foxp2*^*+/−*^ mice (Enard et al, 2009; Gerfen et al, 2013) were housed in a 12-h light-dark cycle-specific pathogen-free room with food and water available ad libitum in the Animal Center of our institute. All animal procedures followed guidelines approved by the NYCU Institutional Animal Care and Use Committee. Both male and female pups were included in all experiments. All the activity-tagging experiments were performed on littermate mice derived from the offspring of intercrosses between heterozygous *TRAP2*^*+/−*^ or *TRAP2*^*+/−*^ with C57BL/6 JNarl mice. For the fiber photometry experiment, wild-type C57BL/6JNarl were used. For the designer receptors exclusively activated by designer drugs (DREADD) experiments, *Rbp4-Cre* pups were obtained from *Rbp4-Cre* mice intercrossed with C57BL/6J mice. For rescue experiments, *Foxp2*^*+/−*^ pups were derived from *Foxp2*^*+/−*^ mice intercrossed with C57BL/6JNarl mice, and *Rbp4-Cre; Foxp2*^*+/−*^ pups were derived from *Rbp4-Cre* mice intercrossed with *Foxp2*^*+/−*^ mice. The genotypes of transgenic mice were determined by polymerase chain reaction (PCR). The primer sequence and conditions of PCR were used based on information from The Jackson Laboratory, MMRRC, and previous studies (Enard et al, 2009; Gerfen et al, 2013).

### Stereotaxic microinjection

Postnatal (P) day 0-P2 pups were first anesthetized with ice for 2 min, and then were placed onto a custom 3D-printed head holder. Some crushed ice was put around the pup to keep it cold for hypothermia. The following viruses were bilaterally injected into pups’ brains with a microinjector (RWD; R-480): *AAV2rg-hSyn-DIO-EGFP* (50 nL; Addgene #50457)*, AAV9-CaMKII-GCaMP6f* (60 nL; Addgene #100834), *AAV8-hSyn-DIO-mCherry* (30 nL; Addgene #50459), *AAV8-hSyn-DIO-hM3Dq-mCherry* (30 nL; Addgene #44361), *AAV8-hSyn-DIO-hM4Di-mCherry* (30 nL; Addgene #44362)*, AAV9-CaMKIIa-EGFP* (30 nL, Addgene #50469)*, AAV8-CaMKIIa-hM3Dq-mCherry* (30 nL, Addgene #50764), and *AAV2rg-hSyn-Cre* (100 nl, Addgene #105553). The final injection coordinates were as follows (the origin of coordinates was set at bregma): Striatum, AP = −1.3 ~ −1.4 mm, ML = ± 0.8 ~ 0.9 mm, DV = −1.5 ~ −1.7 mm; mPFC, AP = −0.8 mm, ML = ± 0.3 ~ 0.4 mm, DV = −0.8 ~ −1 mm. Viruses were injected at a rate of 100 nL/min, and no craniectomy was needed for P0-P2 mice. After injections, the needle was left for 1 min before slow retraction (0.05 mm/s), and the mice were placed on a heating plate until awakened.

### Drug administration

For tagging activated neurons during vocalization, TRAP2 mice received a single intraperitoneal (i.p.) injection of 4-hydroxytamoxifen (4-OHT, 75 mg/kg in 5% tween 80; Sigma, #H6278) 4-h before the behavioral experiment at P8. For manipulating neuronal activity acutely, DREADD-expressing mice received a single i.p. injection of clozapine-N-oxide (CNO, 1 mg/kg; Sigma, #C0832) 30-min before the behavioral experiment. For manipulating neuronal activity chronically, two doses of CNO (1 mg/kg) were administered daily, approximately 12 h apart, from P5 to P7.

### Preparation of mouse brain tissue

For immunohistochemistry (IHC) and in situ hybridization (ISH), mice were deeply anesthetized by isoflurane for over 5 min, and then transcardially perfused with 0.9% sodium chloride (NaCl) followed by ice-cold 4% paraformaldehyde (PFA) in 0.01 M phosphate-buffered saline (PBS). The perfused brains were post-fixed in 4% PFA at 4 °C overnight and were then cryoprotected with 30% sucrose in 0.01 M PBS for at least 48 h. Brains were then sectioned in the coronal plane with a cryostat at 20 μm (Thermo) and stored at 4 °C and −80 °C, respectively, for IHC and ISH. Specifically for the *TRAP2* experiment, coronal brain sections were cut at 20-μm thickness from the frontal cortex posteriorly to the level at which the septum disappears (approximately corresponding to the emergence of the hippocampal commissure), spanning about 2 to 2.3 mm along the rostro-caudal axis depending on brain size. Sections were collected sequentially into 12 parallel series (inter-section interval of 240 μm within each series). One systematically spaced series was selected for immunostaining to ensure unbiased sampling across the full rostro-caudal extent of the region of interest. As a result, approximately 8–10 sections per brain were quantified for unbiased analysis.

For quantitative real-time-PCR (qRT-PCR), mice were deeply anesthetized by isoflurane for over 5 min, and brains were sectioned into 1 mm coronal slices using acrylic brain slice matrix (Alto). The selected slices containing the region of interest were then frozen with dry ice and used for further analysis.

### Immunohistochemistry

IHC was performed as previously described. Shortly, brain sections were mounted to coated slides before being washed with 0.01 M PBS, then permeabilized with 0.2% Triton X-100 in 0.01 M PBS (PBST) for 15 min. Endogenous peroxidase activity was blocked by incubating 3% H_2_O_2_ and 10% methanol in 0.01 M PBST for 5 min. Sections were then incubated with 3% normal donkey serum in 0.01 M PBS for 1 h to block nonspecific binding. After blocking, sections were incubated with the primary antibody in 0.01 M PBS containing 1% normal serum of the host of the secondary antibody, 0.2% Triton X-100, and 0.1% sodium azide at room temperature overnight. Primary antibodies were used as follows: chicken anti-GFP (1:1000; Abcam, #ab13970), rabbit andi-Ctip2 (1:500; Abclonal, #A20483), goat anti-Foxp2 (1:1000; Abcam, #ab1307), guinea pig anti-Vglut1 (1:1000; Millipore, #AB5905), rabbit anti-RFP (1:100 only used in sections following ISH; Abcam, #ab62341), mouse anti-RFP (1:500; Rockland, #200-301-379), rabbit anti-MOR1 (1:500; Proteintech, #27625-1-AP), mouse anti-DARPP-32 (1:500, Santa Cruz, #sc-271111). On the next day, sections were incubated with fluorescence-conjugated secondary antibodies (1:500) in 0.01 M PBS for 1 h. Secondary antibodies were used as follows: donkey anti-chicken Alexa488 (Jackson ImmunoResearch, #703-545-155), donkey anti-goat Alexa 488 (Jackson ImmunoResearch, #705-545-003), goat anti-guinea pig Alexa488 (Invitrogen, #A11073), goat anti-rabbit Alexa555 (Invitrogen, #A21428), donkey anti-rabbit Alexa647 (Jackson ImmunoResearch, #711-605-152), goat anti-mouse Alexa555 (Invitrogen, #A21422), goat anti-mouse Alexa647 (Jackson ImmunoResearch, #155-605-003). Finally, sections were counterstained with 4’,6-diamidino-2-phenylindole (DAPI, 1:5000) for 10 min.

### Digoxigenin-labeled in situ hybridization

ISH was performed as previously described. Shortly, brain sections were post-fixed in 4% PFA/PBS (30 min, on ice), permeabilized with 0.3% Triton X-100 in 0.01 M PBS (15 min), and treated with 0.2 N HCl in DEPC-treated H_2_O (20 min). Proteinase K digestion (10 μg/ml in 0.01 M PBS) was performed at 37 °C for 2–5 min. Sections were then re-fixed with 4% PFA/0.01 M PBS (5 min) and treated twice with glycine (2 mg/ml in 0.01 M PBS, 15 min each). Prehybridization was carried out in 50% deionized formamide/2X SSC at 65 °C for 90 min. Probes were diluted 1:250 to 1:1000 in hybridization solution (50% formamide, 10% dextran sulfate, 0.3 M NaCl, 0.01 M Tris pH 8.0, 500 μg/ml yeast tRNA, 10 mM DTT, 1 mM EDTA pH 8.0, 1X Denhardt’s solution), denatured at 90 °C for 10 min, and applied to samples. Hybridization proceeded at 65 °C for 16 h. Post-hybridization washes included 5X SSC (5 min), 50% formamide/2X SSC (1 h), and RNase A treatment (20 μg/ml, 37 °C, 30 min). Sections were then washed in 10 mM Tris-HCl pH 8.0/500 mM NaCl (10 min), 2X SSC (once), and 0.2X SSC (twice, 20 min each at 65 °C). All TNT buffers contained 0.1% Tween-20. After 0.1% H_2_O_2_/TNT treatment and blocking, sections were incubated overnight with HRP-conjugated sheep anti-digoxigenin (1:1000; Roche, AB_514500). Signals were detected using FITC-conjugated tyramide (1:1000 in dilution buffer; TSA, PerkinElmer) for 10 min. The antisense probe used to detect *Foxp2* mRNA was derived from the Allen Brain Atlas probe RP_071211_03_F06 (NCBI accession number: NM_053242.3). The probe was generated using the following primers: forward primer, 5’-CATCT GCTCA GCCTT CAGC-3’; reverse primer, 5’-TTGGG GCAAT CTCTG ATGA-3’.

### Quantitative real-time polymerase chain reaction

RNA was extracted from the dissected P12 striatal tissue and was reversely transcribed into cDNA. This cDNA was used as the template for the SYBR^®^ Green-based qRT-PCR reaction. PCR primer sequences for *Foxp2* were: forward 5’-GCCAG GCTGT GAAAG CATAT GTGA-3’ and reverse 5’-CATTT GCACT CGACA TTGGG CAGT-3’. The PCR program on the ABI StepOnePlus™ was: 95 °C for 60 s, 45 cycles at 95 °C for 15 s, 60 °C for 15 s and 72 °C for 45 s. Gene expression fold change was calculated using the 2^−ΔΔC^_T_ (cycle of threshold, ^C^_T_) method: 2^−[3Dq(*Foxp2* C^_T_^− _*Gapdh* C^_T_^) − control(*Foxp2* C^_T_^− *Gapdh* C^_T_^)]^.

### Ultrasonic vocalization recording and analysis

Ultrasonic vocalizations (USV) were recorded from P8 pups. Following a 15–30 min habituation period in a sound-attenuating room, each pup was isolated from its mother and littermates and placed in a glass beaker (6 cm diameter) within a soundproof chamber. A condenser ultrasound microphone (Avisoft-Bioacoustics CM16) was suspended 6 cm above the pup within the beaker. Recordings lasted 5 min using an Avisoft UltraSoundGate 116 and Avisoft-RECORDER software. To further characterize USV features, call classification, and call-to-call transitions, USV data were first analyzed using VocalMat [DOI: 10.7554/eLife.59161; (Fonseca et al,)]. with MATLAB (described below).

### Fiber photometry recording and analysis

Neonatal P9 pups were deeply anesthetized with isoflurane (1.5% at a flow rate of 0.5 liter/min) for at least 10 min. After confirming the absence of withdrawal responses, the pups were placed onto a custom 3D-printed head holder. The skin was incised to expose the Bregma (origin), and the right vmPFC was located using the following coordinates: AP, −1.3 mm; ML, 0.3 mm; DV, 0.5 mm. The skull was carefully punctured with needle tips, and optic fibers (diameter of 200 μm and a numerical aperture of 0.37) were slowly implanted into the right vmPFC at a rate of 0.5 mm/min. During implantation, fluorescent signals were monitored to ensure the fiber tip was positioned among GCaMP6+ neurons. Optic fibers were then secured, and incisions were closed using Super Strength Adhesive (3 M, #6004). Following a 2-h recovery period, calcium transients were recorded during vocalization using the Three-color Multichannel Optical Fiber Photometry System (Thinker Tech Nanjing Bioscience Inc). The 470-nm laser beam with a low-level power (~ 30 μW) at the fiber tips was utilized to prevent bleaching during recording. A transistor-transistor logic signal (A-M SYSTEMS Model 2100) was sent to the photometry system and the Avisoft UltraSoundGate 116H recorder to synchronize the recordings. Fluorescent recording traces were analyzed by a MATLAB-based software provided by the manufacturer (Tinker Tech). Briefly, GCaMP6 fluorescence signals were corrected using isosbestic regression-based motion artifact correction (405 nm). Regression coefficients were derived from extended silent periods (> 10 s without USV emission) to estimate and subtract motion-related fluctuations prior to downstream analyses. Calcium transients surrounding USV initiation were quantified by calculating ΔF/F = (F − F0)/F0, where F represents the fluorescence during USV emission, and F0 is the mean baseline fluorescence signal measured over the 2-s period immediately preceding USV initiation. Only USVs preceded by at least 2 s of silence were included in the analysis. Calcium transients during USV emissions were aligned with the initiation of each USV. For comparison, an equal number of random time points served as pseudo-events were randomly generated to match the number of USV events for each mouse (Fig. 2C–G’).

To further assess whether the calcium transient patterns detected in USV and non-USV periods reflect distinct neuronal activity dynamics, we extracted the z-score-normalized peri-event calcium traces from ‒2 to +0.5 s relative to USV onset (*n* = 171; same dataset in Fig. 2A–G). For comparison, we identified another 277 silent periods longer than 2.5 s considered non-USV events (Fig. 2H–K,M,N). Dimensionality reduction was performed using principal component analysis (PCA) in MATLAB to capture the dominant variance components across all traces. An eigenvalue table summarizing the variance explained by the top components and their corresponding temporal patterns was generated (Dataset EV1). To evaluate the consistency of trace dynamics within and between groups, a Pearson correlation matrix was computed across all traces using MATLAB based on the original PCA-reconstructed data. To further explore the structure of the data in reduced dimensions, we applied both uniform manifold approximation and projection (UMAP) and t-distributed stochastic neighbor embedding (t-SNE) using the top 10 PC. t-SNE was implemented using built-in MATLAB functions, while UMAP projections were generated with the UMAP toolbox (Meehan et al, 2025). For classification analysis, linear discriminant analysis (LDA) was performed using the top 10 PC as input features, and 10-fold cross-validation was used to compute classification accuracy. To quantify the magnitude of separation in the LDA projection space, Cohen’s d was calculated, and group differences were assessed using the Mann–Whitney *U* test, along with a permutation test (10,000 iterations). In parallel, a logistic regression classifier was trained on the top 10 PC features, and receiver operating characteristic (ROC) analysis was conducted to evaluate performance with the functions implemented in MATLAB. The statistical significance of the AUC was assessed using 10,000 label shuffles via permutation testing.

To further assess generalizability, we performed an independent held-out decoding analysis using a linear support vector machine (SVM). PCA was first fit exclusively on the training dataset (same dataset in Fig. 2H–K), and the top 10 PC were retained. The trained PCA transformation was then applied to an independent held-out dataset that was not used during model fitting. A linear SVM classifier was trained on the training dataset, with the regularization parameter optimized using cross-validation. The final trained SVM model was subsequently evaluated on the independent held-out dataset without retraining. Classification performance was quantified by accuracy, precision, recall, false-positive rate (FPR), false-negative rate (FNR), and the area under the ROC curve (AUC; Fig. 2L). The statistical significance of the held-out AUC was assessed using permutation testing with 10,000 random label shuffles. All analyses were performed in MATLAB using built-in functions.

### USV syntax Markov-chain analysis and statistics

Because reliable estimation of Markov transition probabilities requires sufficient sampling of call sequences, mice emitting fewer than 50 USV were excluded prior to syntax analysis in the chronic DREADD manipulation experiment (Fig. 4). In the Foxp2 rescue experiment (Fig. 7), however, a substantial proportion of mice in the *Foxp2* heterozygotes emitted fewer than 50 USV. Applying this threshold would have resulted in the exclusion of most animals. Therefore, no minimum call-count threshold was imposed, and all animals were included in the analysis. USV sequence structure was quantified using a bout-based Markov chain framework. For each mouse, USV calls were assigned to discrete syllable classes (“states”) and organized into bouts. Only within-bout call-to-call transitions were counted, and no transitions were allowed across bout boundaries. For each mouse, a state-by-state transition count matrix (C) was constructed by tallying adjacent call transitions. The corresponding transition probability matrix (P) was computed by row-normalizing C to obtain conditional probabilities.

$${P}_{{ij}}=P({X}_{t+1}=j|{X}_{t}=i)$$

Per-mouse summary metrics were derived from the transition count matrix. The self-transition ratio was defined as the fraction of transitions occurring along the diagonal of C (i.e., same-state transitions) divided by the total number of transitions. Entropy rate was calculated from the transition probability matrix using the empirical stationary distribution and Shannon entropy (in bits), where πi denotes the stationary distribution.

$$H=-\mathop{\sum }\limits_{i}{\pi }_{i}\mathop{\sum }\limits_{j}{P}_{{ij}}log {P}_{{ij}}$$

Group-level transition structure was summarized by averaging per-mouse P matrices (*P*^*(1)*^, *P*^*(2)*^, …) within each experimental group to obtain a group-mean transition probability matrix (*P̄*).

$$\bar{P}=\frac{1}{n}\mathop{\sum }\limits_{k=1}^{n}{P}^{\left(k\right)}$$

Group mean matrices were visualized as heatmaps and converted into directed syntax networks by thresholding edges at *P̄*_*ij*_ ≥ 0.05. For each group, the top transitions were additionally reported by ranking edges in *P̄* (restricted to *P̄*_*ij*_ ≥ 0.05) and exporting the highest-probability edges.

### Permutation tests and multiple-comparison correction

Group differences in transition structure were assessed using seeded, per-mouse permutation tests (50,000 permutations). For global transition-pattern differences, the test statistic was defined as the squared Euclidean distance between group mean transition matrices:

$${T}_{{obs}}=\mathop{\sum }\limits_{i,j}{\left({\bar{P}}_{A,{ij}}-{\bar{P}}_{B,{ij}}\right)}^{2}$$

For scalar metrics (entropy rate and self-transition ratio), the test statistic was defined as the absolute difference in group means.

For the permutation test:

$${T}_{{perm}}=\sum {\left({\bar{P}}_{A,{{\rm{i}}}j}^{p{{\rm{e}}}{rm}}-{\bar{P}}_{B,{ij}}^{{perm}}\right)}^{2}$$

Empirical *P* values were computed using a +1 correction:

$$P=\frac{1+{{\rm{\#}}}\left\{{T}_{{perm}}\ge {T}_{{obs}}\right\}}{{n}_{p{{\rm{e}}}{rm}}+1}$$

For each set of pairwise comparisons, false discovery rate (FDR) correction was applied using the Benjamini–Hochberg procedure.

### Linear model control

To control for potential confounding by call number, a linear model was fit with entropy rate as the dependent variable and total call count plus group as predictors. EntropyRate_i_ = β_0_  + β_1_ (TotalCalls_i_) + β_2_ (Group_i_) + ϵ_i_.

### PCA of transition patterns and centroid permutation test

To compare transition patterns in a reduced feature space, per-mouse transition probability matrices were vectorized and transformed (square-root transform followed by mean-centering to stabilize variance in transition probabilities) prior to PCA. PCA was performed across mice, and eigenvalues, explained variance, and cumulative variance were reported. Group separability in PC space was evaluated by a centroid-based permutation test; group labels were randomly permuted (50,000 permutations), group centroids were recomputed in PC1-PC2 space, and the maximum pairwise centroid distance was used as the test statistic. All analyses were performed using custom scripts in MATLAB.

### Microscopy imaging and data analysis

Images were captured using fluorescence microscopy (Olympus BX63 or BX53) and confocal microscopy (Zeiss LSM 700). Image analysis was performed using ImageJ (http://imagej.nih.gov/ij/) and Imaris software (Bitplane, version 8.5). For imaging-based analyses in TRAP2 experiment, regions of interest extracted from the same section were manually defined based on anatomical landmarks and verified to avoid spatial overlap within the same section prior to quantification. The trapped cell counts were divided by the area of interest to calculate the cell density of EGFP+ neurons with ImageJ. Vglut1 and Foxp2 fluorescence intensities from immunohistochemistry and ISH were measured for individual cells using RGB measure plugins in ImageJ. Vglut1 puncta and *Foxp2* mRNA granules were quantified using the Spots module in Imaris and normalized to the volume of each DARPP-32+ cell, which was identified using the Surface module in Imaris. Spot determination criteria were consistent across all experimental groups to ensure analytical accuracy.

### Experimental design and bias control

Animals were assigned to different groups without formal randomization. In some experiments, group allocation was determined by genotype, whereas in others, animals were assigned to different viral treatments. Animals with comparable body sizes were selected to reduce variability. Blinding was applied in selected experiments. USV recordings were performed without knowledge of group allocation, and confocal imaging was conducted by an experimenter blinded to group allocation. Animals that were noticeably smaller than their littermates at P0 were excluded prior to experiments. In addition, animals in which bilateral viral injections failed to accurately target the vmPFC were excluded from analysis. These exclusion criteria were pre-established prior to data collection.

### Statistical analysis

All data except Figs. 2H–N, 4C–E, and 7C–F in this study were tested for normality using the Shapiro–Wilk test before proceeding with further statistical comparisons. All statistical tests were analyzed in two-sided. For the normally distributed datasets, an independent *t*-test or one-way ANOVA followed by Tukey’s HSD post hoc test was used, and data were presented as mean ± s.e.m. If the homogeneity of variance assumption was violated in normally distributed datasets, independent *t*-test with Welch’s correction or Welch’s one-way ANOVA followed by Games-Howell post hoc test was used. For the datasets without normal distributions, the Mann–Whitney *U* test or Kruskal–Wallis one-way ANOVA followed by Dunn’s pairwise multiple comparisons test were employed for analysis, and data were presented as median ± interquartile range. For the within-group comparisons in Fig. 2E–G’, paired *t*-test and Wilcoxon signed rank test were used, respectively, in datasets with and without normal distribution. All experiments were performed with biological replicates, and no technical replicates were included. The statistical methods used to compare means, along with their detailed descriptions, are summarized in Table EV1. The statistical methods used for each analysis are described in the corresponding figure legends. Statistical analysis was performed with SPSS (IBM, version 21). As for the data in Figs. 2H–N, 4C–E, and 7C–F, statistical analysis was performed with MATLAB built-in functions.

## Supplementary information

Table EV1

Peer Review File

Dataset EV1

Source data Fig. 1

Source data Fig. 2

Source data Fig. 3

Source data Fig. 4

Source data Fig. 5

Source data Fig. 6

Source data Fig. 7

Expanded View Figures

## Data Availability

The raw data for Fig. 1 have been deposited in BioStudies under 10.6019/S-BSST2916. The source data of this paper are collected in the following database record: biostudies:S-SCDT-10_1038-S44319-026-00798-1.
