# Supplementary figures and images for: Activity-dependent development of vocal circuits in the neonatal rodent forebrain
Source: EMBO Rep. 2026 May 19;27(13):3632–64. doi: 10.1038/s44319-026-00798-1 (PMC13354774; doi:10.1038/s44319-026-00798-1)

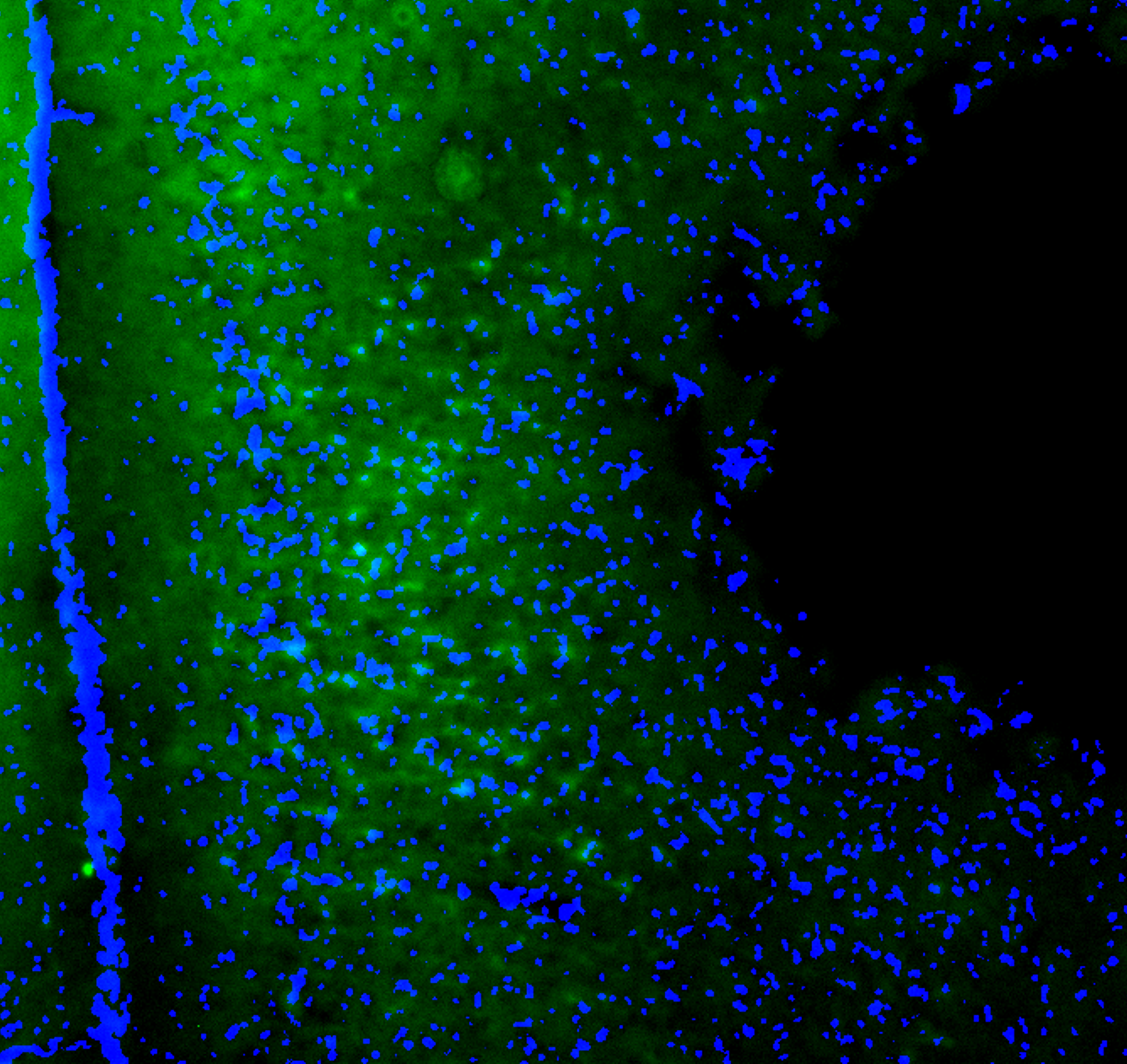

Supplement: Supplementary file 5 — Source data Fig. 2 [file 44319_2026_798_MOESM5_ESM.zip › Figure 2 (V)/2A/2A.tif]

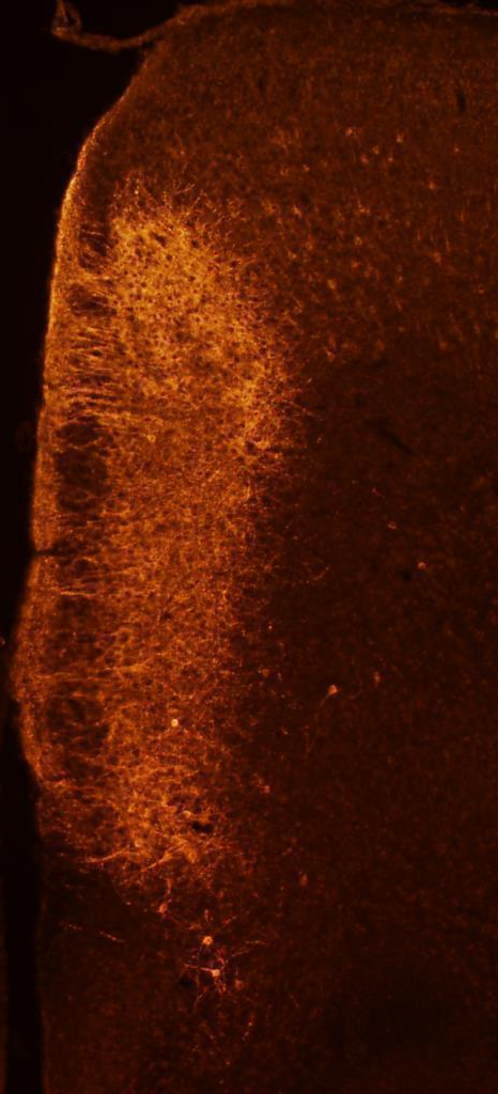

Supplement: Supplementary file 6 — Source data Fig. 3 [file 44319_2026_798_MOESM6_ESM.zip › Figure 3 (V)/3B/3B.tif]

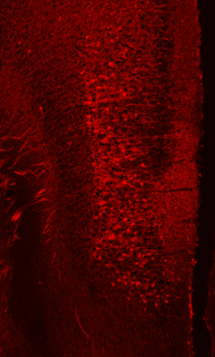

Supplement: Supplementary file 6 — Source data Fig. 3 [file 44319_2026_798_MOESM6_ESM.zip › Figure 3 (V)/3F/3F.tif]

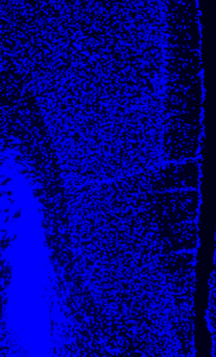

Supplement: Supplementary file 6 — Source data Fig. 3 [file 44319_2026_798_MOESM6_ESM.zip › Figure 3 (V)/3F/3F2.tif]

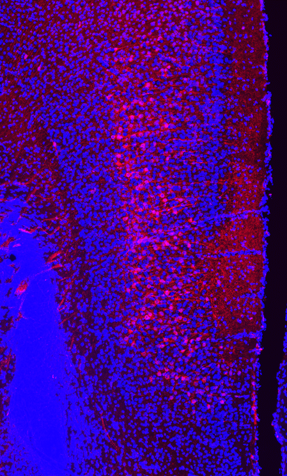

Supplement: Supplementary file 6 — Source data Fig. 3 [file 44319_2026_798_MOESM6_ESM.zip › Figure 3 (V)/3F/3F3.tif]

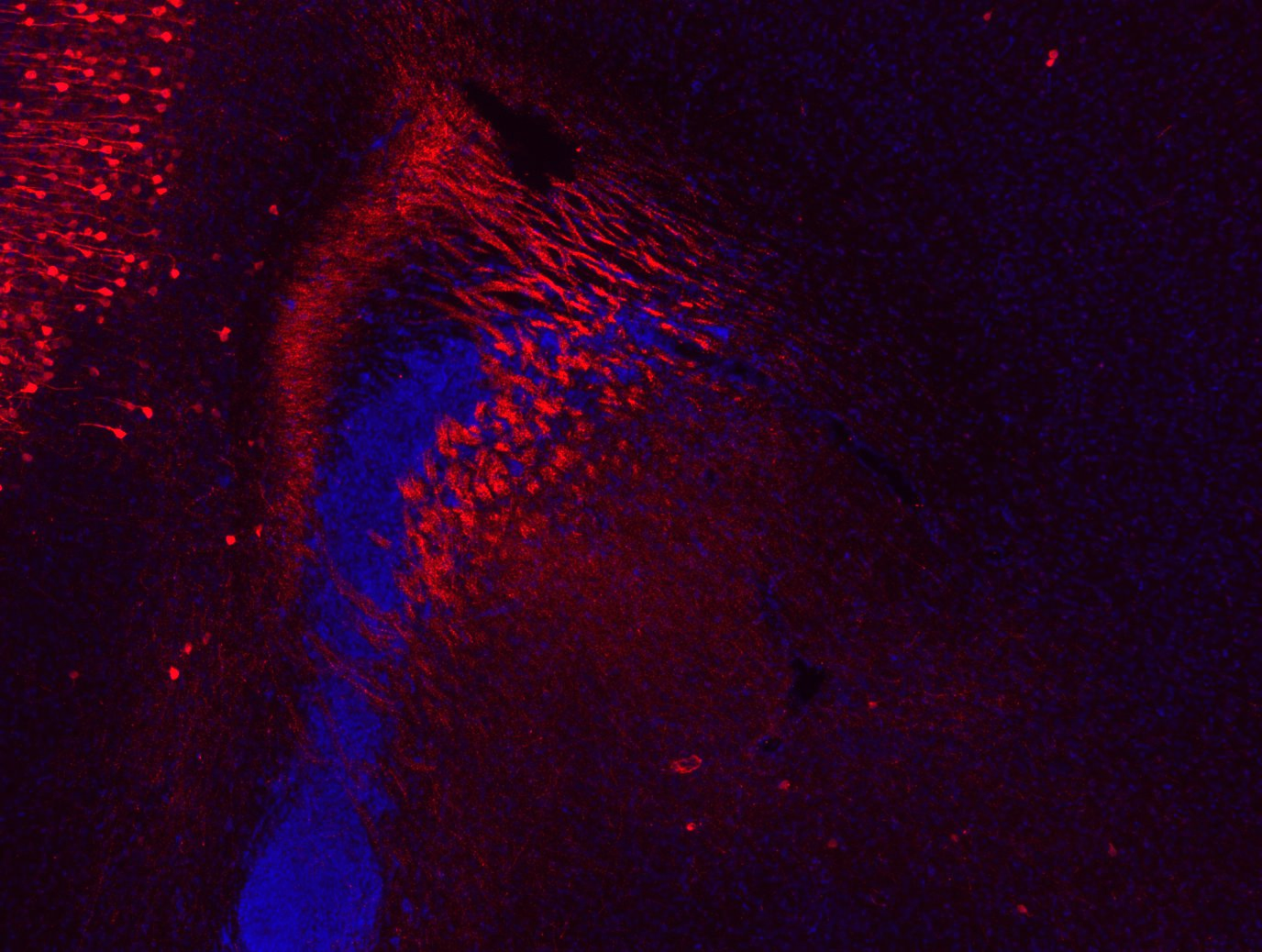

Supplement: Supplementary file 8 — Source data Fig. 5 [file 44319_2026_798_MOESM8_ESM.zip › Figure 5 (V)/5B/DAPI+ RFP rostral striatum( Rbp4-cre 0823-6 ctrl).tif]

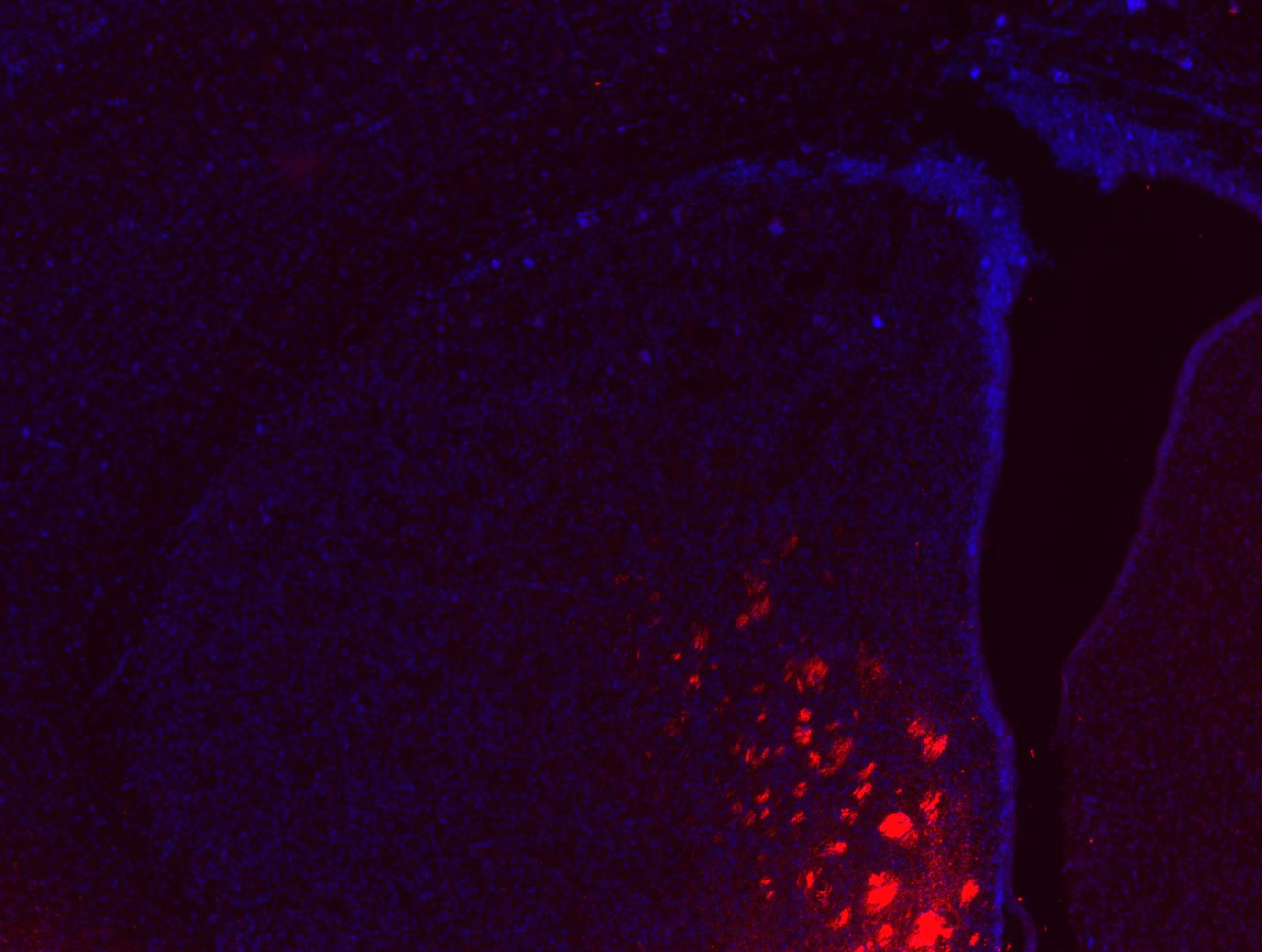

Supplement: Supplementary file 8 — Source data Fig. 5 [file 44319_2026_798_MOESM8_ESM.zip › Figure 5 (V)/5B/DAPI+RFP caudal striatum( Rbp4-cre 0823-6 ctrl).tif]

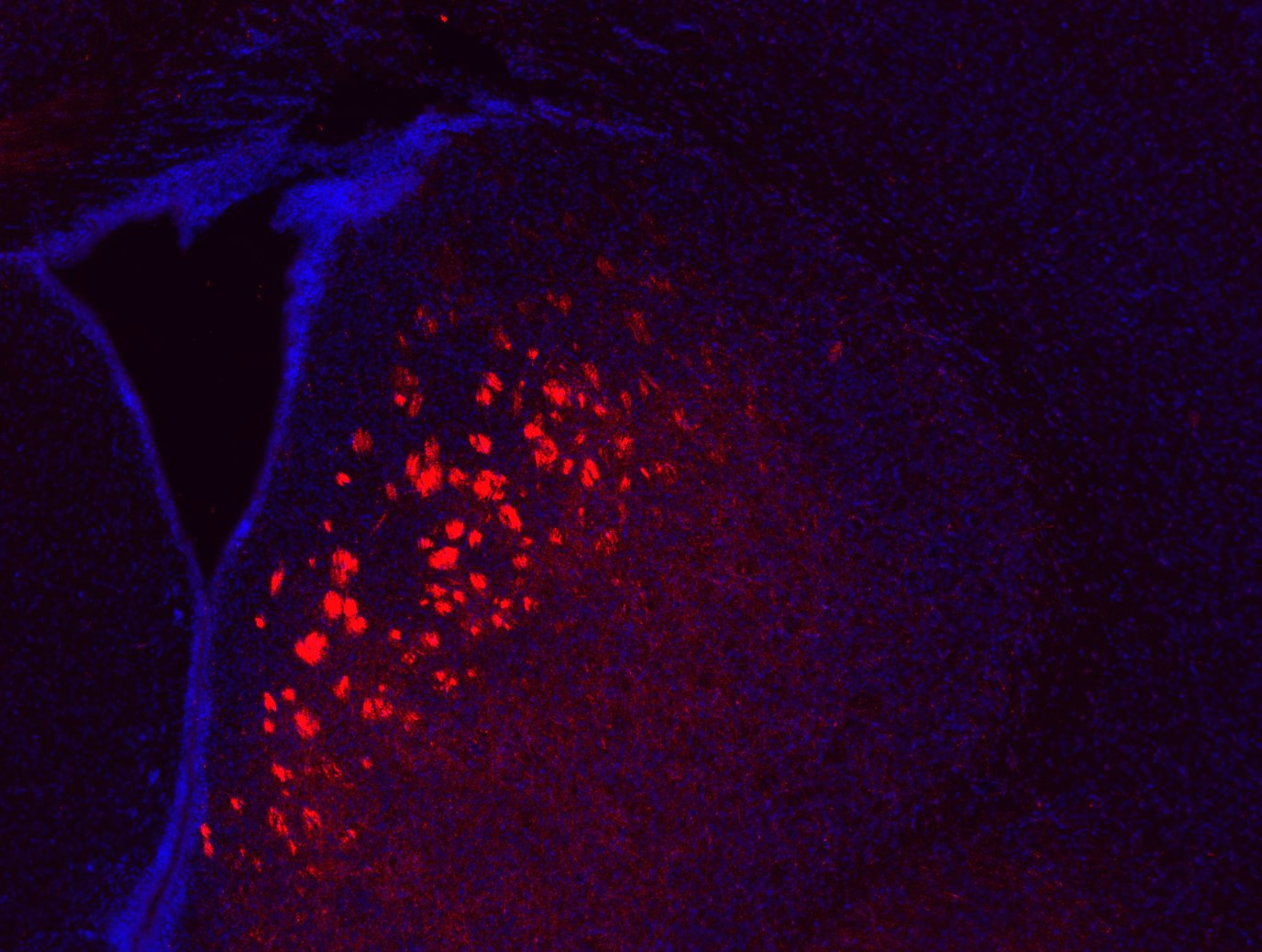

Supplement: Supplementary file 8 — Source data Fig. 5 [file 44319_2026_798_MOESM8_ESM.zip › Figure 5 (V)/5B/DAPI+RFP middle2 striatum( Rbp4-cre 0823-6 ctrl).tif]

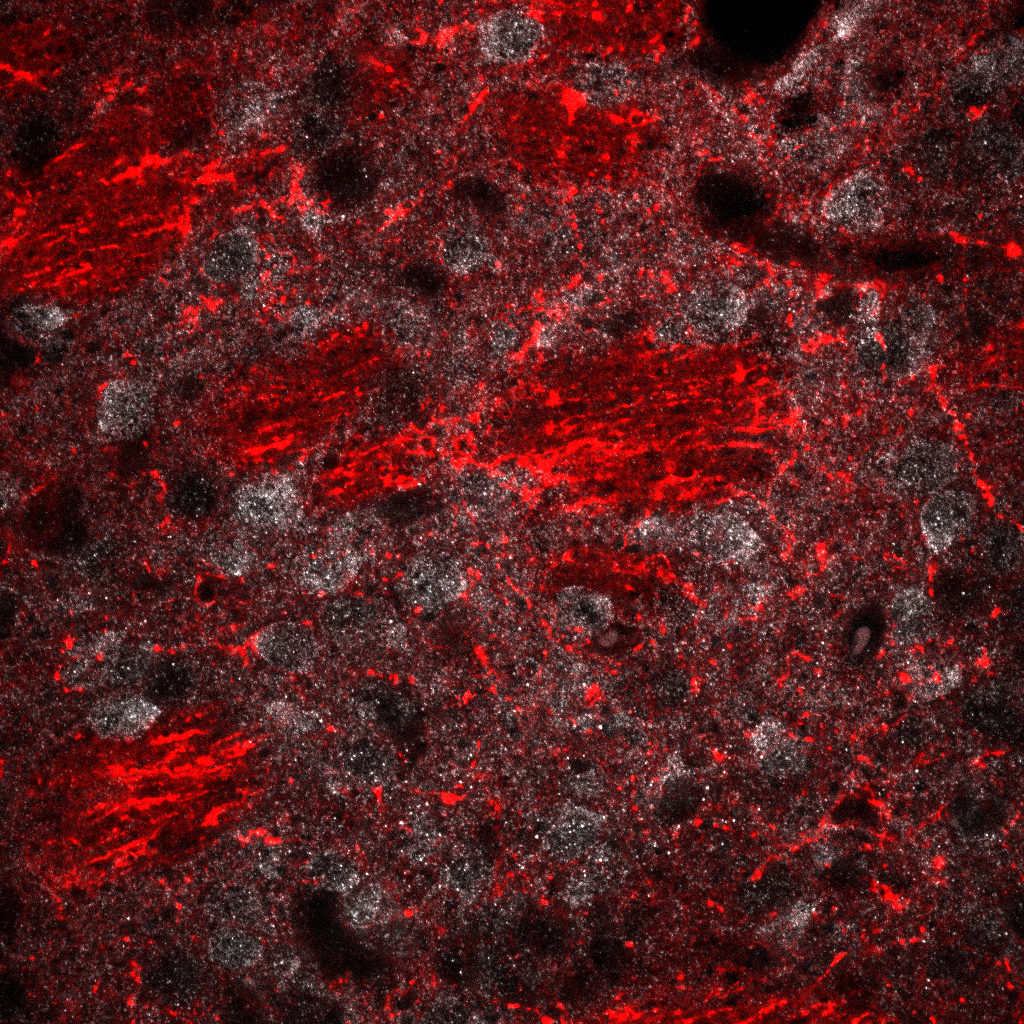

Supplement: Supplementary file 8 — Source data Fig. 5 [file 44319_2026_798_MOESM8_ESM.zip › Figure 5 (V)/5C/5C1_0810-8_2-3-1-Orthogonal Projection-06-Image Export-06_c1-2c1+2.tif]

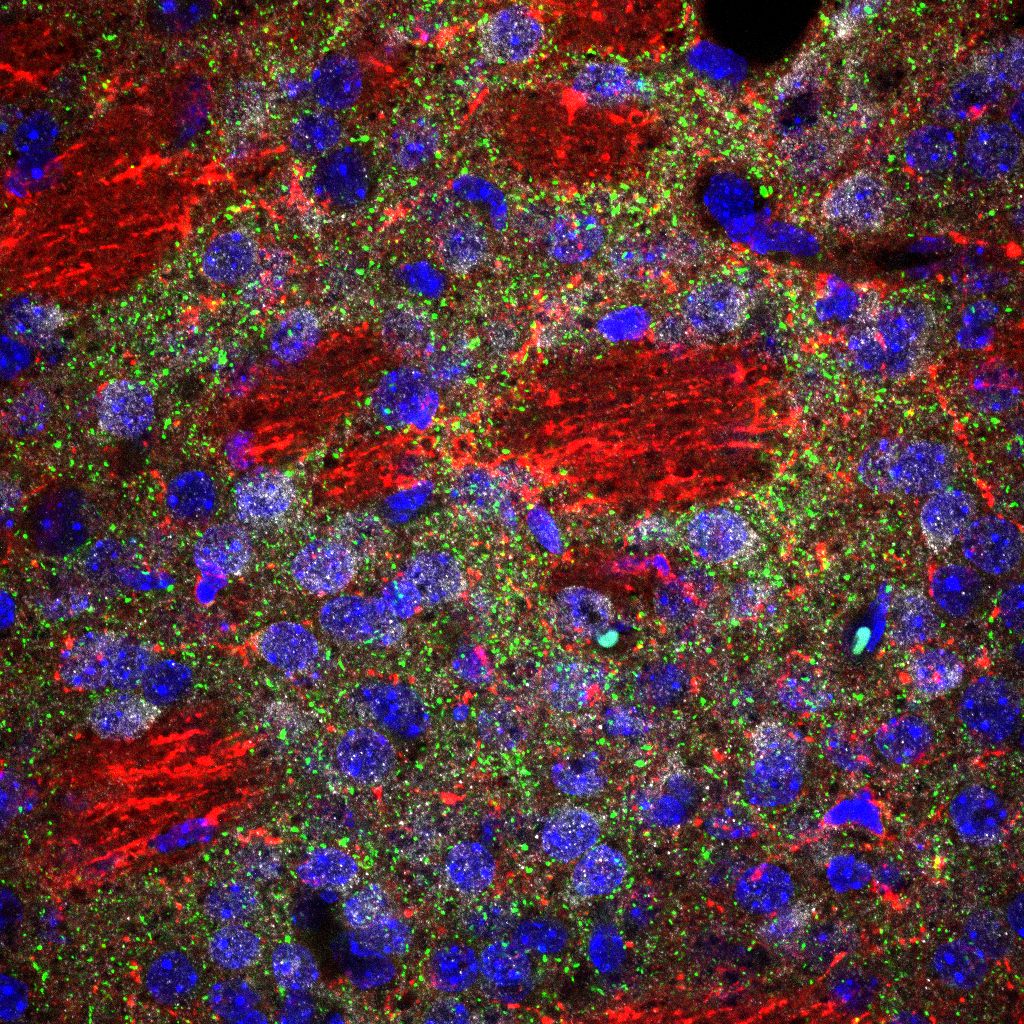

Supplement: Supplementary file 8 — Source data Fig. 5 [file 44319_2026_798_MOESM8_ESM.zip › Figure 5 (V)/5C/5C1_0810-8_2-3-1-Orthogonal Projection-06-Image Export-10_c1+2+3+4.tif]

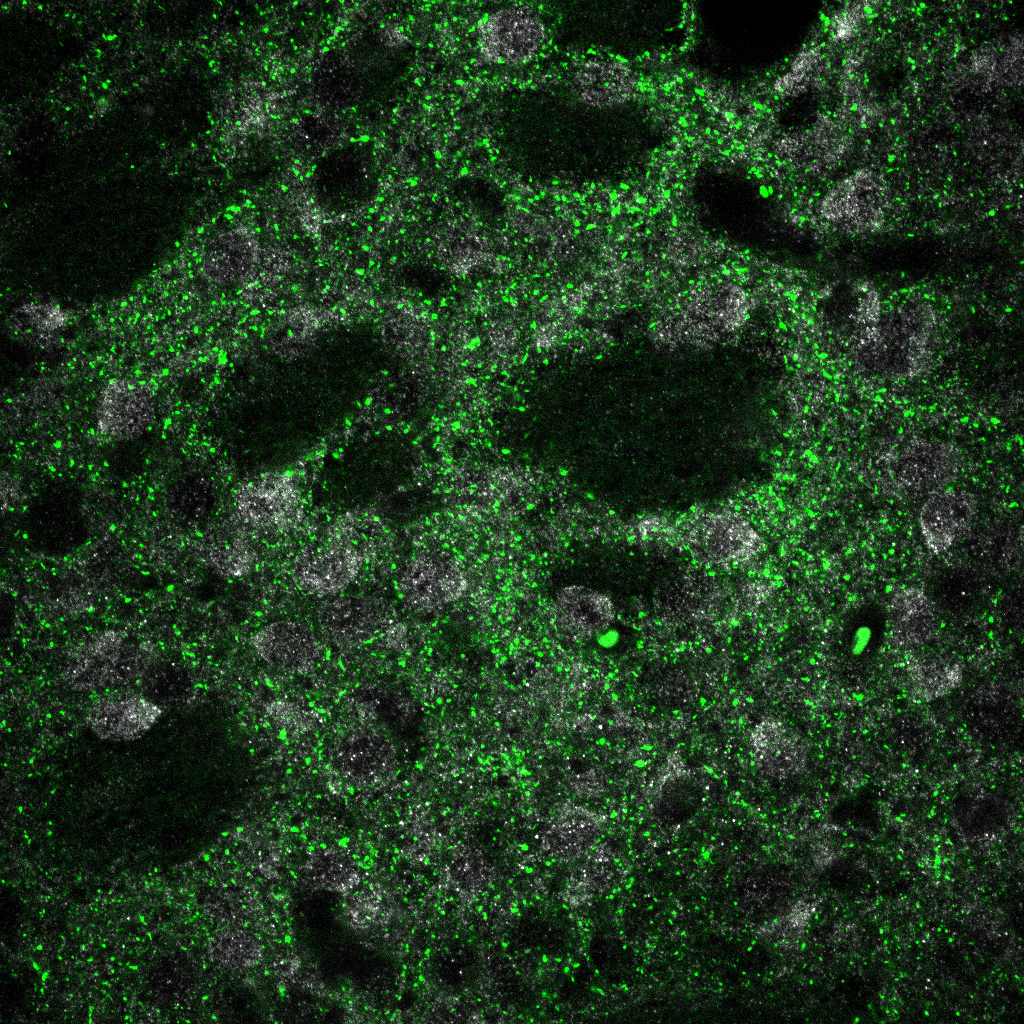

Supplement: Supplementary file 8 — Source data Fig. 5 [file 44319_2026_798_MOESM8_ESM.zip › Figure 5 (V)/5C/5C1_0810-8_2-3-1-Orthogonal Projection-06-Image Export-10_c1+3.tif]

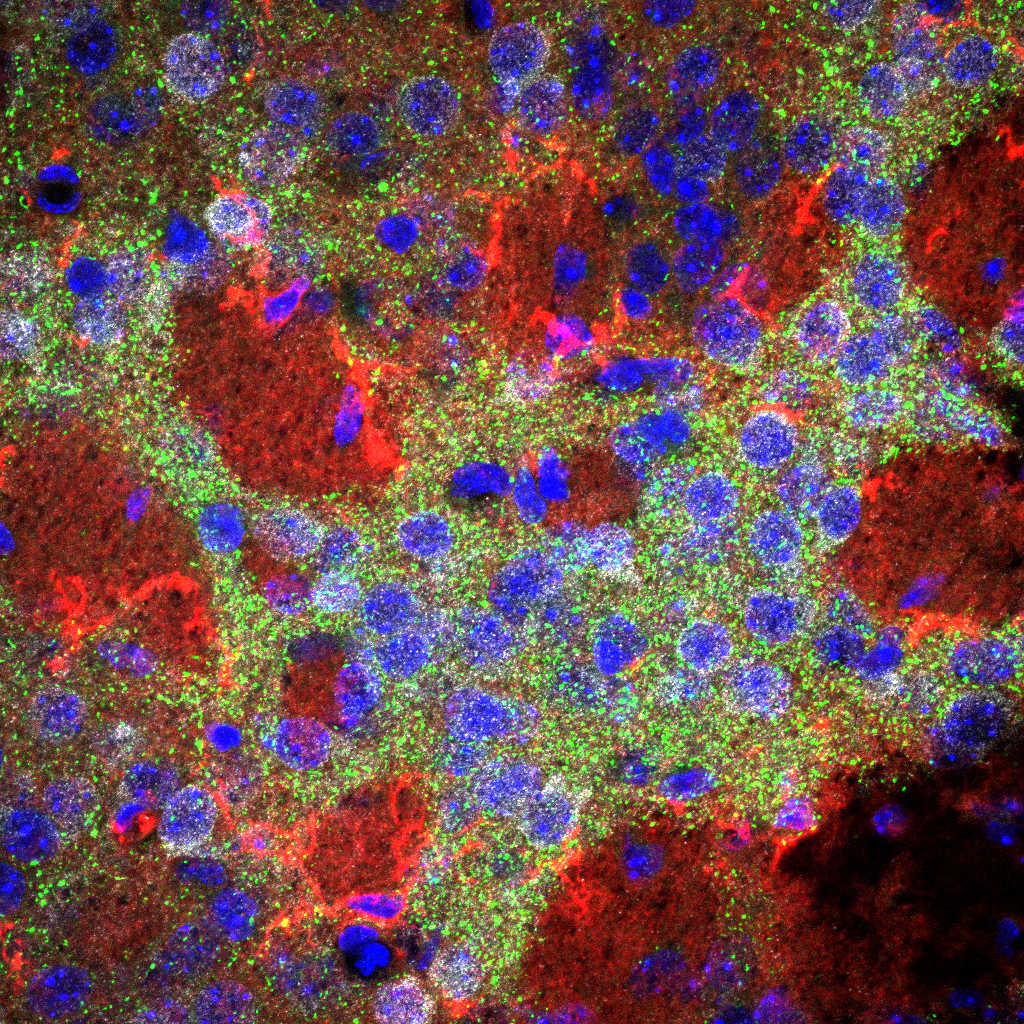

Supplement: Supplementary file 8 — Source data Fig. 5 [file 44319_2026_798_MOESM8_ESM.zip › Figure 5 (V)/5C/5C2_0904-3_4-4-3-Orthogonal Projection-19-Image Export-04_c1+2+3+4.tif]

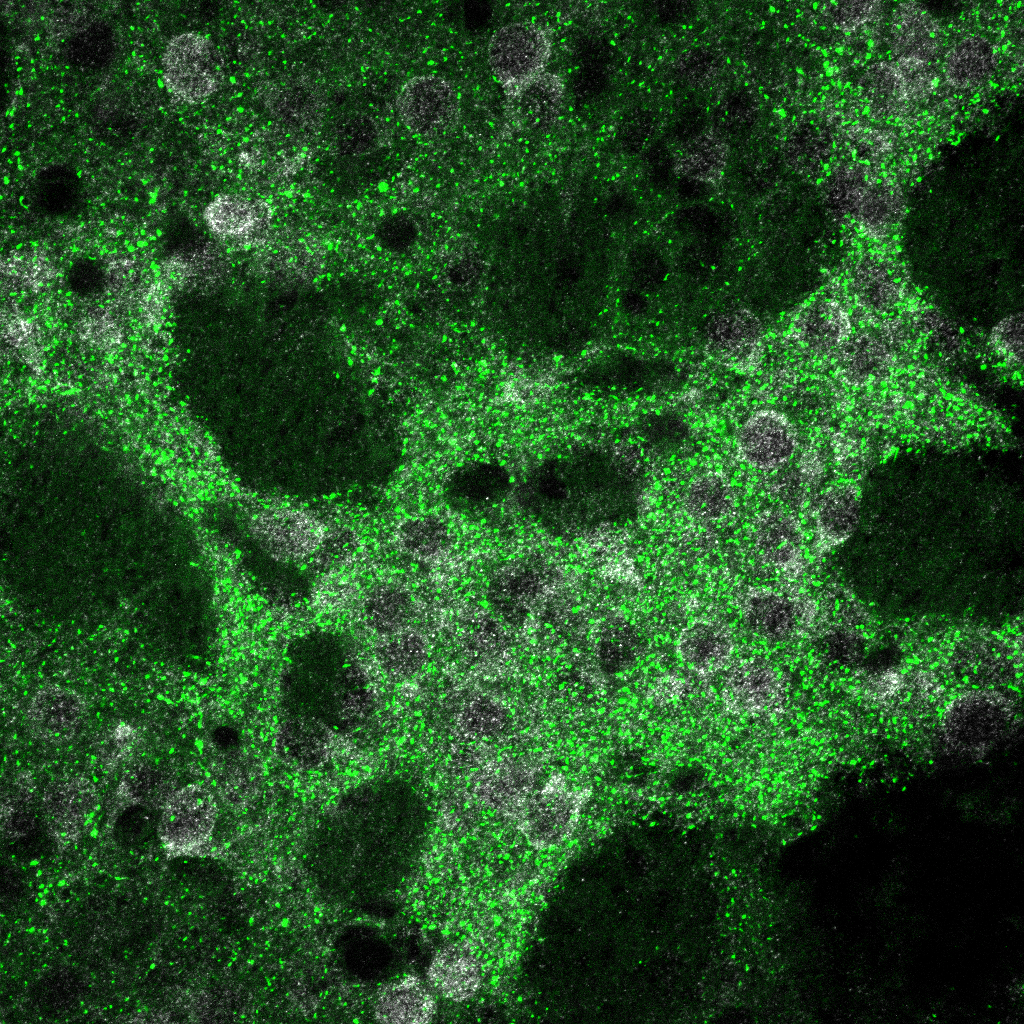

Supplement: Supplementary file 8 — Source data Fig. 5 [file 44319_2026_798_MOESM8_ESM.zip › Figure 5 (V)/5C/5C2_0904-3_4-4-3-Orthogonal Projection-19-Image Export-04_c1+3.tif]

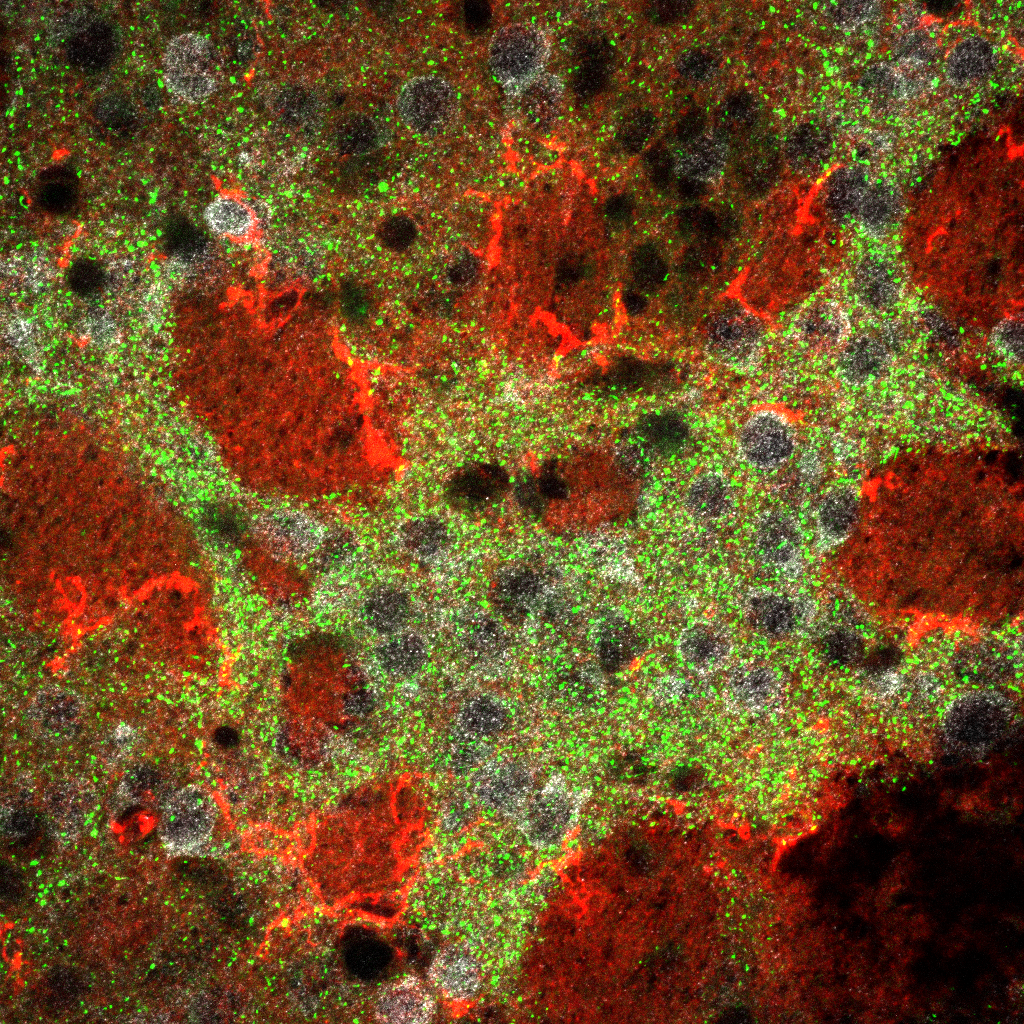

Supplement: Supplementary file 8 — Source data Fig. 5 [file 44319_2026_798_MOESM8_ESM.zip › Figure 5 (V)/5C/5C2_0904-3_4-4-3-Orthogonal Projection-19-Image Export-04_c1-3c1+2+3.tif]

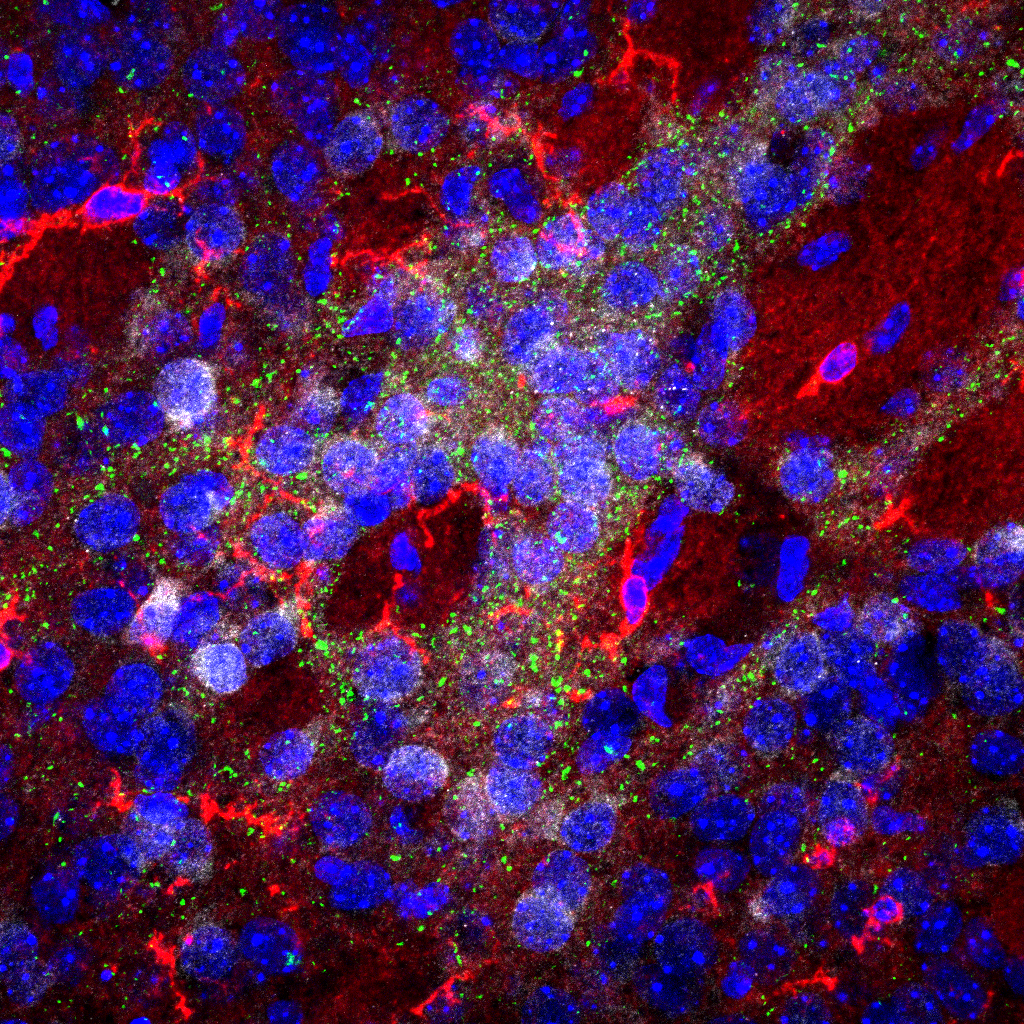

Supplement: Supplementary file 8 — Source data Fig. 5 [file 44319_2026_798_MOESM8_ESM.zip › Figure 5 (V)/5C/5C3_0331-7-2-2-Orthogonal Projection-04-Image Export-06_c1+2+3+4.tif]

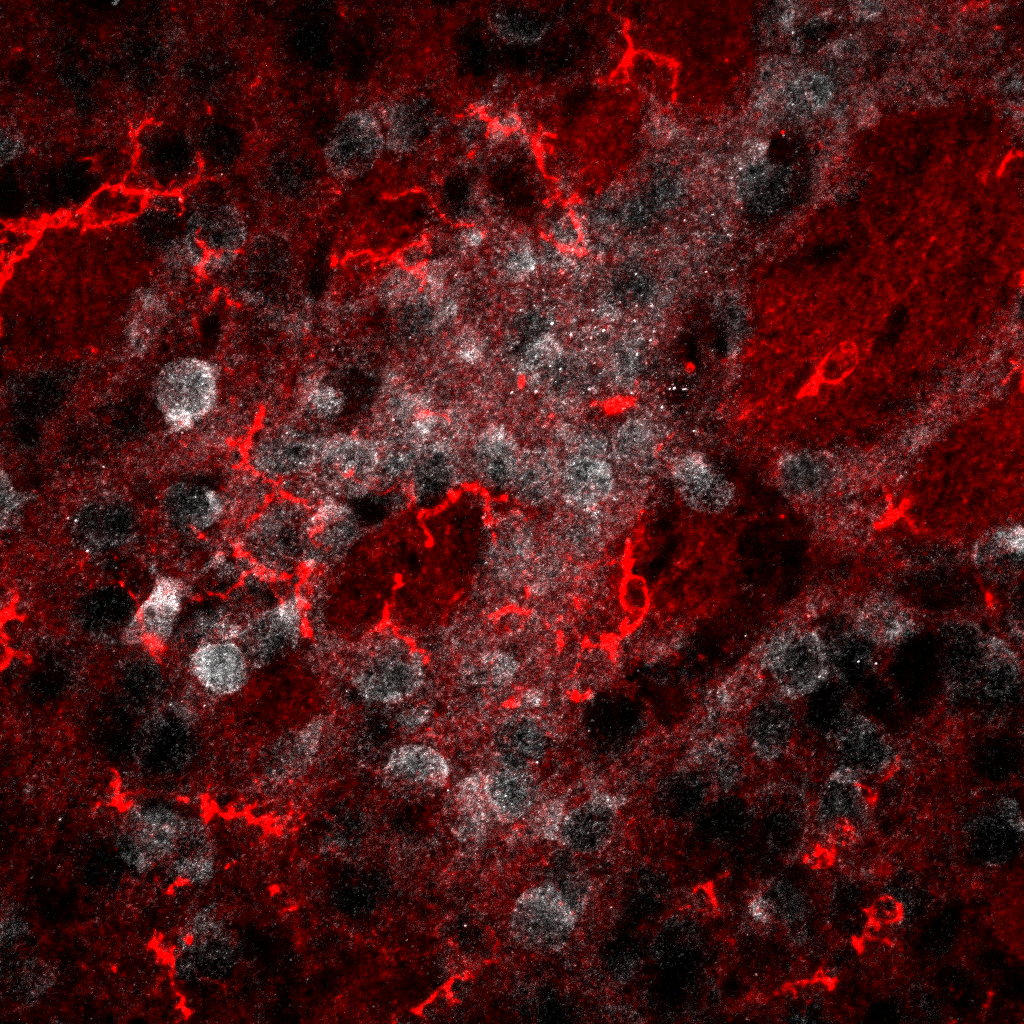

Supplement: Supplementary file 8 — Source data Fig. 5 [file 44319_2026_798_MOESM8_ESM.zip › Figure 5 (V)/5C/5C3_0331-7-2-2-Orthogonal Projection-04-Image Export-06_c1+2.tif]

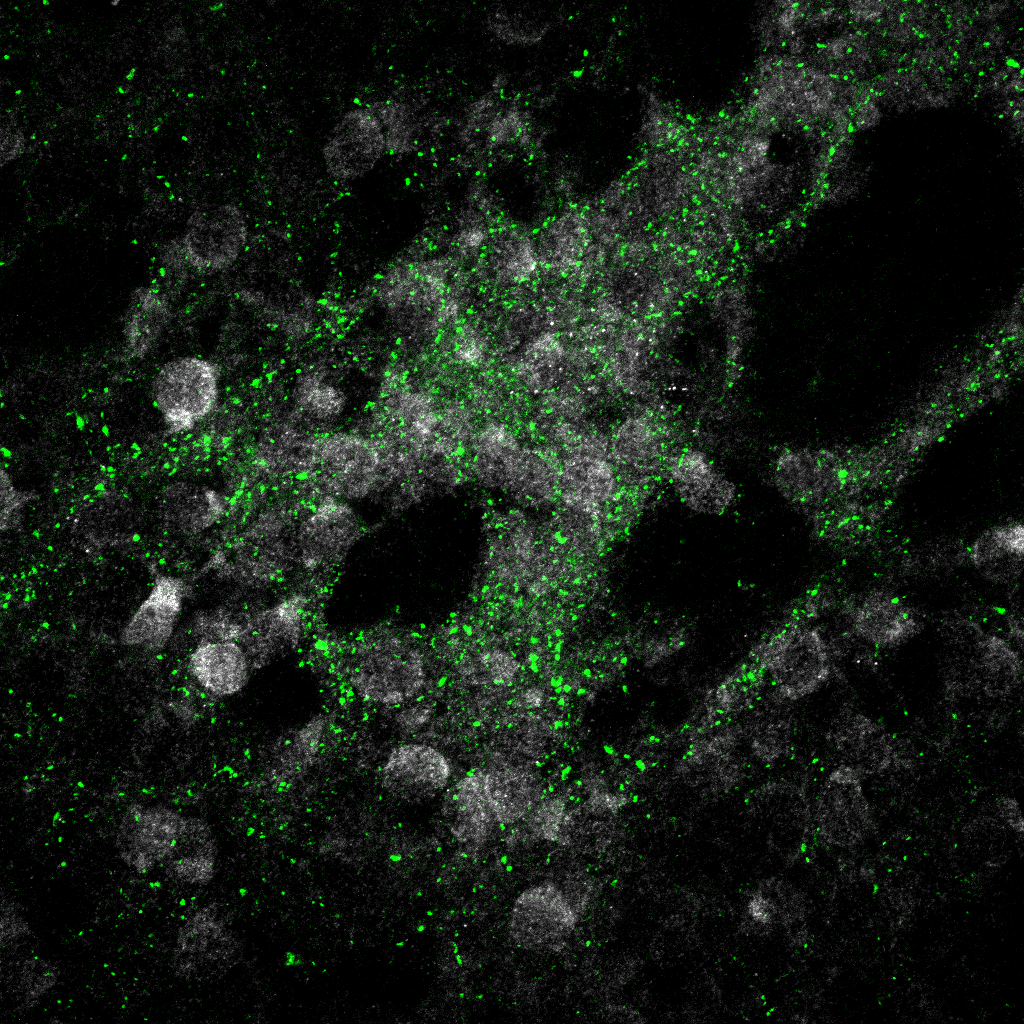

Supplement: Supplementary file 8 — Source data Fig. 5 [file 44319_2026_798_MOESM8_ESM.zip › Figure 5 (V)/5C/5C3_0331-7-2-2-Orthogonal Projection-04-Image Export-06_c1+3.tif]

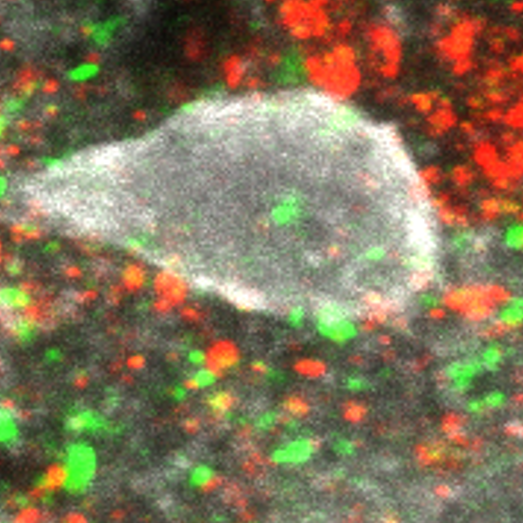

Supplement: Supplementary file 8 — Source data Fig. 5 [file 44319_2026_798_MOESM8_ESM.zip › Figure 5 (V)/5D/5D1.tif]

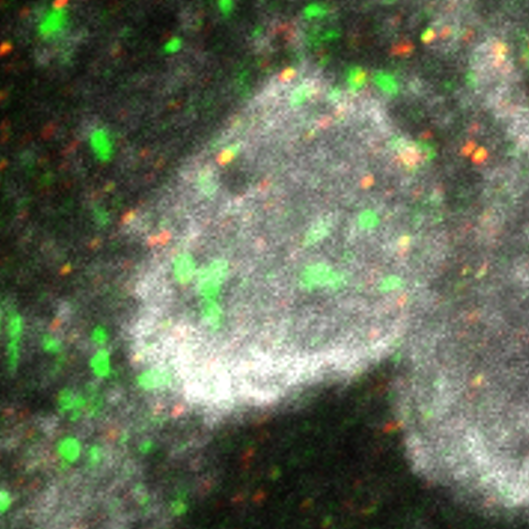

Supplement: Supplementary file 8 — Source data Fig. 5 [file 44319_2026_798_MOESM8_ESM.zip › Figure 5 (V)/5D/5D2.tif]

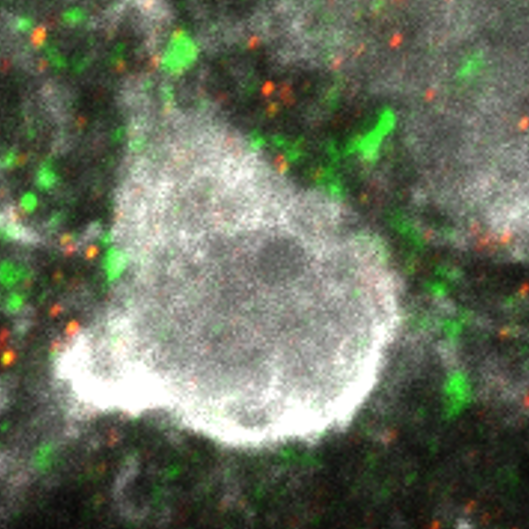

Supplement: Supplementary file 8 — Source data Fig. 5 [file 44319_2026_798_MOESM8_ESM.zip › Figure 5 (V)/5D/5D3.tif]

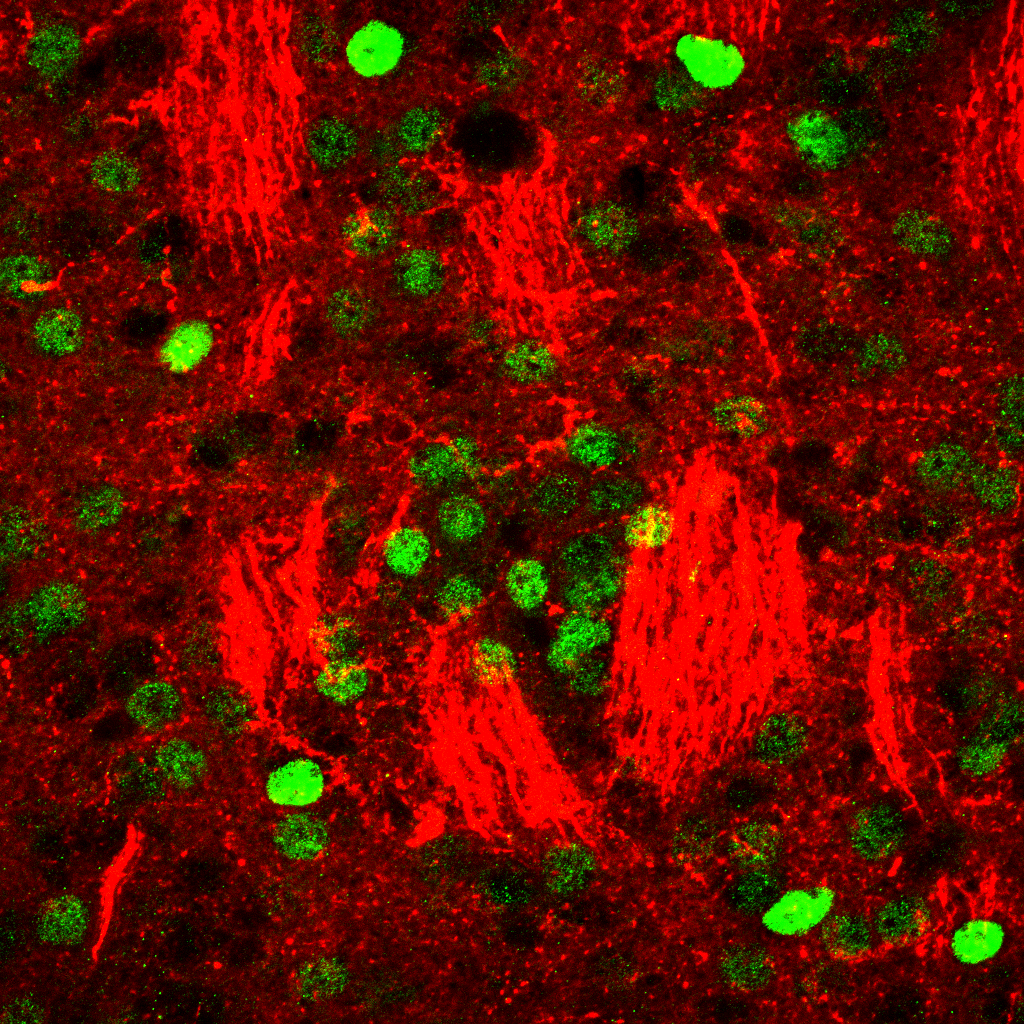

Supplement: Supplementary file 9 — Source data Fig. 6 [file 44319_2026_798_MOESM9_ESM.zip › Figure 6 (V)/6B/6B'.tif]

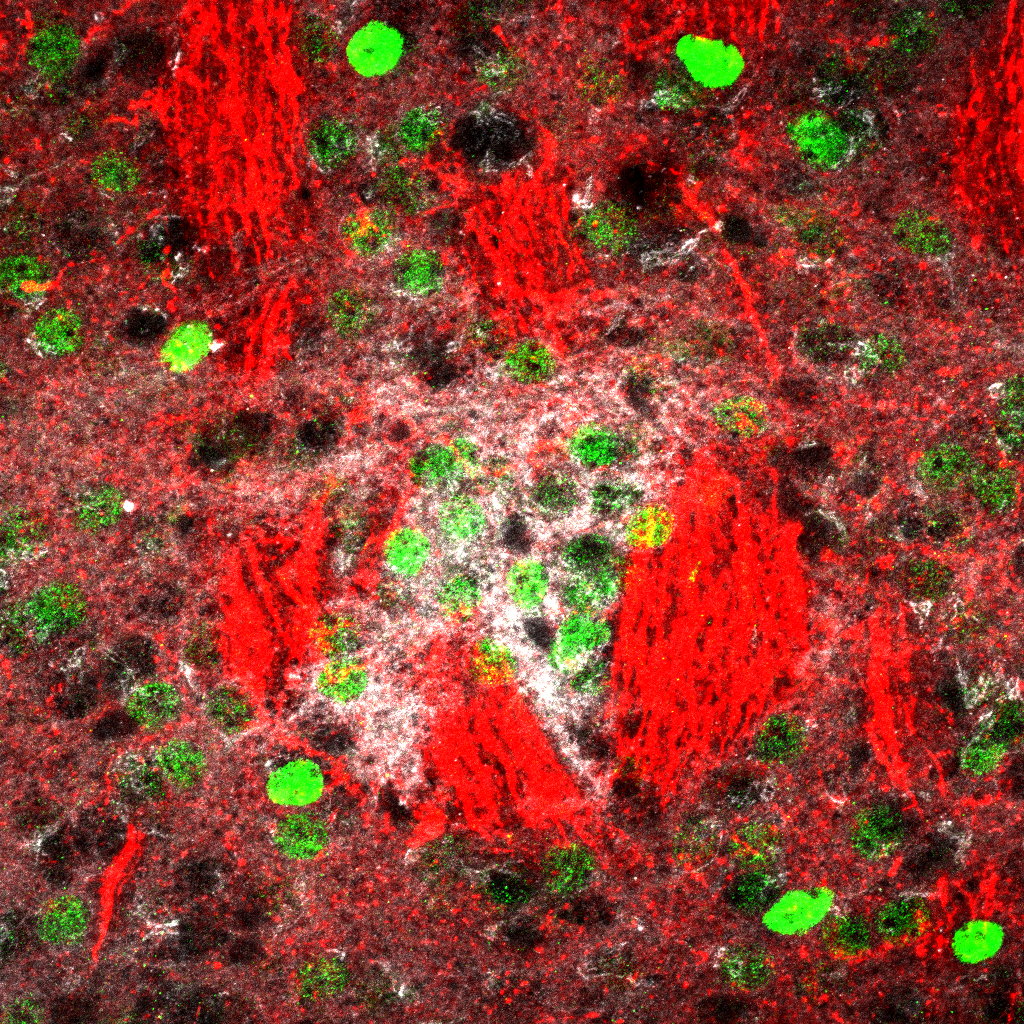

Supplement: Supplementary file 9 — Source data Fig. 6 [file 44319_2026_798_MOESM9_ESM.zip › Figure 6 (V)/6B/6B.tif]

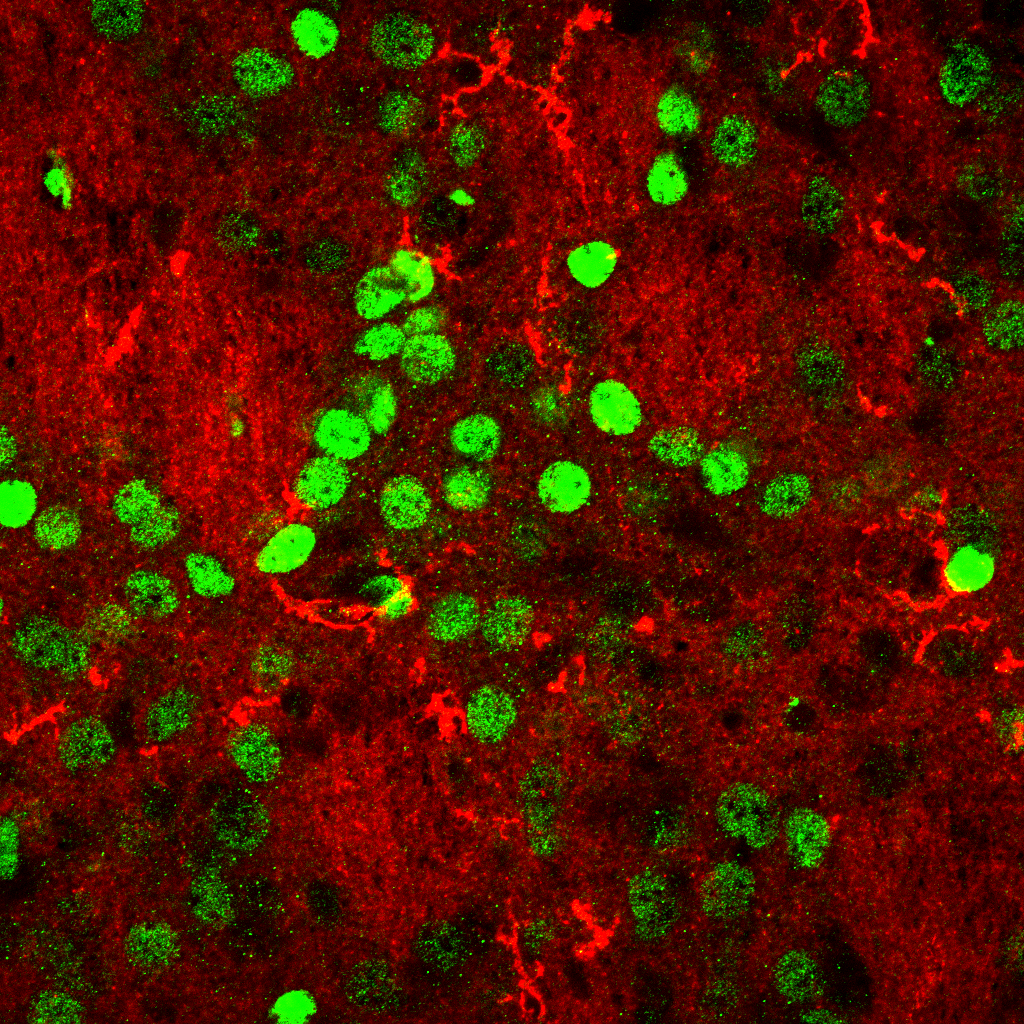

Supplement: Supplementary file 9 — Source data Fig. 6 [file 44319_2026_798_MOESM9_ESM.zip › Figure 6 (V)/6C/2_0331-1-4-2-Orthogonal Projection-03-Image Export-14_c2-2c2+3.tif]

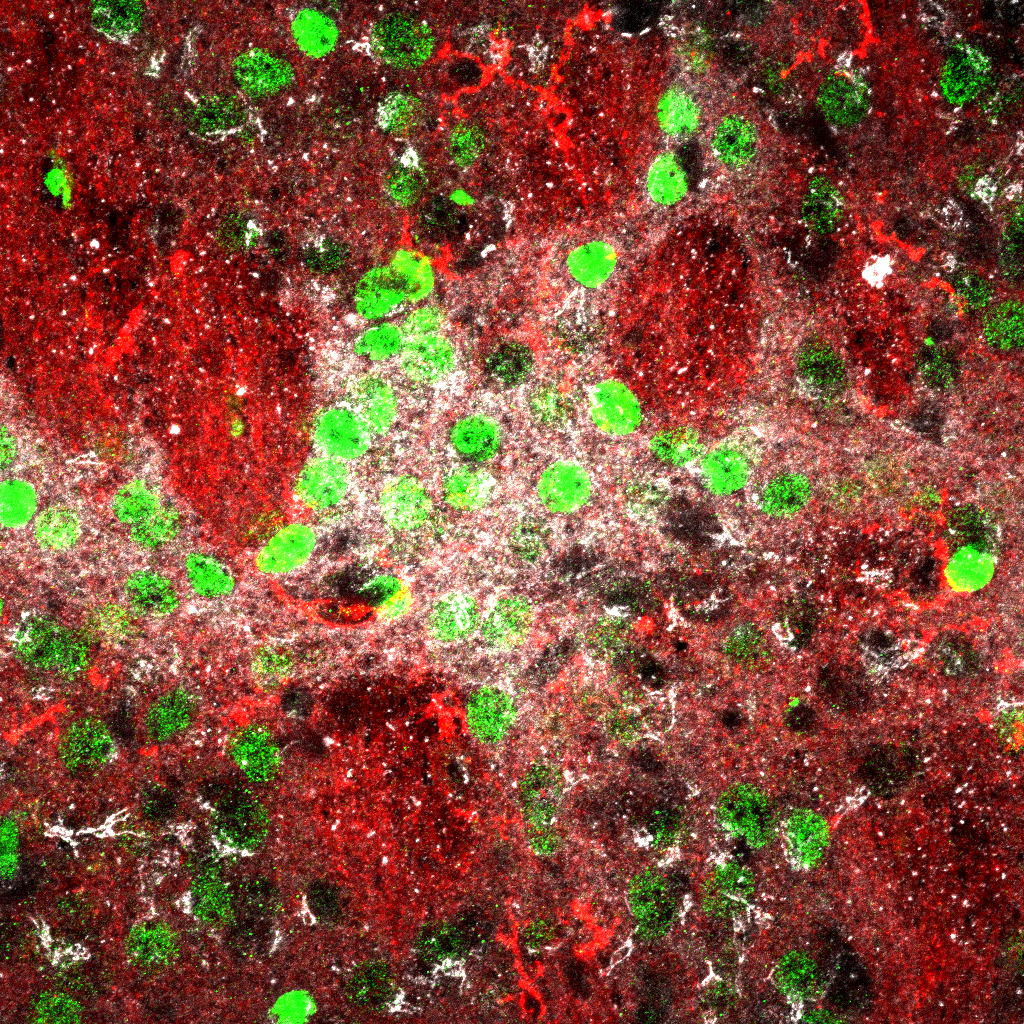

Supplement: Supplementary file 9 — Source data Fig. 6 [file 44319_2026_798_MOESM9_ESM.zip › Figure 6 (V)/6C/6C.tif]

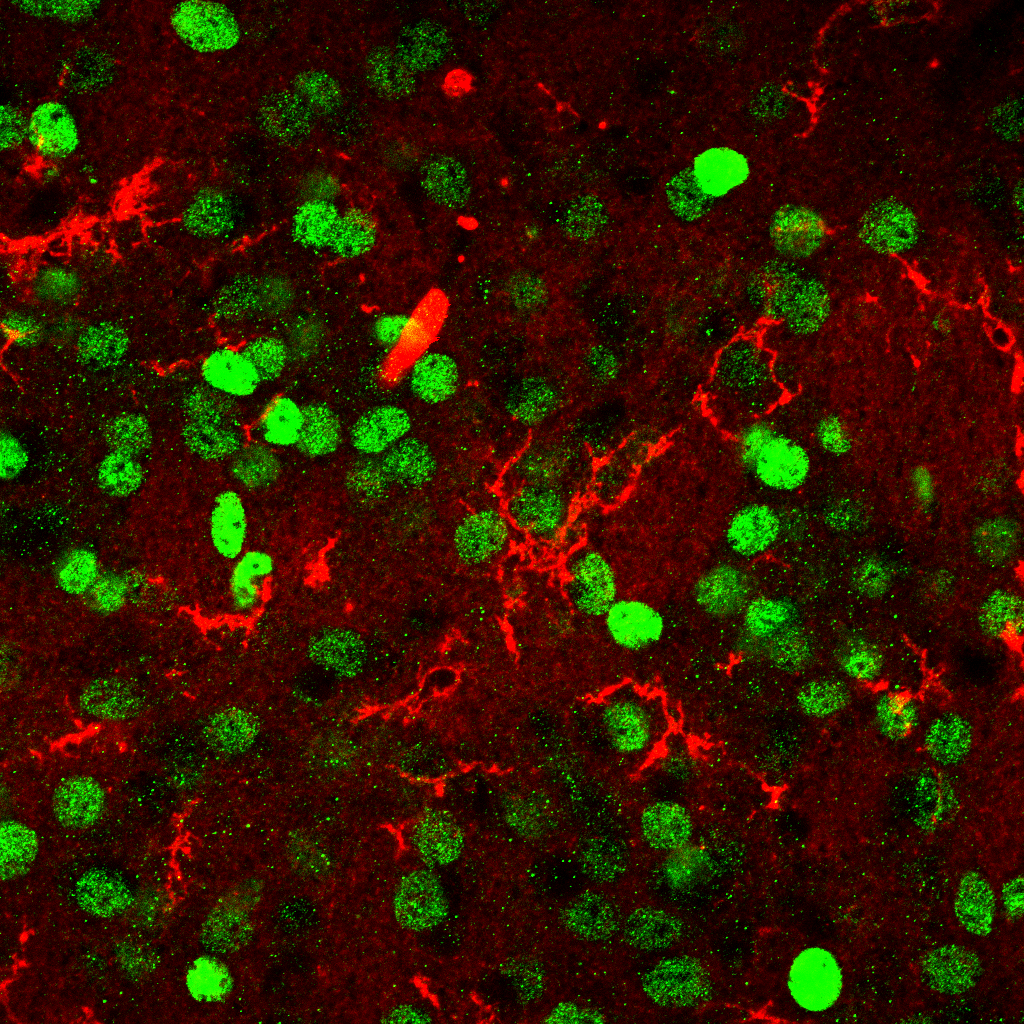

Supplement: Supplementary file 9 — Source data Fig. 6 [file 44319_2026_798_MOESM9_ESM.zip › Figure 6 (V)/6D/6D'.tif]

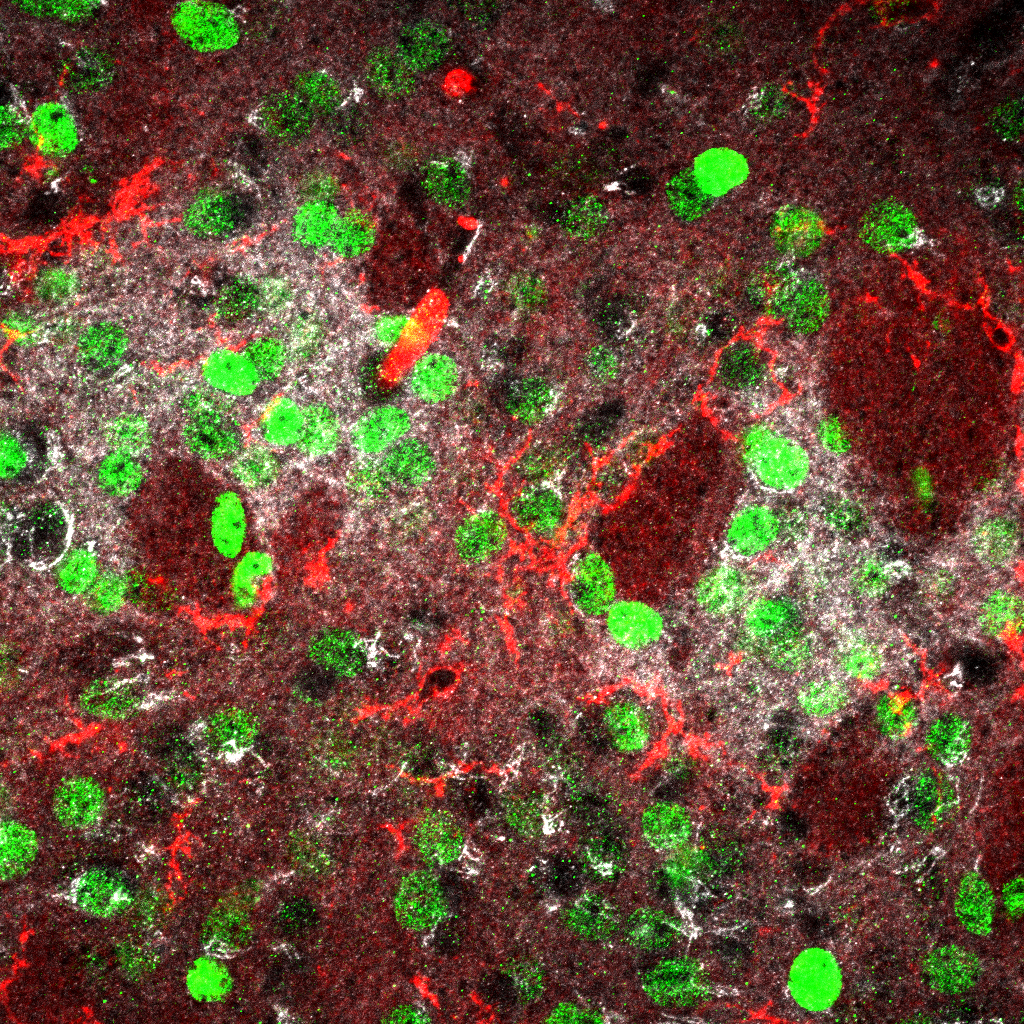

Supplement: Supplementary file 9 — Source data Fig. 6 [file 44319_2026_798_MOESM9_ESM.zip › Figure 6 (V)/6D/6D.tif]

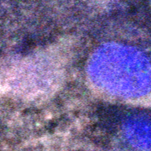

Supplement: Supplementary file 9 — Source data Fig. 6 [file 44319_2026_798_MOESM9_ESM.zip › Figure 6 (V)/6H/6H''.tif]

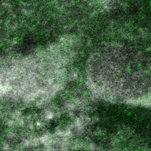

Supplement: Supplementary file 9 — Source data Fig. 6 [file 44319_2026_798_MOESM9_ESM.zip › Figure 6 (V)/6H/6H'.tif]

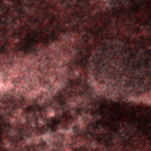

Supplement: Supplementary file 9 — Source data Fig. 6 [file 44319_2026_798_MOESM9_ESM.zip › Figure 6 (V)/6H/6H.tif]

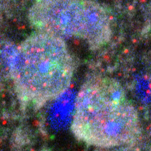

Supplement: Supplementary file 9 — Source data Fig. 6 [file 44319_2026_798_MOESM9_ESM.zip › Figure 6 (V)/6I/6I''.tif]

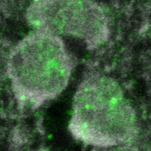

Supplement: Supplementary file 9 — Source data Fig. 6 [file 44319_2026_798_MOESM9_ESM.zip › Figure 6 (V)/6I/6I'.tif]

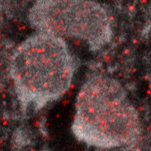

Supplement: Supplementary file 9 — Source data Fig. 6 [file 44319_2026_798_MOESM9_ESM.zip › Figure 6 (V)/6I/6I.tif]

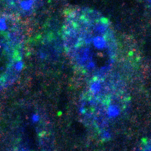

Supplement: Supplementary file 9 — Source data Fig. 6 [file 44319_2026_798_MOESM9_ESM.zip › Figure 6 (V)/6J/6J''.tif]

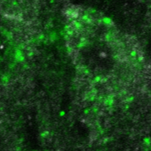

Supplement: Supplementary file 9 — Source data Fig. 6 [file 44319_2026_798_MOESM9_ESM.zip › Figure 6 (V)/6J/6J'.tif]

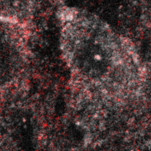

Supplement: Supplementary file 9 — Source data Fig. 6 [file 44319_2026_798_MOESM9_ESM.zip › Figure 6 (V)/6J/6J.tif]

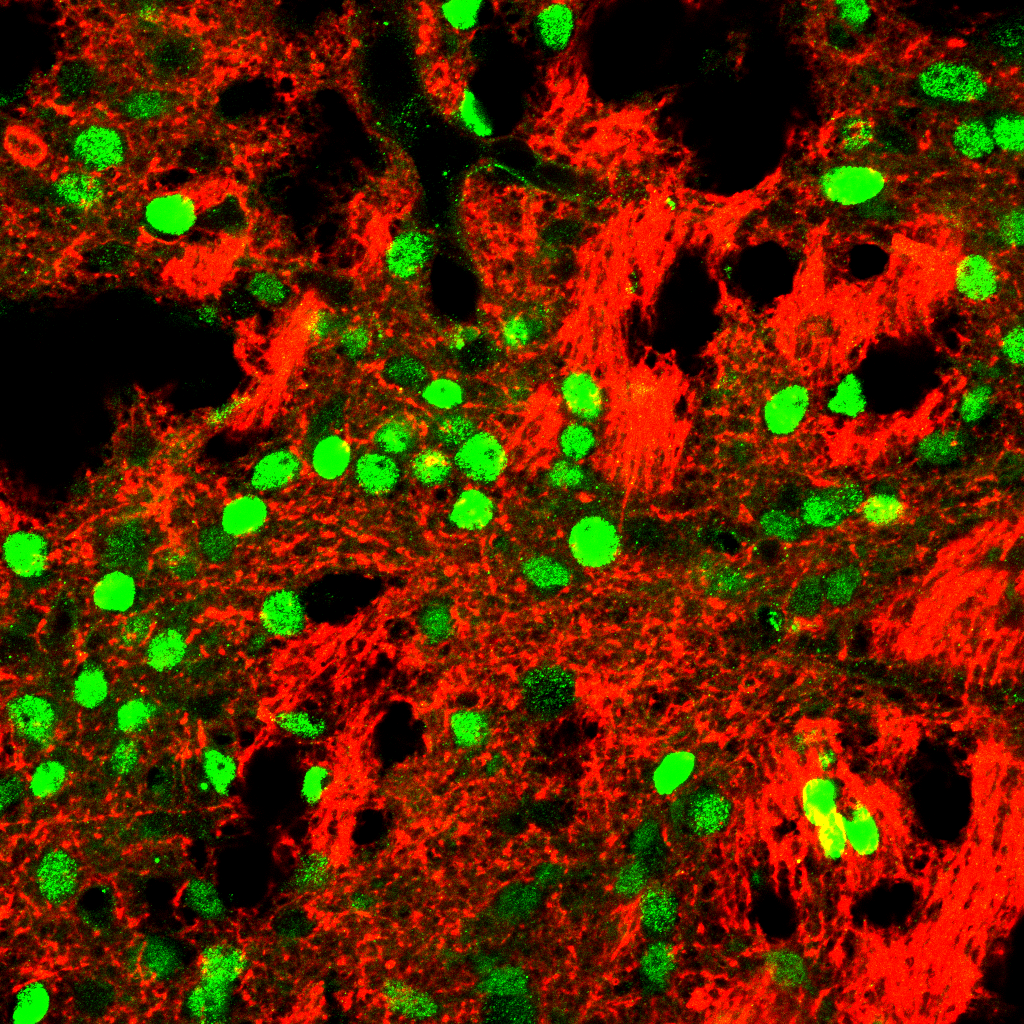

Supplement: Supplementary file 10 — Source data Fig. 7 [file 44319_2026_798_MOESM10_ESM.zip › Figure 7 (V)/7G/7G'.tif]

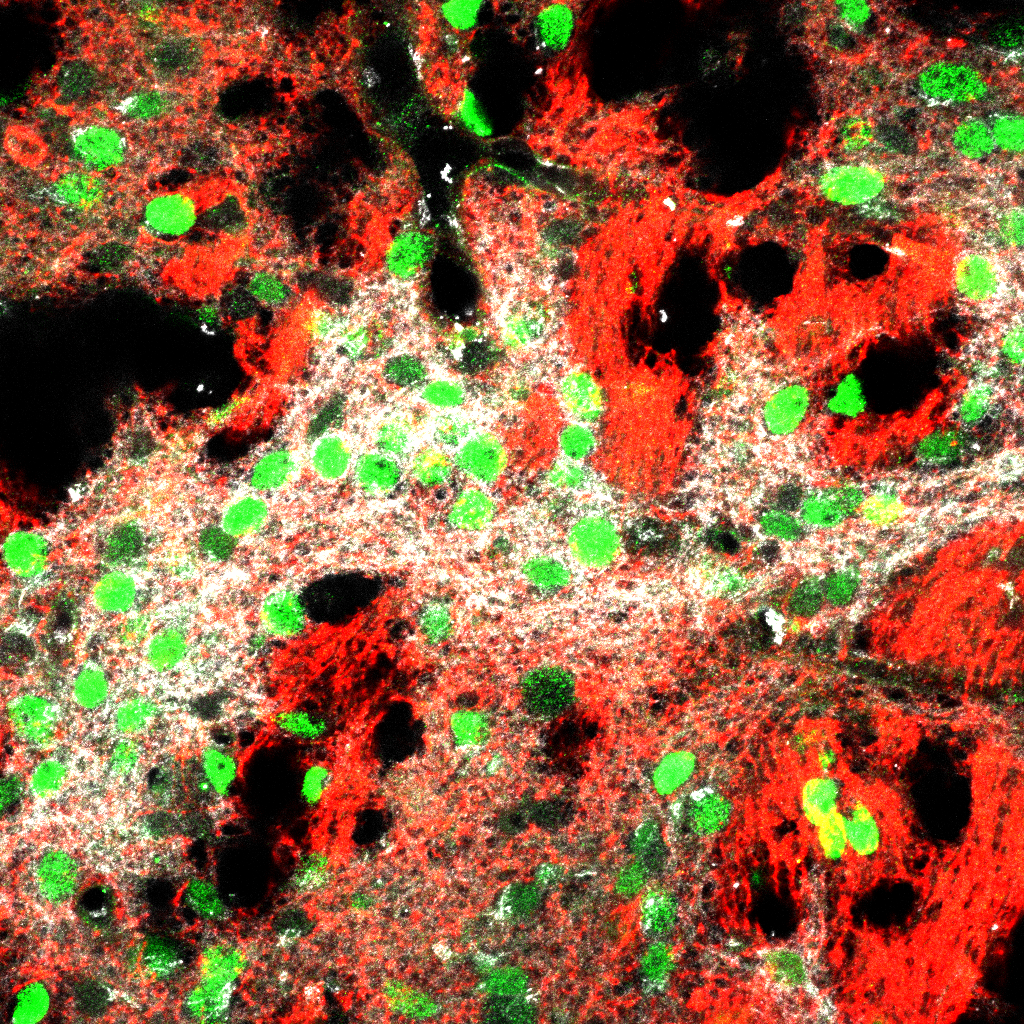

Supplement: Supplementary file 10 — Source data Fig. 7 [file 44319_2026_798_MOESM10_ESM.zip › Figure 7 (V)/7G/7G.tif]

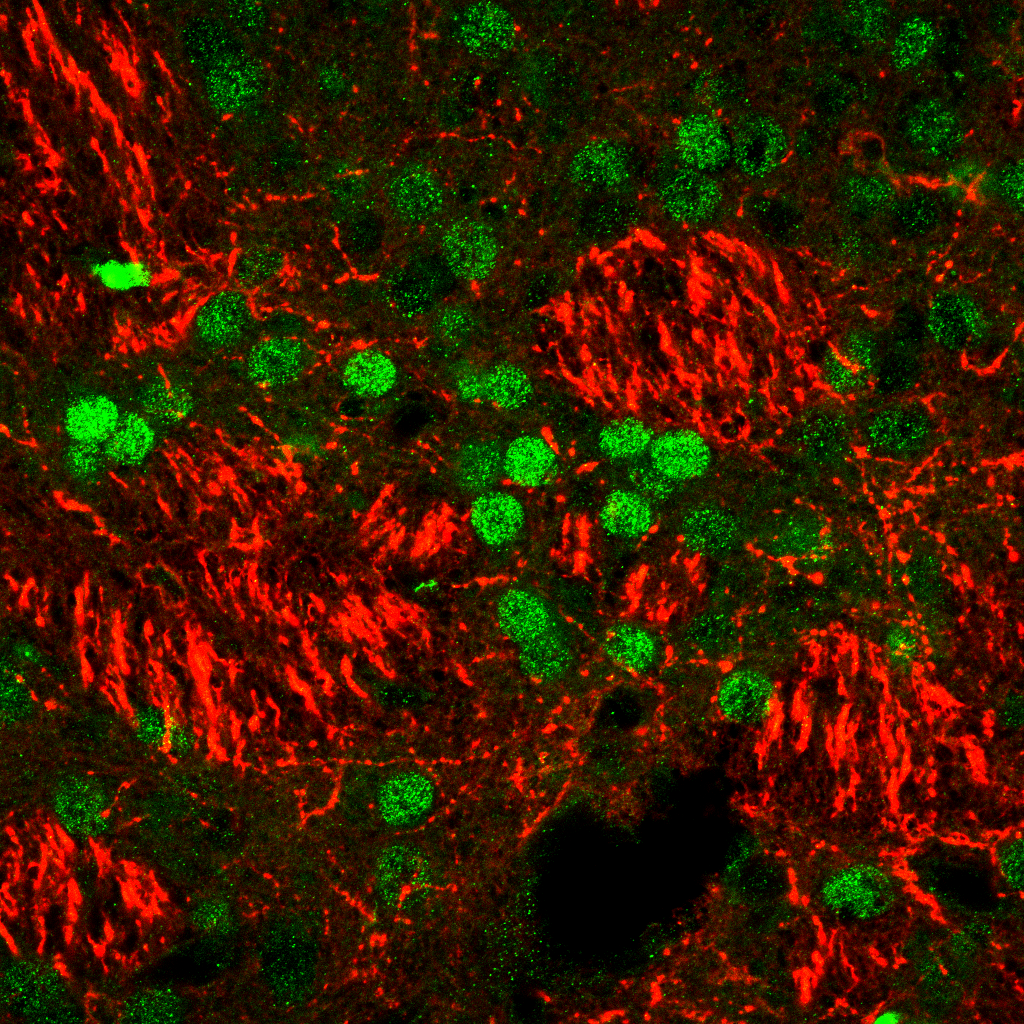

Supplement: Supplementary file 10 — Source data Fig. 7 [file 44319_2026_798_MOESM10_ESM.zip › Figure 7 (V)/7H/7H'.tif]

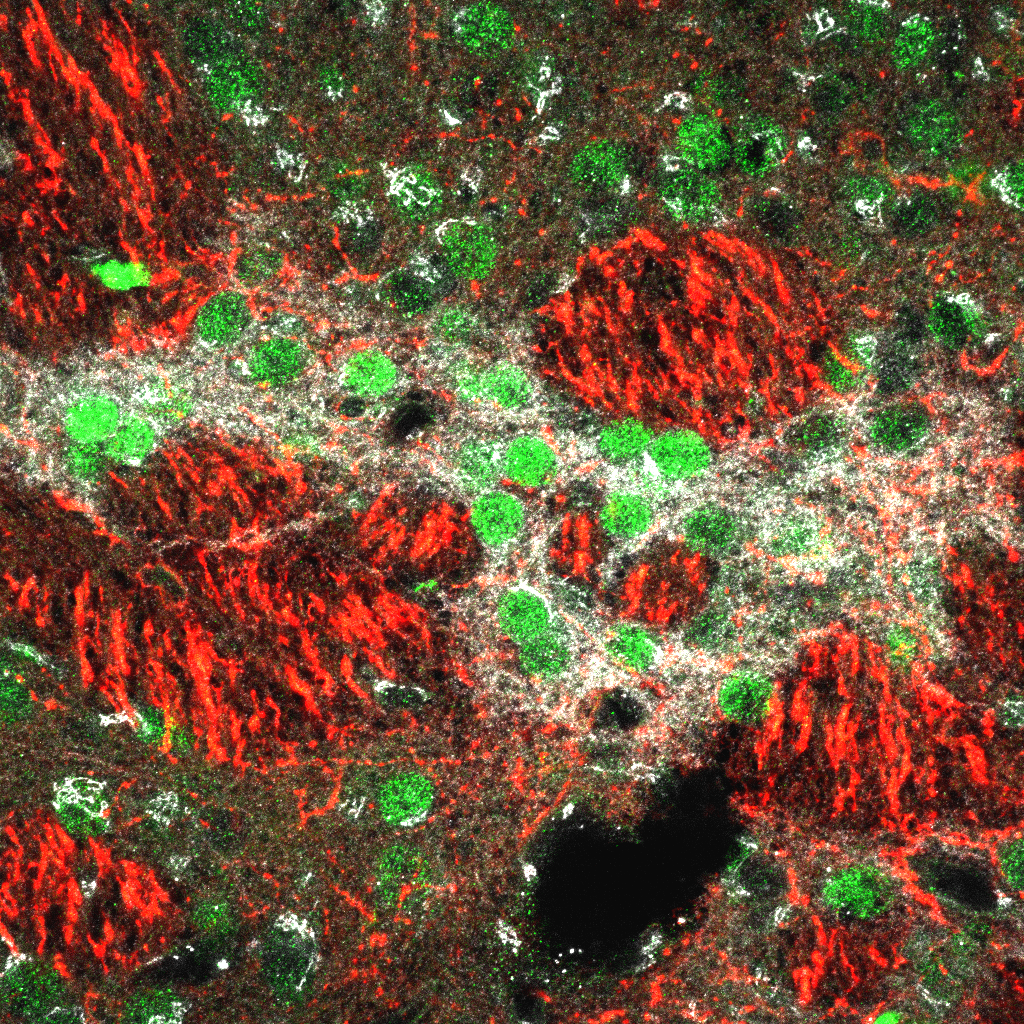

Supplement: Supplementary file 10 — Source data Fig. 7 [file 44319_2026_798_MOESM10_ESM.zip › Figure 7 (V)/7H/7H.tif]

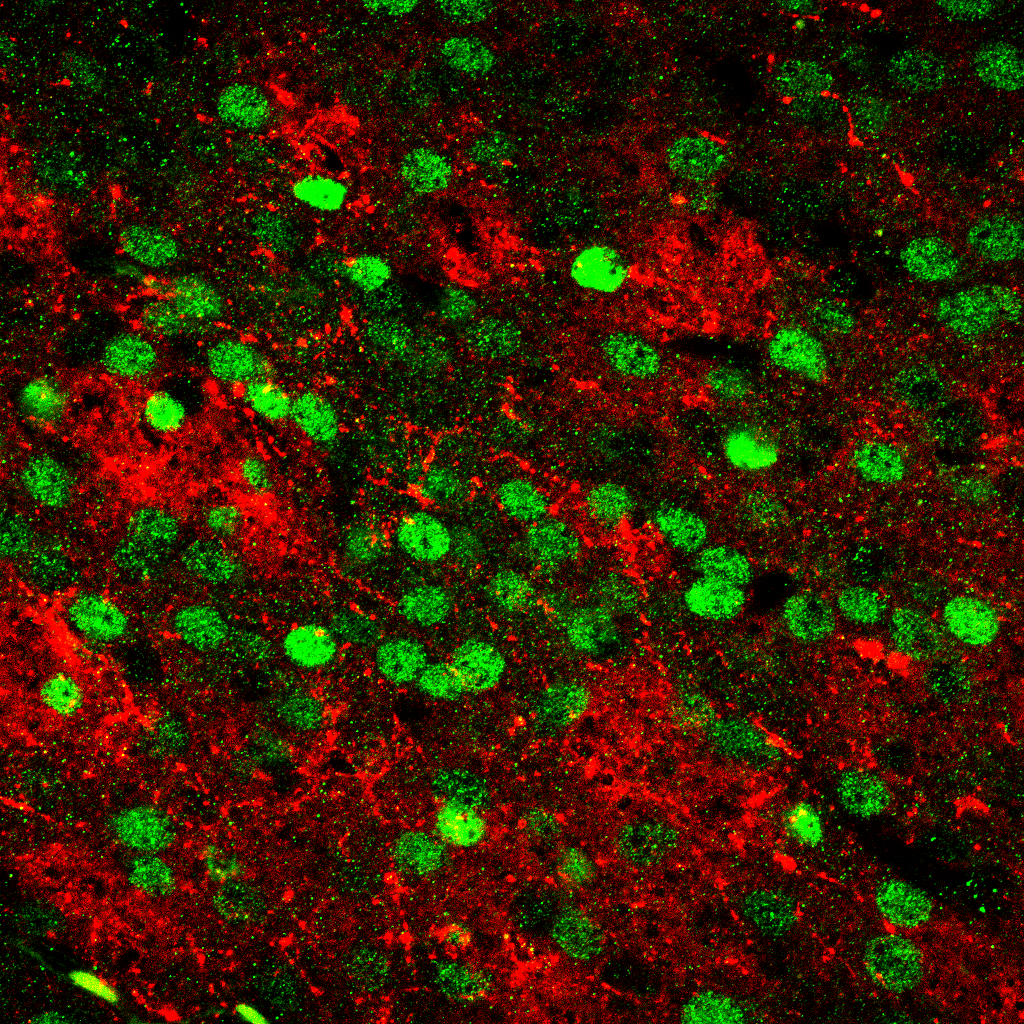

Supplement: Supplementary file 10 — Source data Fig. 7 [file 44319_2026_798_MOESM10_ESM.zip › Figure 7 (V)/7I/7I'.tif]

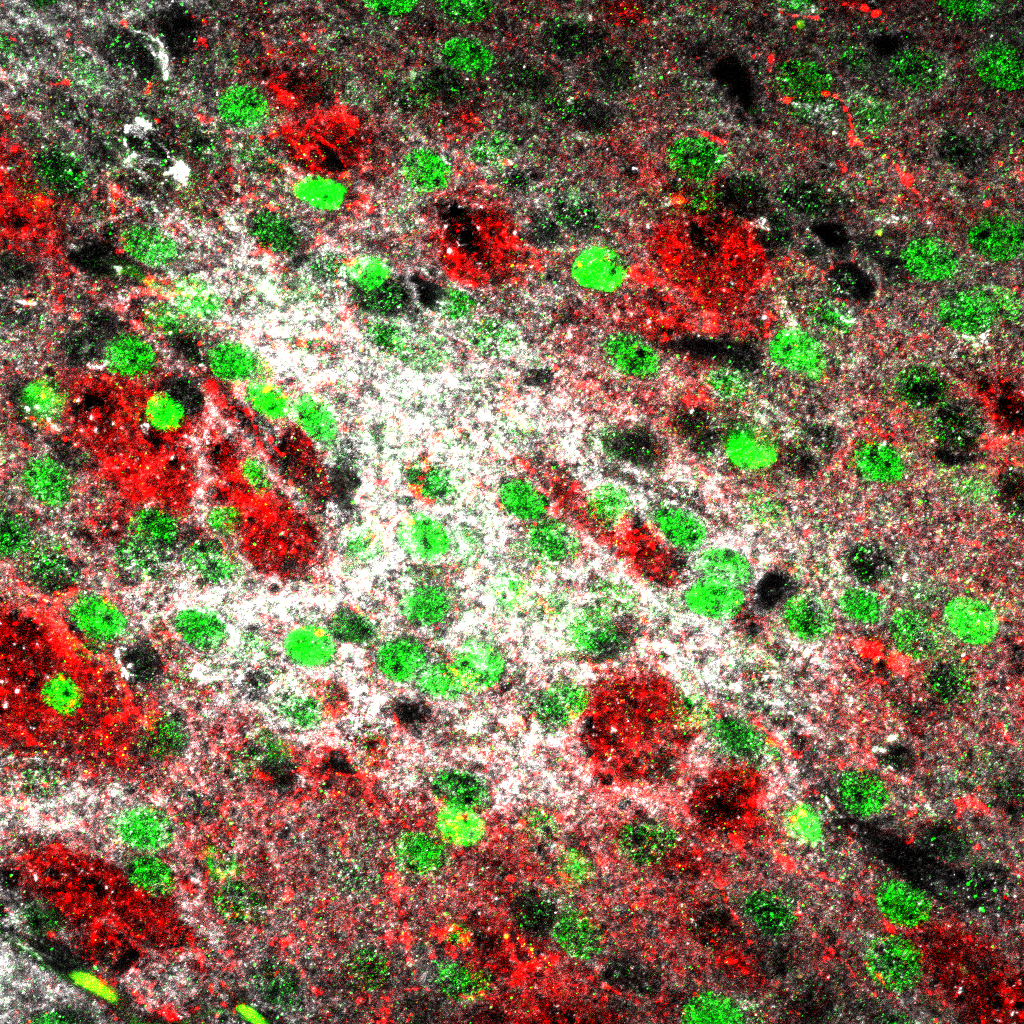

Supplement: Supplementary file 10 — Source data Fig. 7 [file 44319_2026_798_MOESM10_ESM.zip › Figure 7 (V)/7I/Result of Result of 3_0324-5-2-5-Orthogonal Projection-26-Image Export-13_c2.tif]

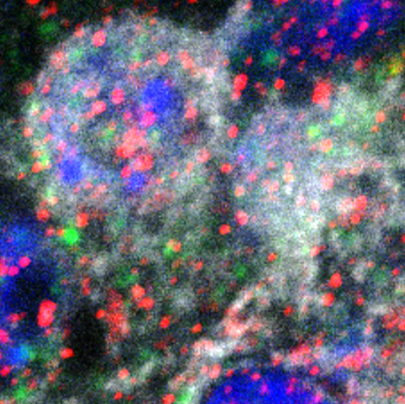

Supplement: Supplementary file 10 — Source data Fig. 7 [file 44319_2026_798_MOESM10_ESM.zip › Figure 7 (V)/7L/7L''.tif]

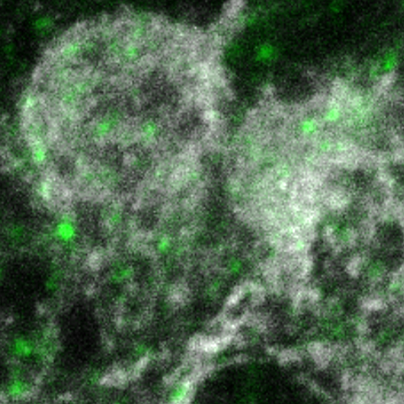

Supplement: Supplementary file 10 — Source data Fig. 7 [file 44319_2026_798_MOESM10_ESM.zip › Figure 7 (V)/7L/7L'.tif]

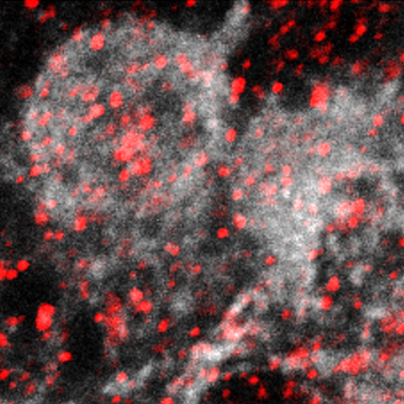

Supplement: Supplementary file 10 — Source data Fig. 7 [file 44319_2026_798_MOESM10_ESM.zip › Figure 7 (V)/7L/7L.tif]

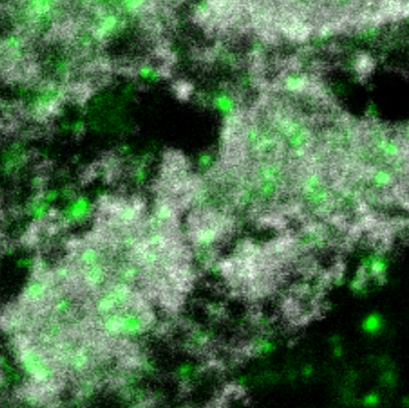

Supplement: Supplementary file 10 — Source data Fig. 7 [file 44319_2026_798_MOESM10_ESM.zip › Figure 7 (V)/7M/7M'.tif]

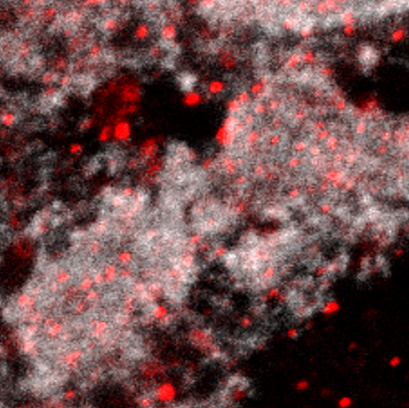

Supplement: Supplementary file 10 — Source data Fig. 7 [file 44319_2026_798_MOESM10_ESM.zip › Figure 7 (V)/7M/7M.tif]

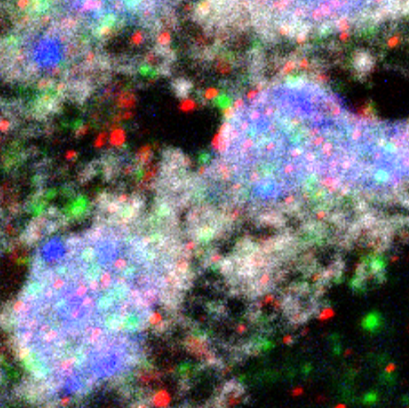

Supplement: Supplementary file 10 — Source data Fig. 7 [file 44319_2026_798_MOESM10_ESM.zip › Figure 7 (V)/7M/M''.tif]
