# Supplementary material for: Activity-dependent development of vocal circuits in the neonatal rodent forebrain
Source: EMBO Rep. 2026 May 19;27(13):3632–64. doi: 10.1038/s44319-026-00798-1 (PMC13354774; doi:10.1038/s44319-026-00798-1)
Supplement: Supplementary file 11 — Expanded View Figures [file 44319_2026_798_MOESM11_ESM.pdf]

Expanded View Figures

Figure EV1. Optimal interval of 4-OHT injection and reactivated neurons in the vmPFC for USV experiment.

(**A**) To determine the optimal interval for detecting activated cells during USV emission, we injected 4-OHT into P8 TRAP2 mice whose striatum had been injected with *AAVrg-hSyn-DIO-EGFP* at P0. The mice were separated from the dam 2, 4, or 6 h after 4-OHT injection, and then perfused 5 days later. (**B**) We found fewer EGFP-positive (+) neurons in the prelimbic cortex (PL) in the 2-h interval group compared with the 4-h and 6-h interval groups. (**C**) Quantification of EGFP+ neurons. (**D**) At P7, all mice received an injection of 4-OHT. Four hours after injection, the mice were separated from their mother to trigger isolation stress-induced USV. Littermates that remained with the dam served as non-separated controls. At P8, both groups underwent a 5-min maternal separation and were perfused 90 min later for the detection of c-Fos expression. Cellular reactivation was quantified by measuring the proportion of c-Fos+ cells within the EGFP+ population. (**E**) Reactivated cells (arrowheads) are identified by co-expression of EGFP (green) and c-Fos (red). (**F**) Quantification of reactivated neurons. The proportion of c-Fos+ cells within the EGFP+ population was significantly higher in the reactivated group compared with the control group. One-way ANOVA is used in (**C**). Independent *t*-test is used in (**F**).

© The Author(s)

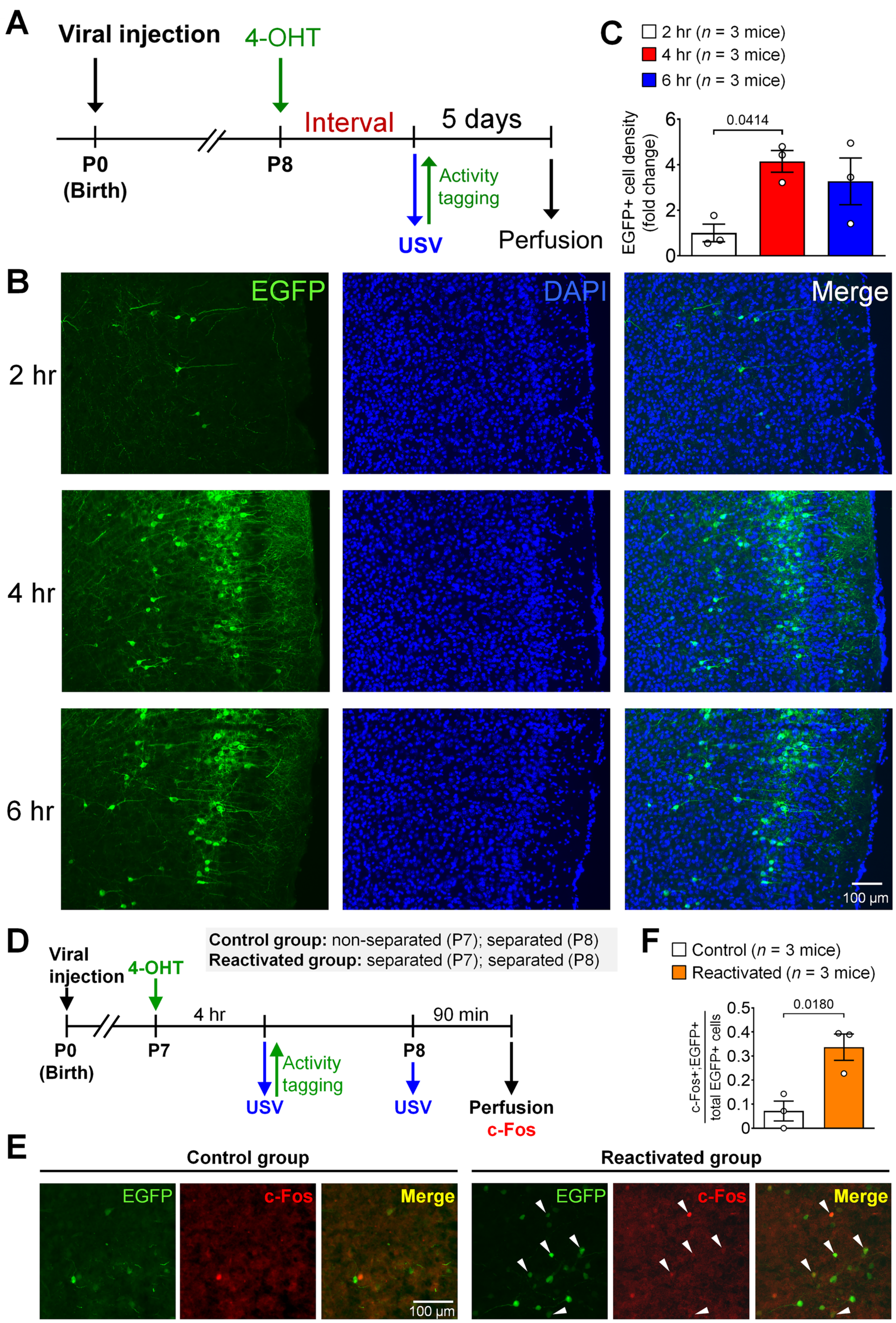
A
Viral injection
4-OHT
Interval
5 days
P0
(Birth)
P8
Activity
tagging
USV
Perfusion
C
2 hr (n = 3 mice)
4 hr (n = 3 mice)
6 hr (n = 3 mice)
EGFP+ cell density
(fold change)
0.0414
B
EGFP
DAPI
Merge
2 hr
4 hr
6 hr
100 μm
D
Control group: non-separated (P7); separated (P8)
Reactivated group: separated (P7); separated (P8)
Viral
injection
4-OHT
4 hr
90 min
P0
(Birth)
P7
P8
Activity
tagging
USV
USV
Perfusion
c-Fos
F
Control (n = 3 mice)
Reactivated (n = 3 mice)
c-Fos+;EGFP+
total EGFP+ cells
0.0180
E
Control group
Reactivated group
EGFP
c-Fos
Merge
100 μm

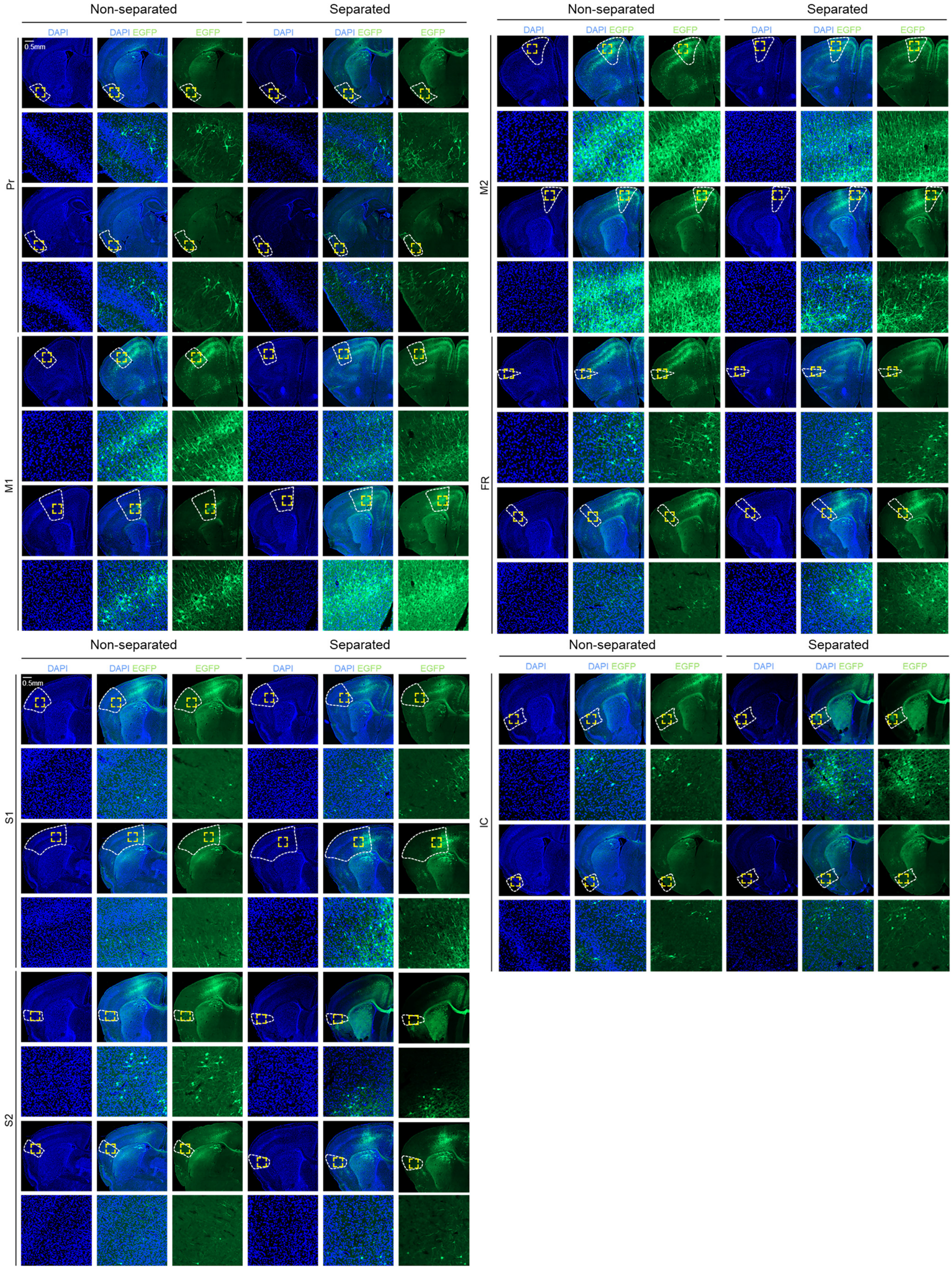

Non-separated
Separated
DAPI
DAPI EGFP
EGFP
0.5mm
Pr
M1
M2
FR
S1
S2
IC

 © The Author(s)

Figure EV2. Localization of vocalization-related corticostriatal ensembles in neonatal mice.

EGFP signals, identified by GFP immunostaining, indicate the activated neurons during USV emission. EGFP-positive neurons were quantified among 13 cortical regions, including prelimbic, infralimbic, medial orbital, cingulate, lateral/ventral orbital, dorsal peduncular (shown in Fig. 1), piriform (Pr), primary motor (M1), secondary motor (M2), frontal area (FR), primary somatosensory (S1), secondary somatosensory (S2), and insular (IC) cortices. This figure provides additional representative images. In a subset of panels, images are derived from the same sections as those shown in Fig. 1C but correspond to distinct, non-overlapping regions of interest.

© The Author(s)

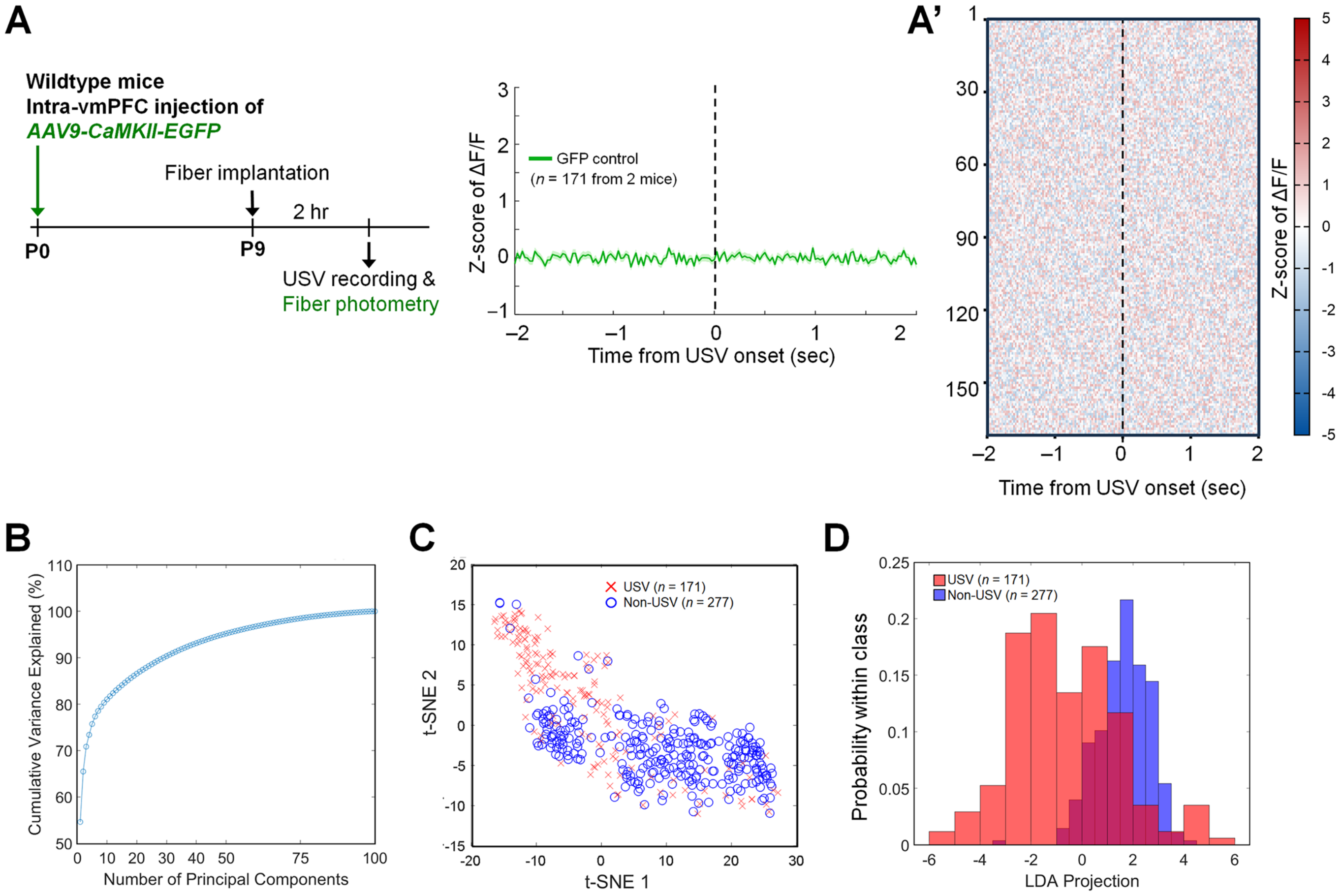

Figure EV3. Dimensionality reduction and discriminative projection of vmPFC trace patterns during USV and non-USV periods.

(**A–A′**) Schematic experimental design and the peri-event GFP signals recorded during fiber photometry. The green line shows the mean value, and the shaded green region indicates the standard error of the mean. The normalized signals of each event are aligned and shown in a heat map. The warmer color represents the stronger signals. (**B**) Cumulative variance explained by the top 100 principal components (PC) derived from USV and non-USV groups. (**C**) The t-SNE projection using PC1-PC10, showing the distribution of trace patterns in an alternative nonlinear projection. (**D**) Histogram of LDA projection scores using PC1-PC10.

 © The Author(s)

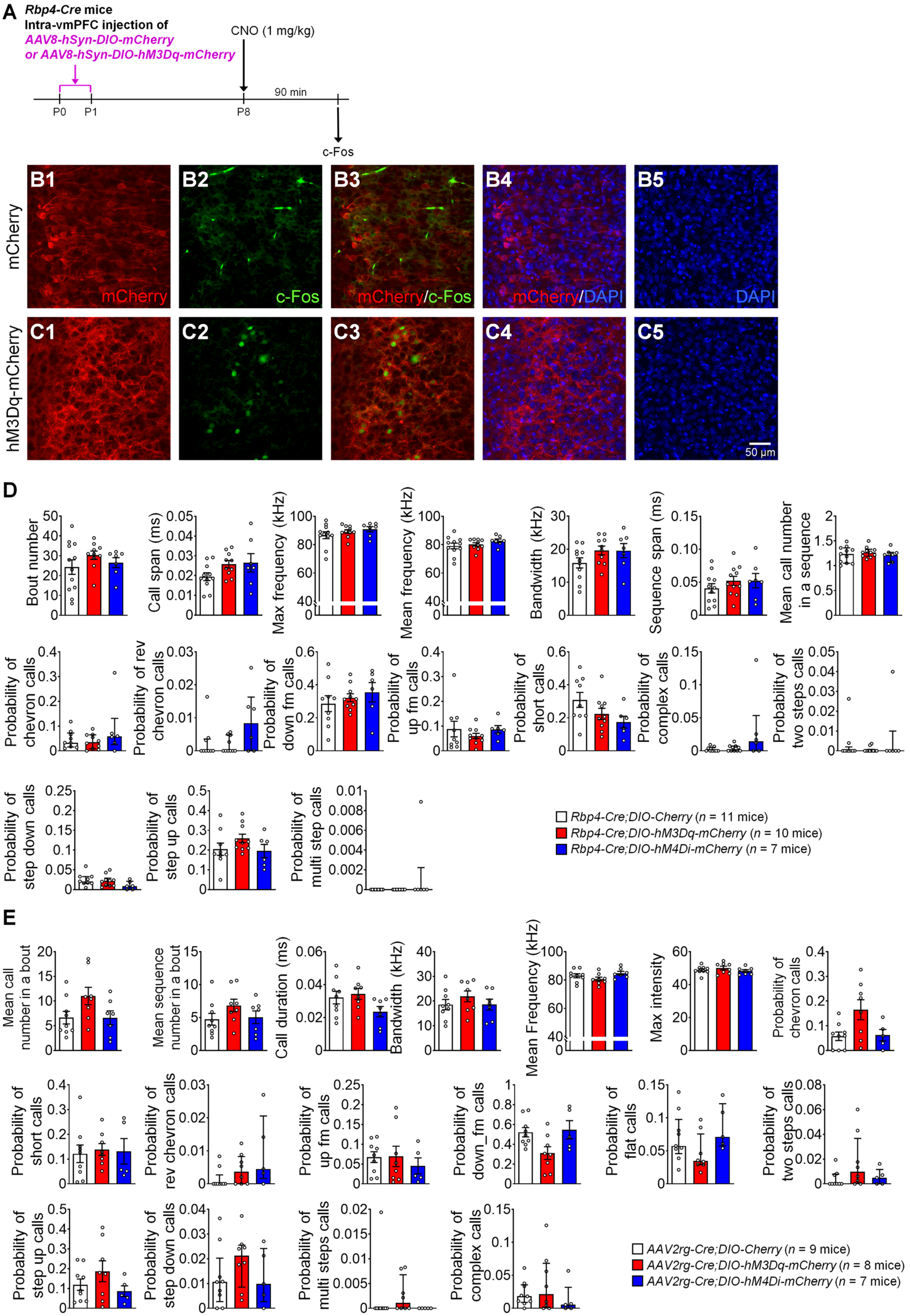
A
Rbp4-Cre mice
Intra-vmPFC injection of
AAV8-hSyn-DIO-mCherry
or AAV8-hSyn-DIO-hM3Dq-mCherry
CNO (1 mg/kg)
90 min
P0
P1
P8
c-Fos
mCherry
hM3Dq-mCherry
B1
B2
B3
B4
B5
C1
C2
C3
C4
C5
mCherry
c-Fos
mCherry/c-Fos
mCherry/DAPI
DAPI
50 µm
D
Bout number
Call span (ms)
Max frequency (kHz)
Mean frequency (kHz)
Bandwidth (kHz)
Sequence span (ms)
Mean call number in a sequence
Probability of chevron calls
Probability of rev chevron calls
Probability of down fm calls
Probability of up fm calls
Probability of short calls
Probability of complex calls
Probability of two steps calls
Probability of step down calls
Probability of step up calls
Probability of multi step calls
Rbp4-Cre;DIO-Cherry (n = 11 mice)
Rbp4-Cre;DIO-hM3Dq-mCherry (n = 10 mice)
Rbp4-Cre;DIO-hM4Di-mCherry (n = 7 mice)
E
Mean call number in a bout
Mean sequence number in a bout
Call duration (ms)
Bandwidth (kHz)
Mean Frequency (kHz)
Max intensity
Probability of chevron calls
Probability of short calls
Probability of rev chevron calls
Probability of up fm calls
Probability of down_fm calls
Probability of flat calls
Probability of two steps calls
Probability of step up calls
Probability of step down calls
Probability of multi steps calls
Probability of complex calls
AAV2rg-Cre;DIO-Cherry (n = 9 mice)
AAV2rg-Cre;DIO-hM3Dq-mCherry (n = 8 mice)
AAV2rg-Cre;DIO-hM4Di-mCherry (n = 7 mice)

Figure EV4. Validation of hM3Dq activation and analysis of USV properties in DREADD-manipulated mice.

(**A**) The schematic experimental design. (**B**, **C**) Increased c-Fos expression was observed in vmPFC neurons expressing hM3Dq-mCherry (C1–C5) compared with neurons expressing the control virus (B1–B5) 90 min after CNO administration. (**D**) USV acoustic and syntactic parameters were not significantly altered in mice with acute vmPFC DREADD manipulation. (**E**) USV acoustic and syntactic parameters were not significantly altered in mice with acute manipulation of corticostriatal projection neurons.

 © The Author(s)

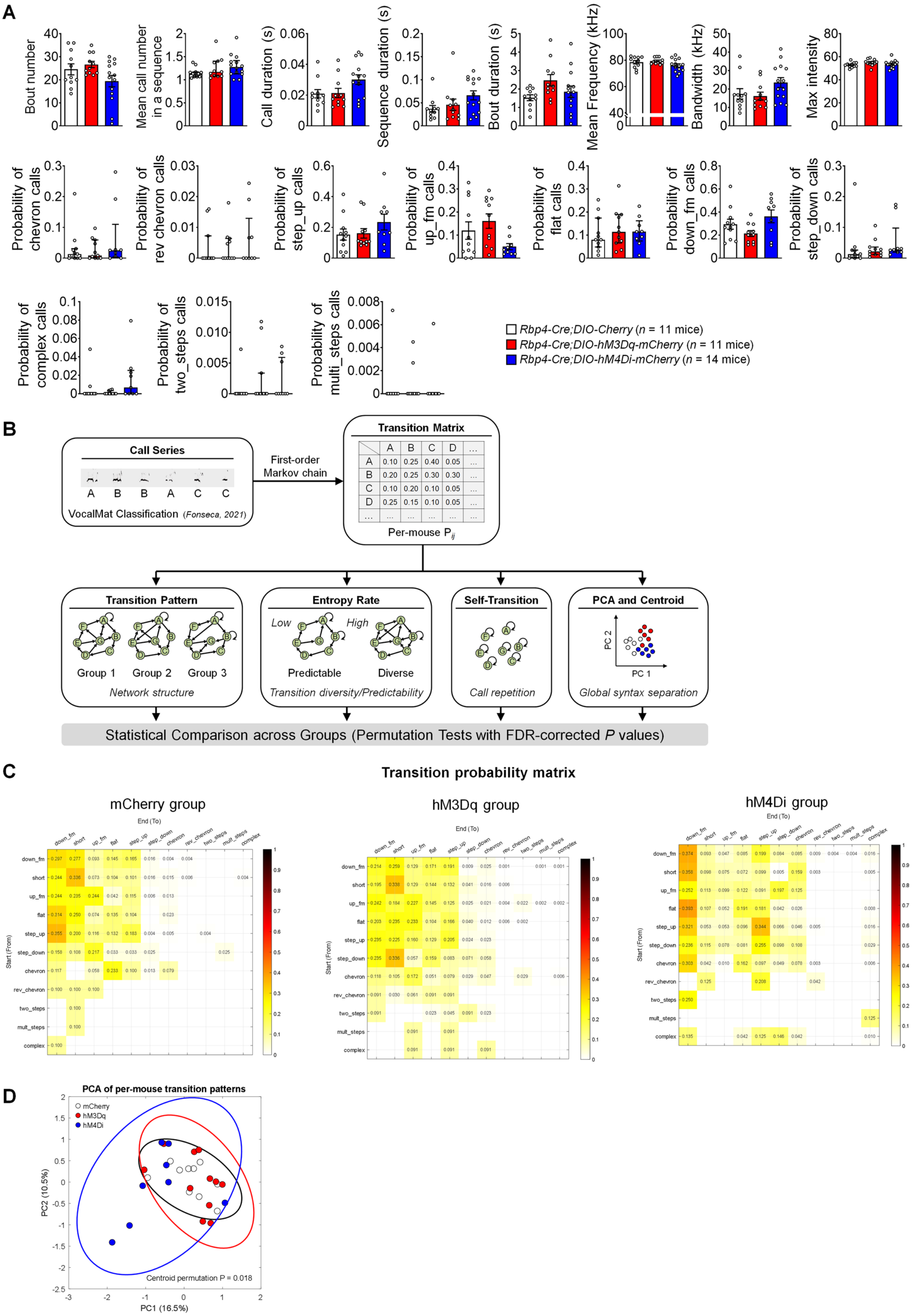
A
Bout number
Mean call number in a sequence
Call duration (s)
Sequence duration (s)
Bout duration (s)
Mean Frequency (kHz)
Bandwidth (kHz)
Max intensity
Probability of chevron calls
Probability of rev chevron calls
Probability of step_up calls
Probability of up_fm calls
Probability of flat calls
Probability of down_fm calls
Probability of step_down calls
Probability of complex calls
Probability of two_steps calls
Probability of multi_steps calls
Rbp4-Cre;DIO-Cherry (n = 11 mice)
Rbp4-Cre;DIO-hM3Dq-mCherry (n = 11 mice)
Rbp4-Cre;DIO-hM4Di-mCherry (n = 14 mice)
B
Call Series
A B B A C C
VocalMat Classification (Fonseca, 2021)
First-order Markov chain
Transition Matrix
Per-mouse Pij
Transition Pattern
Group 1 Group 2 Group 3
Network structure
Entropy Rate
Low High
Predictable Diverse
Transition diversity/Predictability
Self-Transition
Call repetition
PCA and Centroid
PC 2
PC 1
Global syntax separation
Statistical Comparison across Groups (Permutation Tests with FDR-corrected P values)
C
Transition probability matrix
mCherry group
hM3Dq group
hM4Di group
End (To)
Start (From)
D
PCA of per-mouse transition patterns
mCherry
hM3Dq
hM4Di
Centroid permutation P = 0.018
PC1 (16.5%)
PC2 (10.5%)

Figure EV5. Analysis of USV properties and USV syntax in DREADD-manipulated mice.

(**A**) USV acoustic and syntactic parameters were not significantly altered in mice with chronic vmPFC DREADD manipulation. (**B**) Schematic flowchart of Markov chain analysis. (**C**) Transition probability matrix of each group. (**D**) Principal component analysis (PCA) of transition patterns across groups. Group ellipses illustrate clustering in PC space.

 © The Author(s)

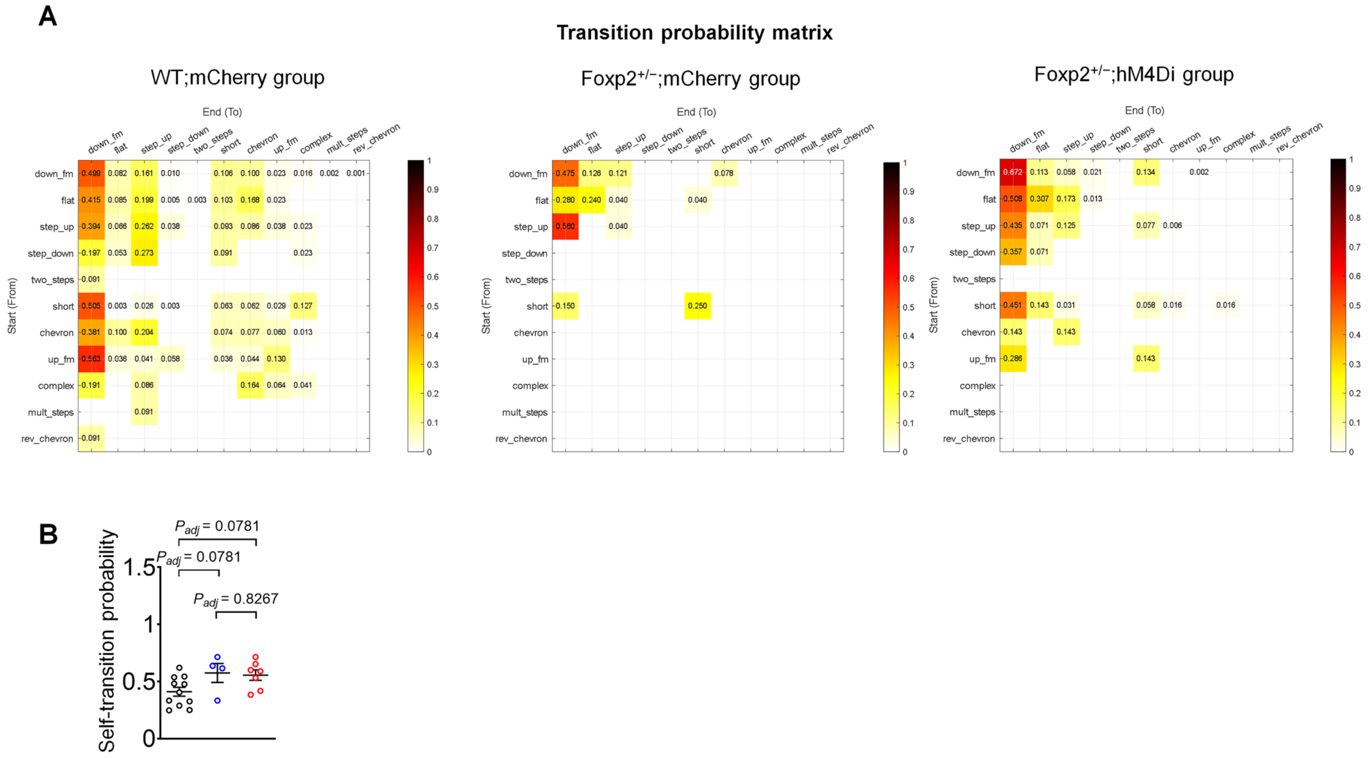

Figure EV6. Analysis of USV properties and USV syntax in DREADD-manipulated mice.

(A) Transition probability matrix of each group. **(B)** Per-mouse permutation test of USV self-transition probability across groups.
