# Supplementary material for: Activity-dependent development of vocal circuits in the neonatal rodent forebrain
Source: EMBO Rep. 2026 May 19;27(13):3632–64. doi: 10.1038/s44319-026-00798-1 (PMC13354774; doi:10.1038/s44319-026-00798-1)
Supplement: Supplementary file 2 — Peer Review File [file 44319_2026_798_MOESM2_ESM.pdf]

EMBO reports

# Activity-dependent development of vocal circuits in the neonatal rodent forebrain

*Shih-Yun Chen2, Hao-Yu Pang, Pao-Wen Fan, Guan-Ying Wu, Wan-Ting Lin, Fu-Chin Liu, and Hsiao-Ying Kuo*

*Corresponding author(s): Hsiao-Ying Kuo (kuohy@nycu.edu.tw)*

**Review Timeline:**

| | |
|---|---|
| Submission Date: | 21st Jun 25 |
| Editorial Decision: | 12th Sep 25 |
| Revision Received: | 14th Mar 26 |
| Editorial Decision: | 14th Apr 26 |
| Revision Received: | 20th Apr 26 |
| Accepted: | 28th Apr 26 |

*Editor: Esther Schnapp*

## Transaction Report:

(Note: With the exception of the correction of typographical or spelling errors that could be a source of ambiguity, letters and reports are not edited. Depending on transfer agreements, referee reports obtained elsewhere may or may not be included in this compilation. Referee reports are anonymous unless the Referee chooses to sign their reports.)

**1st Editorial Decision** **12th Sep 2025**

Dear Dr. Kuo,

Thank you for your patience while your manuscript was peer-reviewed at EMBO reports. I am sorry for the delay in getting back to you, referee 2 never sent a report and I thus had to find a 4th referee, which delayed the entire process. All 3 reports have come in now and are pasted below.

As you will see, the referees acknowledge that the findings are potentially interesting. However, they also have several concerns, and the most important one is that indirect effects of FoxP2 mutations could cause the changes in iso USV rates. This concern must be addressed. All other points raised by the referees are also relevant and should be addressed somehow. Please let me know if you have any comments regarding the referee reports, and we can discuss the exact revision requirements further, also in a video chat, if you like.

I would thus like to invite you to revise your manuscript with the understanding that the referee concerns must be fully addressed and their suggestions taken on board. Please address all referee concerns in a complete point-by-point response. Acceptance of the manuscript will depend on a positive outcome of a second round of review. It is EMBO reports policy to allow a single round of major revision only and acceptance or rejection of the manuscript will therefore depend on the completeness of your responses included in the next, final version of the manuscript.

We realize that it is difficult to revise to a specific deadline. In the interest of protecting the conceptual advance provided by the work, we recommend a revision within 3 months (13th Dec 2025). Please discuss the revision progress ahead of this time with the editor if you require more time to complete the revisions.

IMPORTANT NOTE: we perform an initial quality control of all revised manuscripts before re-review. Your manuscript will FAIL this control and the handling will be DELAYED if the following APPLIES:
1) A data availability section providing access to data deposited in public databases is missing. If you have not deposited any data, please add a sentence to the data availability section that explains that.
2) Your manuscript contains statistics and error bars based on n=2. Please use scatter blots in these cases. No statistics should be calculated if n=2.

When submitting your revised manuscript, please carefully review the instructions that follow below. Failure to include requested items will delay the evaluation of your revision.

1) a .docx formatted version of the manuscript text (including legends for main figures, EV figures and tables). Please make sure that the changes are highlighted to be clearly visible.

2) individual production quality figure files as .eps, .tif, .jpg (one file per figure). See https://wol-prod-cdn.literatumonline.com/pb-assets/embo-site/EMBOPress_Figure_Guidelines_061115-1561436025777.pdf for more info on how to prepare your figures.

3) We replaced Supplementary Information with Expanded View (EV) Figures and Tables that are collapsible/expandable online. A maximum of 5 EV Figures can be typeset. EV Figures should be cited as 'Figure EV1, Figure EV2" etc... in the text and their respective legends should be included in the main text after the legends of regular figures.

- For the figures that you do NOT wish to display as Expanded View figures, they should be bundled together with their legends in a single PDF file called *Appendix*, which should start with a short Table of Content. Appendix figures should be referred to in the main text as: "Appendix Figure S1, Appendix Figure S2" etc. See detailed instructions regarding expanded view here: <https://www.embopress.org/page/journal/14693178/authorguide#expandedview>

- Additional Tables/Datasets should be labeled and referred to as Table EV1, Dataset EV1, etc. Legends have to be provided in a separate tab in case of .xls files. Alternatively, the legend can be supplied as a separate text file (README) and zipped together with the Table/Dataset file.

4) a .docx formatted letter INCLUDING the reviewers' reports and your detailed point-by-point responses to their comments. As part of the EMBO Press transparent editorial process, the point-by-point response is part of the Review Process File (RPF), which will be published alongside your paper.

5) a complete author checklist, which you can download from our author guidelines <https://www.embopress.org/page/journal/14693178/authorguide>. Please insert information in the checklist that is also reflected in the manuscript. The completed author checklist will also be part of the RPF.

6) Please note that all corresponding authors are required to supply an ORCID ID for their name upon submission of a revised manuscript (<https://orcid.org/>). Please find instructions on how to link your ORCID ID to your account in our manuscript tracking system in our Author guidelines

<https://www.embopress.org/page/journal/14693178/authorguide#authorshipguidelines>

7) Before submitting your revision, primary datasets produced in this study need to be deposited in an appropriate public database (see https://www.embopress.org/page/journal/14693178/authorguide#datadeposition). Please remember to provide a reviewer password if the datasets are not yet public. The accession numbers and database should be listed in a formal "Data Availability" section placed after Materials & Method (see also https://www.embopress.org/page/journal/14693178/authorguide#datadeposition). Please note that the Data Availability Section is restricted to new primary data that are part of this study. * Note - All links should resolve to a page where the data can be accessed. *
If your study has not produced novel datasets, please mention this fact in the Data Availability Section.

8) At EMBO Press we ask authors to provide source data for the main manuscript figures. You will receive a separate email with instructions for providing source data with your revised manuscript, including how to upload and organize the files.

9) Our journal also encourages inclusion of *data citations in the reference list* to directly cite datasets that were re-used and obtained from public databases. Data citations in the article text are distinct from normal bibliographical citations and should directly link to the database records from which the data can be accessed. In the main text, data citations are formatted as follows: "Data ref: Smith et al, 2001" or "Data ref: NCBI Sequence Read Archive PRJNA342805, 2017". In the Reference list, data citations must be labeled with "[DATASET]". A data reference must provide the database name, accession number/identifiers and a resolvable link to the landing page from which the data can be accessed at the end of the reference. Further instructions are available at https://www.embopress.org/page/journal/14693178/authorguide#referencesformat

10) Regarding data quantification (see Figure Legends: https://www.embopress.org/page/journal/14693178/authorguide#figureformat)

The following points must be specified in each figure legend:

- the name of the statistical test used to generate error bars and P values,

- the number (n) of independent experiments (please specify technical or biological replicates) underlying each data point,

- the nature of the bars and error bars (s.d., s.e.m.),

- If the data are obtained from n Program fragment delivered error ``Can't locate object method "less" via package "than" (perhaps you forgot to load "than"?) at //ejpvfs23/sites23b/embor_www/letters/embor_decision_revise_and_review.txt line 56.' 2, use scatter blots showing the individual data points.

Discussion of statistical methodology can be reported in the materials and methods section, but figure legends should contain a basic description of n, P and the test applied.

- Please also include scale bars in all microscopy images.

11) The journal requires a statement specifying whether or not authors have competing interests (defined as all potential or actual interests that could be perceived to influence the presentation or interpretation of an article). In case of competing interests, this must be specified in your disclosure statement. Further information: https://www.embopress.org/competing-interests

12) All Materials and Methods need to be described in the main text using our 'Structured Methods' format, which is required for all research articles. According to this format, the Methods section includes a separate Reagents and Tools Table file (listing key reagents, experimental models, software and relevant equipment and including their sources and relevant identifiers) and a Methods and Protocols section describing the methods using a step-by-step protocol format. The aim is to facilitate adoption of the methodologies across labs. More information on how to adhere to this format as well as a downloadable template (.docx) for the Reagents and Tools Table can be found in our author guidelines: https://www.embopress.org/page/journal/14693178/authorguide#structuredmethods.

An example of a Method paper with Structured Methods can be found here: https://www.embopress.org/doi/full/10.1038/s44320-024-00037-6#sec-4

We would also welcome the submission of cover suggestions, or motifs to be used by our Graphics Illustrator in designing a cover.

As part of the EMBO publication's Transparent Editorial Process, EMBO reports publishes online a Review Process File (RPF) to accompany accepted manuscripts. This File will be published in conjunction with your paper and will include the referee reports, your point-by-point response and all pertinent correspondence relating to the manuscript.

You are able to opt out of this by letting the editorial office know (emboreports@embo.org). If you do opt out, the Review Process File link will point to the following statement: "No Review Process File is available with this article, as the authors have chosen not to make the review process public in this case."

I look forward to seeing a revised form of your manuscript when it is ready.

Best regards,
Esther

Esther Schnapp, PhD
Senior Editor
EMBO reports

Referee #1:

This study was designed to identify neuronal circuits that are involved in ultrasonic vocalization in mouse pups. The experimental design incorporates some cutting-edge techniques. The manuscript would be improved by incorporating the following specific points:

1) The scientific justification for using FoxP2 mutant mice is not clear. What is measured in this study is social communication in mouse pups, whereas FoxP2 is implicated in language development in humans. Neonatal social communication is distinct from human language (PMID: 28204487). Or is there evidence that FoxP2 mutant carriers are impaired in neonatal non-language vocalization?
2) It seems unclear to this reviewer that "no USV induction" is genuinely no USV induction. It could be that pups emitted USV before recording started.
3) The use of Fos-Cre is a major weakness of this study. Fos and other immediate early genes are not genuine markers of cell activity; there are activated cells without Fos (i.e., false negative) and there are inactive cells that are Fos-positive (i.e., false positive). There is no good correlation between $Ca^{2+}$ influx and IEG expression.
4) Student t-tests were used to analyze data in Figure 1D, 2D-F, 6EFK, 7IJ. ANOVAs were used for Figs. 3, 4, 5EFG, and 7F. Please present a summary Excel that shows that the assumption of homogeneity of variance was not violated to justify these parametric tests.
5) Only qualitative analyses are applied to the data in Figs. 5 and 6. The number of cells, as well as the intensities of VgGlut1 and FoxP2, should be quantitatively analyzed.
6) In Figure 7I and 7J, WT control group should be set as 1.0.

Referee #3:

The manuscript shows very nice work on the neural mechanisms involved in neonatal mouse vocalizations induced by maternal separation and on cortico-striatal behavioral effects and plasticity. The work is impressive due to several reasons. It confirms the relevance of prefrontal cortical activity for USV generation. It involves implantation and fiberphotometry in neonatal pups, which is not done a lot in the field. And, the authors show activity dependent wiring in the striatum and an upregulation of FoxP2 following sustained prefrontal cortex activation. They even show that this manipulation can rescue vocal deficits of FoxP2 mutant mice, which has strong translational potential.
I suggest that this article will be published in EMBO Reports, however, I have one major and multiple minor issues that the authors should address before publication.

Major: The authors claim that cortico-striatal projections are responsible for USV generation (Figures 1-4). However, in none of these experiments the authors specifically investigated or manipulated cortico-striatal projections. In Figure 1 fos activated cells were counted. In Figure 2 the authors used CamKII promoter AAV to image prefrontal cortex activity and in Figures 3 and 4 the authors use a layer V specific mouse line. While layer V contains cortico-striatal projection neurons the observed effects could also be mediated by other projections. Although the activity dependent wiring and upregulation shown in Figures 5-7 suggests that the effect is indeed driven by cortico-striatal projection neurons the effect on vocalizations (Figures 1-4) cannot be proven by this. I strongly suggest to use a retrograde AAV approach (e.g. AAV-retro-Cre in striatum and AAV-DIO-DREADD in PL in WT mice).

Related and minor comments:
In Figure 1 the authors nicely analyze different areas and find that PL is mostly active in vocalizing mice. However, they did not provide a quantitative analysis for the layers but just show an example image (Fig1E). This has to be analyzed more deeply.

Figure 2G-L shows a sophisticated analysis using various dimensional reduction techniques, which at first glance does not add too much to the conventional analysis performed in Fig2A-F'. I then, however, understood that the authors tried to predict USVs solely by the neuronal traces. Fig2L shows a reconstructed fluorescence signal that seems to capture the kinetics of the real data shown in Fig2B. However, despite the authors state "a discriminative power in predicting USV occurrence from neural trace dynamics" they do not provide a panel showing the accuracy of USV prediction by their tools. If I'm not mistaken the authors should train a support vector machine with the reduced components to get a proper classifier / predictor. If they can also show that their LDA method works fine it's ok but they then have to show the decoding accuracy (incl. false positives and negatives) in a test data set, which is distinguished from the training data set.

In Figure 3A the authors state "Corticostriatal neuron-specific Cre". If I'm not mistaken Rbp4 is a layer V marker contains not exclusively striatal projection cells. Thus, with the methods in
Figure 3 the authors manipulate corticostriatal neurons but potentially also other projection neurons. The authors should thus rewrite this or include an experiment in which they inject retrograde Cre-dependent chemogenetic AAVs in the striatum.
The text corresponding to Figure 4 is headed by this subtitle: "Chronic activation of layer V vmPFC neurons during postnatal development increases corticostriatal innervation". However, neither in the Figure or in the text I find experiments in which the corticostriatal innvervation was really investigated. The experiments in Figure 4 show that chronic chemogenetic manipulation leads to increase of USVs numbers and a decrease of USV durations and frequency jumps (Figure 4B), which has nothing to do with corticostriatal innervation. The authors again should either rewrite or provide respective experiments.

In Figure 5 the authors indeed investigate corticostriatal projections. The result is very interesting but the authors should be careful to state that the VGLUT1+ puncta are corticostriatal projection fibers. As the authors cannot be 100% sure that these fibers may just pass-by the should avoid the term innervation in the respective subtitle. In the text below the authors even state "Since chronic activation of vmPFC corticostriatal neurons increased synaptic connectivity [...]". Again, counting projection fibers is not sufficient to make a statement about synaptic connectivity and experiments have to be provided if the claim wants to be held up (additional pre-postsynaptic marker stainings, electron microscopy or ex-vivo patching).

Referee #4:

This study seeks to understand the role of the ventromedial prefrontal cortex in regulating the production of isolation USVs in mouse pups, as well as in regulating synapse formation and gene expression in the striatum. The authors use the TRAP2 strategy to label striatum-projecting cortical neurons that are active in association with isolation USV production. Next, they perform fiber photometry to test whether vmPFC-striatal neurons increase their activity during times of isolation USV production. Then they test the effects of chemogenetic manipulations of activity (Rbp4-expressing vmPFC neurons) on isolation USV production. They test the effects of chronic chemogenetic manipulations on isolation USV production and VGlut1 and FoxP2 expression in striatum. Finally, they test the effects of chronic chemogenetic activation of vmPFC-striatal neurons on isolation USV production in FoxP2 heterozygote mutant mice. Although their experiments reveal interesting effects on isolation USV production, including in FoxP2 mutants, the specificity of these effects to vocal behavior is not established. Overall, many of the conclusions are incompletely supported by the data, some analyses are flawed and/or incomplete, and some experiments are missing important controls.

Major
Introduction
(1) In the Introduction, the authors' description of the two vocalization pathways differs in important details from the description offered by Jurgens, 2009. In Jurgens's view, the first pathway includes ACC to PAG to premotor neuron pools. The second pathway includes motor cortex (as well as inputs to motor cortex), which bypasses the PAG and instead projects directly to premotor neuron pools. It is possible that the authors disagree with some details of this model of vocal motor circuits. Their language, however, suggests that they are summarizing Jurgens's view of these pathways, despite the mismatch between their descriptions and those of Jurgens. This section should be corrected/clarified.
(2) In the Introduction, the authors selectively present past work that is in line with their hypothesis (that FoxP2 regulates vocal communication, even in a non-vocal-learner such as the mouse). I don't take issue with the evidence that FoxP2 regulates vocal communication in vocal learners (humans, songbirds). However, the evidence that FoxP2 regulates mouse USV production is complicated. First, FoxP2 mutant mice typically have severe developmental delays. As such, it is not clear whether effects on iso USV production stem directly from changes to circuits that regulate vocalization vs. indirectly from developmental delays. Related to this idea is recent work that found that rates of iso USVs in mouse pups are well related to the intensity of non-vocal movements (Pranic et al., 2021). How can we rule out the possibility that changes in iso USV rates in these FoxP2 models stem indirectly from effects of the FoxP2 mutations on pup non-vocal movement and/or behavioral arousal? Second, the authors do not cite work by Urbanus et al., 2020, who showed that region-specific FoxP2 deletions do not recapitulate the effects of global FoxP2 deletion on iso USV production. This study certainly calls into question the idea that FoxP2 expression in corticostriatal circuits regulates mouse iso USV production. At the very least, these studies/caveats need to be mentioned in the manuscript.

TRAP experiments
(3) The conclusion that the authors could "tag USV-recruited cell ensembles with reporter gene without significant confounding populations of non-USV cells" is not substantiated. The authors do not include experiments to establish what proportion of the labeled neurons in different regions are related to USVs vs. not USVs, which would be needed to support this statement. It is important to note that neurons labeled in their experimental pups could be activated in relation to any number of things that occur in the iso session and/or after the iso session (USV production, decreased body temperature, increased behavioral arousal, stress of being in a plastic cup, changes in maternal behavior after pup is returned to the nest, etc.). The language here should be modified.
(4) Related to the above point, why was 4-OHT administered 4 hours before the isolation session? This seems different relative to the optimal time course established in the original TRAP paper. If the authors claim that this timing gave them optimal GFP labeling, they need to show quantification of labeling obtained with different timings of 4-OHT delivery relative to the behavior session. The inclusion of such data would strengthen their claim that their timing of 4-OHT delivery gave optimal neuronal labeling.
(5) The authors show that the number of GFP-labeled vmPFC neurons was higher in experimental pups than in controls, but this finding does not fully support their conclusion that the labeled neurons are USV-related. As mentioned above, many things differ between the experimental and control groups. The authors should include an analysis within the experimental group, to test whether the number of labeled vmPFC neurons is positively correlated to the number of iso USVs produced across different experimental pups. Such an analysis would substantially strengthen the authors' claim that the labeled neurons are related to USV production.
(6) The authors should validate their findings from the TRAP labeling experiment with a Fos staining? Is Fos expression in vmPFC-striatal neurons positively correlated with iso USV rates in experimental pups? The time window of TRAP2 labeling is really long (as noted by the authors, many hours) compared to the time resolution of Fos immunohistochemistry.
(7) The included cortical regions are large. Which and how many planes of section were considered and/or averaged in these quantifications (Fig. 1D)? The inclusion of these details is critical to evaluate the data.

Fiber photometry
(8) Previous work has found that rates of pup iso USVs are correlated with the intensity of body movements (Pranic et al., 2021). Were videos collected of the pup's behavior during fiber photometry sessions? Can you rule out the possibility that vmPFC signals are related to body movements/behavioral arousal?
(9) The authors should include GFP controls, to rule out the possibility that some signals resulted from movement artifacts. The shuffled control group does not allow the authors to test this point or the previous point.
(10) The authors should include representative traces of raw data (example periods of photometry signal along with either a spectrogram of USVs or a raster plot showing the timing of USVs). Without seeing any raw data, it is difficult to understand to what degree vmPFC-striatal activity was selectively related to USV production vs. behavioral events that occur outside of USV times.
(11) I'm not following the purpose of the PCA/t-Sne analyses of photometry signals collected around USV times vs. non-USV times. The non-USV times would be expected to contain a random smattering of transients (presumably, there are some transients outside of USV times, see previous point) and non-transients. One would expect USV times vs. non-USV times to look different, but why is that interesting?
(12) In Fig. 2B, the summary data should be the average of the averages for the n = 4 mice. It appears from the description that the authors have instead averaged across all USV onsets (n = 170 evens). This is a flawed way to perform the analysis, as the average shown could be the result of one mouse with a great relationship between USVs and vmPFC activity and 3 mice with a poor/weak relationship.
(13) It is somewhat unexpected that the averaged USV-aligned photometry signal decays in Fig. 2B and 2B' begins to decay so rapidly. Pups often produce bouts of iso USVs. It would strengthen this results section to separately analyze photometry signals aligned to onsets of single iso USVs (i.e., iso USVs that are preceded by and followed by silence) vs. bouts of USVs (multiple USVs back-to-back) to understand whether the temporal dynamics of the photometry signals differ according to bout length.

Acute chemogenetic manipulations of activity
(14) In Figure 3C, why does the viral labeling in the mCherry group look so sparse compared to the labeling in the hM3Dq group? It is also concerning that nearly every mCherry-positive neuron expresses Fos in the control group. This shouldn't be the case.
(15) Were videos collected? Can the authors rule out the possibility that changes in iso USV rates were secondary to changes in movement and/or behavioral arousal?
(16) I understand that optogenetics is more challenging than chemogenetics to implement in pups. However, the conclusion that vmPFC activation promotes USVs in a relatively direct fashion would be strengthened if the authors could show that vmPFC opto activation elicits USVs (during/following laser-on periods).

Chronic chemogenetic activation/inhibition vs. corticostriatal innervation, VGlut1 and FoxP2 expression
(17) In Figure 4B, left-most panel, is the number of USVs in the hM4Di group significantly different from controls, as well as from the hM3Dq group? If not, rather than saying "hM4Di inhibition decreased USV emissions compared to the hM3Dq group", the authors should say that hM4Di inhibition did not change USV emissions relative to controls.
(18) The results subheading "Chronic activation of layer V vmPFC neurons during postnatal development increases corticostriatal innervation" looks like a mistake, given that this results section only describes effects of chronic chemogenetic

manipulations of USV production.
(19) Were videos collected? Are the non-vocal behaviors of pups that have experienced chronic activation or chronic inhibition of vmPFC different from controls?
(20) The sample size is low for the hM4Di group relative to the others. The number of USVs in the hM4Di group looks like it tends to be lower than controls, and I wonder if the experiment is underpowered.
(21) It seems problematic to me that the analyses in Figure 5 were performed at the level of individual cells, rather than by animal. How can we know whether the changes in VGlut1 expression are robust across animals in the hM3Dq experimental group?
(22) In Figure 5G, the counts of neurons are too small, and the analysis seems underpowered. Given the small numbers of neurons and the trend that mirrors the findings in Fig. 5E/F (hM3Dq > controls and hM4Di), I don't think the authors' claim that the changes in VGlut expression are selective for striatal neurons innervated by mCherry axons is well supported.
(23) The results seem to state that chronic inhibition of vmPFC neurons causes a downregulation of FoxP2 relative to controls. However, Fig. 6E seems to show that hM4Di does not differ from controls. The authors should adjust the language to ensure that the conclusions correspond to the data.

Chronic chemogenetic activation in FoxP2 hets
(24) The authors state that they manipulate vmPFC-striatal neurons, but the strategy used in this experiment (Rbp4-Cre) is not specific to that cell type.
(25) Were videos collected? In addition to promoting USV production, does chronic chemogenetic activation of vmPFC neurons alter the non-vocal behaviors of FoxP2 mutant mice?
(26) Does the degree of iso USV rescue correlate with the degree of FoxP2 expression level rescue across animals in the hM3Dq group? If so, this would strengthen the claim that FoxP2 expression might be related in some way to the rescue of iso USV rates.

Minor
(1) The sentence in the abstract that includes "chronic activation of vmPFC-striatal neurons not only increased FoxP2...but also VGlut1-labeled synapses" would be clearer if the authors modified the language to clarify where (in what brain region) these changes were observed.
(2) In the first sentence of the Intro, I'm not sure what is meant by "vocal plurality".
(3) Based on the findings, I find the title to be misleading, as I don't think the authors have demonstrated that vmPFC-striatal neurons are part of a vocalization circuit
(4) In the TRAP experiments, were the subjects males, females, or both?
(5) Were the measurements of FoxP2 and VGlut1 expression conducted in the same hM3Dq mice, or in two different sets?
(6) In the Discussion, the authors say "Due to the low level of USV events in Rbp4-Cre mice, we did not find a significant reduction in USV emissions in pups with chronic inhibition." I don't find this reasoning to be sound, as the USV rates look high enough to be able to detect a significant decrease with a sufficiently powered dataset.
(7) In the Discussion, the authors cite Zheng et al., 2022 and state that vmPFC projects to the vocal motor organ in Alston's singing mice. It is important to clarify that that study used polysynaptic tracing, and that the vmPFC does not project directly to the larynx.

***Referee #1:***

***This study was designed to identify neuronal circuits that are involved in ultrasonic vocalization in mouse pups. The experimental design incorporates some cutting-edge techniques. The manuscript would be improved by incorporating the following specific points:***

***1) The scientific justification for using FoxP2 mutant mice is not clear. What is measured in this study is social communication in mouse pups, whereas FoxP2 is implicated in language development in humans. Neonatal social communication is distinct from human language (PMID: 28204487). Or is there evidence that FoxP2 mutant carriers are impaired in neonatal non-language vocalization?***

**Reply:** We thank the reviewer for raising this important concern regarding the scientific rationale for using *Foxp2* mutant mice. We agree that neonatal social communication in mice is not equivalent to human language. Foxp2 is widely recognized as a key regulator of vocal motor control and sensorimotor integration, rather than solely a language-specific gene. However, multiple studies have demonstrated that *Foxp2* mutations in mice impair non-language vocalizations, including isolation-induced ultrasonic vocalizations (USV). Specifically, the event number (call rate) and duration of isolation-induced ultrasonic vocalization (USV) are reduced in *Foxp2*$^{R552H}$ heterozygote and homozygote (Shu et al., 2005; Fujita et al., 2008; Groszer et al., 2008; Gaub et al., 2010), and *Drd1a-Cre;Foxp2*$^{fl/fl}$ mice (Kuo et al., 2023). Additionally, other USV features such as the number of elements, frequency jump, and peak sound pressure are also impaired in Foxp2 mutant mice (Gaub et al., 2010; Chen et al., 2016; Kuo et al., 2023). Although neonatal social communication in mice differs from human language, Foxp2 mutant neonates do carry impaired social communication. We have revised the Introduction and added "*Foxp2 mutations in mice impair non-language vocalizations, including isolation-induced ultrasonic vocalizations (USV). Specifically, the call rate and duration of isolation-induced USV are reduced in Foxp2R552H heterozygote and homozygote (Shu et al., 2005; Fujita et al., 2008; Groszer et al., 2008; Gaub et al., 2010), and Foxp2 conditional knockout mice (Kuo et al., 2023). Additionally, other USV features, such as the number of elements, frequency jump, and peak sound pressure, are also impaired in Foxp2 mutant mice (Gaub et al., 2010; Chen et al., 2016; Kuo et al., 2023). However, the mechanistic relationship between Foxp2 and mouse USV remains debated, as alterations in pup USV in Foxp2 mutants may also reflect developmental or motor effects rather than direct changes in vocal circuits (Gaub et al., 2010; Pranic et al., 2022). Consistently, a previous study revealed that region-specific Foxp2 knockout did not cause vocal impairment in call number or temporal features (Urbanus et al., 2020). Interestingly, Foxp2 has been shown to regulate synaptic remodeling through spinogenesis and neurite outgrowth (Schulz et al., 2010; Chen et al., 2016; Kuo et al., 2023), both are known to be activity-dependent. These observations suggest that neuronal activity may regulate Foxp2 expression, which in turn controls synaptic development and remodeling in vocal circuits.*" in page 4.

***2) It seems unclear to this reviewer that "no USV induction" is genuinely no USV induction. It could be that pups emitted USV before recording started.***

**Reply:** We thank the reviewer for this critical suggestion. We agree that the term "no USV induction" may have been ambiguous and could be misinterpreted as a recording-related issue. Our original intention was to indicate that the pups were not separated from their mom and therefore did not undergo isolation-induced vocalization. To avoid confusion, we have revised the terminology throughout the manuscript and now refer to these conditions as the "non-separated" and "separated" groups.

***3) The use of Fos-Cre is a major weakness of this study. Fos and other immediate early genes are not genuine markers of cell activity; there are activated cells without Fos (i.e., false negative) and there are inactive cells that are Fos-positive (i.e., false positive). There is no good correlation between Ca2+ influx and IEG expression.***

**Reply:** We thank the reviewer for raising this important concern regarding the use of Fos-based activity labeling. We agree that immediate early genes such as c-Fos are not perfect indicators of

neuronal activity, as some active neurons may not express Fos (false negatives), and Fos expression can occasionally occur in the absence of strong neuronal firing or unrelated to behaviors we studied (false positives). We have now clarified this limitation in the Discussion *"One limitation of the present study is that c-Fos is not a perfect indicator of neuronal activity, as some active neurons may not express Fos (false negatives), and Fos expression can occasionally occur in the absence of strong neuronal firing or be unrelated to the behaviors we studied (false positives). The TRAP2 experiment should therefore be interpreted as identifying candidate neuronal populations engaged during USV production rather than definitively labeling all neurons involved in vocalization."* on Page 24.

In the present study, the TRAP2 strategy was not intended to definitively identify all neurons involved in vocalization, but rather to identify candidate brain regions preferentially recruited during USV production. Therefore, the Fos-dependent tagging experiment should be interpreted as a screening approach that highlights regions enriched for activity during vocalization, rather than as definitive evidence that all labeled neurons are causally involved in vocal behavior. Importantly, we performed two additional experiments to strengthen the interpretation of the TRAP2 results. First, we examined whether retrogradely labeled vmPFC-striatal neurons tagged during the initial USV session were reactivated during a subsequent USV session. We found that a substantial fraction of EGFP-labeled neurons in the vmPFC expressed c-Fos upon re-exposure to the USV paradigm, indicating that neurons tagged during the first USV session were reactivated during the second session. This result indicates that neurons labeled by the TRAP2 strategy constitute a population preferentially reactivated during vocal behavior. Second, we directly examined the relationship between USV production and vmPFC neuronal activation using conventional c-Fos immunostaining in wildtype pups. We found that the density of c-Fos-positive neurons in the vmPFC positively correlated with the USV call number (New Fig. 1I). This analysis provides independent evidence that vmPFC activity is associated with USV production at the behavioral level. Finally, activity-dependent induction of immediate early genes such as c-Fos has been extensively characterized as a transcriptional response linking neuronal activity to downstream cellular changes. Membrane depolarization and calcium influx activate CREB signaling, which in turn drives c-Fos transcription as an early step in activity-dependent gene regulation (Sheng and Greenberg, 1990; PMID: 2157471). These studies support the use of Fos-based strategies as a molecular entry point for identifying neurons engaged during defined behavioral epochs. Together, although Fos-based labeling cannot capture all active neurons, the convergent evidence from TRAP2 labeling, ensemble reactivation, and behavioral correlation supports the conclusion that vmPFC neurons are recruited during isolation-induced USV production.

***4) Student t-tests were used to analyze data in Figure 1D, 2D-F, 6EFK, 7IJ. ANOVAs were used for Figs. 3, 4, 5EFG, and 7F. Please present a summary Excel that shows that the assumption of homogeneity of variance was not violated to justify these parametric tests.***

**Reply:** We thank the reviewer for this important methodological point. To ensure the appropriateness of the parametric tests used in Figures 1D, 2D-F, 3-5, 6E, F, K, and 7F, I, J, we formally tested the assumption of homogeneity of variances before comparing means. We added a statistical summary table as an New Appendix Table. We also clarified the details of statistical analysis in the Materials and Methods section and corresponding figure legends *"All data except Figs. 2H-N, 4C-E, and 7C-F in this study were tested for normality using the Shapiro-Wilk test before proceeding with further statistical comparisons. For the normally distributed datasets, independent t-test or one-way ANOVA followed by Tukey's HSD post hoc test was used, and data were presented as mean ± s.e.m. If the homogeneity of variance assumption was violated in normally distributed datasets, independent t-test with Welch's correction or Welch's one-way ANOVA followed by Games-Howell post hoc test was used. For the datasets without normal distributions, the Mann-Whitney U test or Kruskal-Wallis one-way ANOVA followed by Dunn's pairwise multiple comparisons test were employed for analysis, and data were presented as median ± interquartile range. For the within-group comparisons in Fig. 2E-G', paired t-test and Wilcoxon signed rank test were used, respectively, in datasets with and*

*without normal distribution. The statistical methods used to compare means and their detailed descriptions are summarized in Appendix Table 1. The statistical methods used for each analysis are described in the corresponding figure legends. Statistical analysis was performed with SPSS (IBM, version 21). As for the data in Figs. 2H-N, 4C-E, and 7C-F, statistical analysis was performed with MATLAB built-in functions." on page 35.*

***5) Only qualitative analyses are applied to the data in Figs. 5 and 6. The number of cells, as well as the intensities of VGlut1 and FoxP2, should be quantitatively analyzed.***

**Reply:** We thank the reviewer for this important suggestion. We apologize if this was not sufficiently clear in the original submission. Quantitative analyses of Vglut1 and Foxp2 signal intensities were already included in Figures 5 and 6. To improve clarity, we have revised the Figure Legends and Results text to explicitly indicate these quantifications. In addition, we have now included a quantitative analysis of Foxp2-positive cell numbers in new Figure 6E. We found that Foxp2-positive cell density was unchanged after chronic activity manipulation. We added the description in the Result section *"Note that the Foxp2+ cell density was unchanged, suggesting the reduced intensity of Foxp2 may result from activity-dependent regulation of its expression levels, rather than neuronal loss or altered cell fate (Fig. 6E)."* on Page 16. Regarding Vglut1, as it primarily labels axonal terminals rather than neuronal somata, it is not technically feasible to reliably quantify the number of mCherry+/Vglut1+ labeled cells.

***6) In Figure 7I and 7J, WT control group should be set as 1.0.***

**Reply:** We thank the reviewer for this suggestion. We understand that setting the WT group as 1.0 would facilitate interpretation. However, in Figures 7I and 7J (new Figures 7N and 7O), a WT control group was not included in these specific experiments. Importantly, increased Foxp2 expression relative to WT has already been validated at the protein level in Figure 7F. The analyses shown in Figures 7N and 7O were designed as independent validations at the mRNA level rather than as a direct WT comparison. Including a WT control in these assays would require repeating the entire experimental set in Figures 7N and 7O, as these measurements were performed in parallel batches. To avoid potential confusion, we have now explicitly labeled the data as "fold change" in Figures 7N and 7O and clarified this point in the figure legend and Results section.

***Referee #3:***

***The manuscript shows very nice work on the neural mechanisms involved in neonatal mouse vocalizations induced by maternal separation and on cortico-striatal behavioral effects and plasticity. The work is impressive due to several reasons. It confirms the relevance of prefrontal cortical activity for USV generation. It involves implantation and fiberphotometry in neonatal pups, which is not done a lot in the field. And, the authors show activity dependent wiring in the striatum and an upregulation of FoxP2 following sustained prefrontal cortex activation. They even show that this manipulation can rescue vocal deficits of FoxP2 mutant mice, which has strong translational potential.***

***I suggest that this article will be published in EMBO Reports, however, I have one major and multiple minor issues that the authors should address before publication.***

***Major: The authors claim that cortico-striatal projections are responsible for USV generation (Figures 1-4). However, in none of these experiments the authors specifically investigated or manipulated cortico-striatal projections. In Figure 1 fos activated cells were counted. In Figure 2 the authors used CamKII promoter AAV to image prefrontal cortex activity and in Figures 3 and 4 the authors use a layer V specific mouse line. While layer V contains cortico-striatal projection neurons the observed effects could also be mediated by other projections. Although the activity dependent wiring and upregulation shown in Figures 5-7 suggests that the effect is indeed driven by cortico-striatal projection neurons the effect on vocalizations (Figures 1-4) cannot be proven by this. <u>I strongly suggest to use a retrograde AAV approach</u>***

***(e.g. AAV-retro-Cre in striatum and AAV-DIO-DREADD in PL in WT mice).***

**Reply:** We thank the reviewer for this important suggestion. We agree that the experiments in the original manuscript did not directly manipulate cortico-striatal projection neurons, and therefore could not definitively attribute the observed behavioral effects to this specific projection. To address this concern, we have performed the experiment suggested by the reviewer using a retrograde viral strategy to selectively manipulate cortico-striatal projection neurons. Specifically, we injected *AAV2rg-Cre* into the striatum and Cre-dependent DREADD (*AAV8-hSyn-DIO-DREADD-mCherry*) into the vmPFC, thereby restricting DREADD expression to vmPFC neurons projecting to the striatum. After recovery, animals received CNO (1 mg/kg) prior to USV recording. The results closely recapitulate those observed with direct vmPFC DREADD manipulation. Activation of cortico-striatal projection neurons with hM3Dq significantly increased USV call number, and also altered temporal features of vocalizations, including sequence structure and call-type usage, compared with control animals. These new data are now included in Figure 3, where they are presented alongside the acute vmPFC DREADD manipulation experiments. The findings provide direct evidence that vmPFC neurons projecting to the striatum are sufficient to modulate neonatal USV production, supporting the interpretation that the cortico-striatal pathway contributes to the regulation of vocal behavior. The manuscript text has been revised accordingly "*Although a high percentage of Cre-expressing neurons were labeled in layer V projection neurons in the Rbp4-Cre mice (Gerfen et al., 2013; Munz et al., 2023), only a subset of layer V neurons project to the striatum (Reiner et al., 2010). To directly test whether corticostriatal projection neurons contribute to USV production, we next selectively manipulated vmPFC neurons that project to the striatum using an intersectional retrograde viral strategy. AAV2rg-Cre was injected into the striatum of wildtype mice, together with Cre-dependent DREADD viruses delivered to the vmPFC to specifically target corticostriatal projection neurons (Fig. 3E). Robust mCherry expression was observed in vmPFC, confirming successful labeling of corticostriatal neurons (Fig. 3F). Activation of these neurons significantly increased the total number of USV calls, sequences, and bouts compared with control animals, whereas inhibition showed no significant effect (Fig. 3G; Dataset EV3E). Activation also increased bout span. In addition, the sequence span and the mean number of calls per sequence were significantly higher in the hM3Dq group than in the hM4Di group. These results demonstrate that selective activation of vmPFC corticostriatal projection neurons is sufficient to increase USV production and alter its temporal organization.*" on Page 12.

***Related and minor comments:***

***1) In Figure 1 the authors nicely analyze different areas and find that PL is mostly active in vocalizing mice. However, they did not provide a quantitative analysis for the layers but just show an example image (Fig1E). This has to be analyzed more deeply.***

**Reply:** We thank the reviewer for this important suggestion. In response, we have now expanded the quantitative analysis of cortical layers. Specifically, we increased the sample size and performed layer-specific quantification of Ctip2-positive neurons in Cg, PL, and IL regions. The results are presented in the New Fig. 1H, and the manuscript was revised *"Additionally, we observed a higher density of retrograde-labeled EGFP+ cells in Layer V of PL and Cg (Fig. 1F, G, H), which is in good accord with the prominent corticostriatal projections in Layer V of the cortex (Reiner et al., 2010)."* on page 7. Our analysis revealed significant differences between groups in Layer V of the PL and Cg regions, but not in Layer II/III. These findings indicate that the observed differences are layer-specific and predominantly involve deep-layer projection neurons. This layer-restricted effect further supports our subsequent use of Rbp4-Cre mice, which selectively target Layer V neurons, to investigate the functional contribution of these projection neurons to vocalization behavior.

***2) Figure 2G-L shows a sophisticated analysis using various dimensional reduction techniques, which at first glance does not add too much to the conventional analysis performed in Fig2A-F'. I then, however, understood that the authors tried to predict USVs solely by the neuronal traces. Fig2L shows a reconstructed fluorescence signal that seems to capture the kinetics of the real data shown in Fig2B. However, despite the authors state "a***

***discriminative power in predicting USV occurrence from neural trace dynamics" they do not provide a panel showing the accuracy of USV prediction by their tools. If I'm not mistaken the authors should train a support vector machine with the reduced components to get a proper classifier / predictor. If they can also show that their LDA method works fine it's ok but they then have to show the decoding accuracy (incl. false positives and negatives) in a test data set, which is distinguished from the training data set.***

**Reply:** We thank the reviewer for this constructive suggestion. We agree that demonstrating decoding performance using an explicit classifier and independent validation strengthens the interpretation of our dimensionality reduction analyses. In response, we trained a linear support vector machine (SVM) using the first 10 principal components derived from PCA (fit on the training dataset only). Importantly, classifier performance was then evaluated on an independent held-out dataset that was not used in model fitting or parameter tuning. On this held-out dataset, the linear SVM achieved an AUC of 0.79, an overall classification accuracy of 70.5%, a false positive rate of 9.1%, and a precision of 83.8%. These results demonstrate that peri-USV neural dynamics contain reproducible predictive information that generalizes beyond the training dataset. We have added the corresponding ROC curve to new Figure 2K and in the revised manuscript *"To further assess whether this predictive information generalizes beyond the training dataset, we trained a linear support vector machine (SVM) classifier using PC1-PC10 derived from the same PCA transformation and evaluated its performance on an independent held-out dataset that was not used for model fitting or parameter optimization. The held-out ROC analysis yielded an AUC of 0.79 (Permutation test: P < 0.001; Fig. 2L), with an overall accuracy of 70.5%. The classifier exhibited high precision (83.8%) and a low false-positive rate (9.1%), indicating that predicted USV events were reliable, although recall was moderate (49.2%), reflecting a relatively conservative classification at the selected decision boundary. Together, these results indicate that peri-USV neural dynamics contain information predictive of USV events, and that this predictive structure generalizes to independent datasets."* on Page 10.

***3) In Figure 3A the authors state "Corticostriatal neuron-specific Cre". If I'm not mistaken Rbp4 is a layer V marker contains not exclusively striatal projection cells. Thus, with the methods in Figure 3 the authors manipulate corticostriatal neurons but potentially also other projection neurons. The authors should thus rewrite this or include an experiment in which they inject retrograde Cre-dependent chemogenetic AAVs in the striatum.***

**Reply:** We thank the reviewer for this important clarification. The reviewer is correct that *Rbp4-Cre* labels a subset of layer V pyramidal neurons, including corticostriatal projection neurons, but may also label neurons projecting to other subcortical targets. Therefore, the original wording describing these neurons as "corticostriatal neuron-specific" was not precise. Following the reviewer's suggestion, we have now performed an additional intersectional retrograde viral experiment to directly target corticostriatal projection neurons. In this experiment, *AAV2rg-Cre* was injected into the striatum, and a Cre-dependent DREADD virus was injected into the vmPFC, restricting DREADD expression specifically to vmPFC neurons projecting to the striatum. Manipulation of these neurons produced effects on USV production that were consistent with those observed in the *Rbp4-Cre* experiments. These results are now included in Figure 3, providing direct evidence that corticostriatal projection neurons are sufficient to modulate neonatal vocalizations. In addition, we have revised the text throughout the manuscript to avoid describing *Rbp4-Cre* as corticostriatal neuron-specific, and we now explicitly note this limitation in the Discussion on Page 24. "*The specificity of the genetic tools used to manipulate prefrontal projection neurons is another concern. Although Rbp4-Cre mice target a subset of layer V pyramidal neurons, these neurons are not exclusively corticostriatal and may also include projections to other subcortical regions. To address this concern, we performed an additional intersectional retrograde viral strategy that restricted DREADD expression to vmPFC neurons projecting to the striatum. This manipulation produced effects on USV production similar to those observed in the acute Rbp4-Cre experiments. Nevertheless, we cannot completely exclude the possibility that other vmPFC-fugal pathways may also contribute to the regulation of neonatal vocalizations.*"

***4) The text corresponding to Figure 4 is headed by this subtitle: "Chronic activation of layer V vmPFC neurons during postnatal development increases corticostriatal innervation". However, neither in the Figure or in the text I find experiments in which the corticostriatal innvervation was really investigated. The experiments in Figure 4 show that chronic chemogenetic manipulation leads to increase of USVs numbers and a decrease of USV durations and frequency jumps (Figure 4B), which has nothing to do with corticostriatal innervation. The authors again should either rewrite or provide respective experiments.***

**Reply:** We thank the reviewer for pointing out this issue. We agree that the subtitle in the original manuscript was not specific, as the experiments in Figure 4 assess the behavioral consequences of chronic activation of layer V vmPFC neurons during postnatal development but do not directly measure corticostriatal innervation. Following the reviewer's suggestion, we have revised the subtitle and corresponding text to avoid implying that these experiments directly demonstrate increased corticostriatal innervation. In addition, to more directly address the role of corticostriatal projections, we performed an additional intersectional retrograde viral experiment in which *AAV2rg-Cre* was injected into the striatum and a Cre-dependent DREADD virus was injected into the vmPFC, thereby restricting manipulation to vmPFC neurons projecting to the striatum (new Fig. 3E-G). Manipulation of these neurons produced effects on USV production consistent with those observed in the original experiments. These new data are now included in Figure 3, and the manuscript text has been revised accordingly.

***5) In Figure 5 the authors indeed investigate corticostriatal projections. The result is very interesting but the authors should be careful to state that the VGLUT1+ puncta are corticostriatal projection fibers. <u>As the authors cannot be 100% sure that these fibers may just pass-by the should avoid the term innervation in the respective subtitle.</u> In the text below the authors even state "Since chronic activation of vmPFC corticostriatal neurons increased synaptic connectivity [...]". Again, counting projection fibers is not sufficient to make a statement about synaptic connectivity and experiments have to be provided if the claim wants to be held up (additional pre-postsynaptic marker stainings, electron microscopy or ex-vivo patching).***

**Reply:** We thank the reviewer for this important point. We agree that the wording in the original manuscript overstated the interpretation of these data. Accordingly, we have revised the text to avoid implying direct evidence for synaptic connectivity. Specifically, we replaced the phrase "synaptic connectivity" with "putative glutamatergic synapses" throughout the manuscript. In this experiment, we quantified Vglut1-positive puncta within the striatum. Vglut1 is localized to presynaptic glutamatergic terminals and is not detected along axonal shafts, resulting in a punctate signal corresponding to presynaptic sites. Therefore, the increase in Vglut1 puncta observed following chronic vmPFC activation is consistent with an increase in the number of putative glutamatergic presynaptic terminals within the striatum. Additionally, previous studies have shown that glutamatergic inputs to the striatum are largely segregated by vesicular glutamate transporter subtype, with Vglut1-positive terminals originating predominantly from cortical neurons, whereas Vglut2-positive terminals arise primarily from thalamostriatal projections (Fremeau et al., 2001; PMID: 11502256; Nakamura et al., 2005; PMID: 16217795). Thus, the Vglut1 puncta quantified in our experiments likely represent cortical glutamatergic terminals in the striatum. However, we agree that these data do not directly demonstrate functional synaptic connectivity. The text and figure subtitle have therefore been revised to avoid the term "innervation", and the results are now described more conservatively.

***Referee #4:***

***This study seeks to understand the role of the ventromedial prefrontal cortex in regulating the production of isolation USVs in mouse pups, as well as in regulating synapse formation and gene expression in the striatum. The authors use the TRAP2 strategy to label striatum-projecting cortical neurons that are active in association with isolation USV production. Next,***

***they perform fiber photometry to test whether vmPFC-striatal neurons increase their activity during times of isolation USV production. Then they test the effects of chemogenetic manipulations of activity (Rbp4-expressing vmPFC neurons) on isolation USV production. They test the effects of chronic chemogenetic manipulations on isolation USV production and VGlut1 and FoxP2 expression in striatum. Finally, they test the effects of chronic chemogenetic activation of vmPFC-striatal neurons on isolation USV production in FoxP2 heterozygote mutant mice. Although their experiments reveal interesting effects on isolation USV production, including in FoxP2 mutants, the specificity of these effects to vocal behavior is not established. Overall, many of the conclusions are incompletely supported by the data, some analyses are flawed and/or incomplete, and some experiments are missing important controls.***

***Major***

***Introduction***

***(1) In the Introduction, the authors' description of the two vocalization pathways differs in important details from the description offered by Jurgens, 2009. In Jurgens's view, the first pathway includes ACC to PAG to premotor neuron pools. The second pathway includes motor cortex (as well as inputs to motor cortex), which bypasses the PAG and instead projects directly to premotor neuron pools. It is possible that the authors disagree with some details of this model of vocal motor circuits. Their language, however, suggests that they are summarizing Jurgens's view of these pathways, despite the mismatch between their descriptions and those of Jurgens. This section should be corrected/clarified.***

**Reply:** We thank the reviewer for pointing out the inconsistency between our description and the classical framework proposed by Jürgens (2009). In the revised manuscript, we have clarified this section to more accurately reflect the original model. Specifically, Jürgens proposed two partially distinct vocal motor pathways in mammals: (1) a limbic pathway in which the anterior cingulate cortex projects to the periaqueductal gray (PAG) and then to brainstem premotor circuits controlling vocal motor neurons, and (2) a motor cortical pathway that can influence premotor neuron pools more directly and does not necessarily require PAG involvement. We have revised the text to explicitly distinguish the classical framework proposed by Jürgens from more recent rodent studies demonstrating modulatory roles of additional forebrain structures (e.g., POA, amygdala, and medial prefrontal cortex) that can regulate vocalization through PAG-dependent mechanisms. These revisions clarify that our description reflects both the classical conceptual model and subsequent experimental findings in rodents. The revised section of the Introduction is now on Page 3 "*Classical models of mammalian vocal control propose two partially distinct neural pathways (Jurgens, 2009). The first pathway originates from limbic cortical regions, such as the anterior cingulate cortex (ACC), and relays through the periaqueductal gray (PAG) to brainstem premotor circuits that control vocal motor neurons. The second pathway involves motor cortical projections that can more directly influence brainstem premotor neuron pools and may bypass the PAG. While the classical model emphasizes two partially distinct pathways, subsequent rodent studies suggest that multiple forebrain regions can modulate vocal production through interactions with PAG-dependent circuits. In rodents, studies have revealed additional forebrain structures that modulate vocalization through PAG-dependent gating mechanisms.*"

***(2) In the Introduction, the authors selectively present past work that is in line with their hypothesis (that FoxP2 regulates vocal communication, even in a non-vocal-learner such as the mouse). I don't take issue with the evidence that FoxP2 regulates vocal communication in vocal learners (humans, songbirds). However, the evidence that FoxP2 regulates mouse USV production is complicated. First, FoxP2 mutant mice typically have severe developmental delays. As such, it is not clear whether effects on iso USV production stem directly from changes to circuits that regulate vocalization vs. indirectly from developmental delays. Related to this idea is recent work that found that rates of iso USVs in mouse pups are well related to the intensity of non-vocal movements (Pranic et al., 2021). How can we rule out the possibility that changes in iso USV rates in these FoxP2 models stem indirectly***

***from effects of the FoxP2 mutations on pup non-vocal movement and/or behavioral arousal? Second, the authors do not cite work by Urbanus et al., 2020, who showed that region-specific FoxP2 deletions do not recapitulate the effects of global FoxP2 deletion on iso USV production. This study certainly calls into question the idea that FoxP2 expression in corticostriatal circuits regulates mouse iso USV production. At the very least, these studies/caveats need to be mentioned in the manuscript.***

**Reply:** We thank the reviewer for this thoughtful comment and agree that the relationship between Foxp2 and mouse USV is complex and remains an active area of investigation. In the revised manuscript, we have expanded the Introduction to better reflect the diversity of findings in the field and to acknowledge potential caveats in interpreting USV phenotypes in Foxp2 mutant mice. First, we now describe and discuss the possibility that altered USV production in Foxp2 mutant mice could arise indirectly from developmental delays, changes in motor activity, or altered behavioral arousal, rather than solely from changes in vocalization circuits. In this context, we have added discussion of the work by Pranic et al., 2021, which reported that pup USV production is related to the intensity of non-vocal movements. Second, we have added citation and discussion of Urbanus et al., 2020, which showed that region-specific Foxp2 deletions do not fully recapitulate the USV phenotypes observed in global Foxp2 mutant mice. We now explicitly note that these findings highlight the complexity of interpreting Foxp2 function in mouse vocal behavior. The revised Introduction is now on Page 4 *"Foxp2 mutations in mice impair non-language vocalizations, including isolation-induced ultrasonic vocalizations (USV). Specifically, the call rate and duration of isolation-induced USV are reduced in Foxp2R552H heterozygote and homozygote (Shu et al., 2005; Fujita et al., 2008; Groszer et al., 2008; Gaub et al., 2010), and Foxp2 conditional knockout mice (Kuo et al., 2023). Additionally, other USV features, such as the number of elements, frequency jump, and peak sound pressure, are also impaired in Foxp2 mutant mice (Gaub et al., 2010; Chen et al., 2016; Kuo et al., 2023). However, the mechanistic relationship between Foxp2 and mouse USV remains debated, as alterations in pup USV in Foxp2 mutants may also reflect developmental or motor effects rather than direct changes in vocal circuits (Gaub et al., 2010; Pranic et al., 2022). Consistently, a previous study revealed that region-specific Foxp2 knockout did not cause vocal impairment in call number or temporal features (Urbanus et al., 2020). Interestingly, Foxp2 has been shown to regulate synaptic remodeling through spinogenesis and neurite outgrowth (Schulz et al., 2010; Chen et al., 2016; Kuo et al., 2023), both are known to be activity-dependent. These observations suggest that neuronal activity may regulate Foxp2 expression, which in turn controls synaptic development and remodeling in vocal circuits."*

Importantly, our study approaches this question from a complementary perspective. Rather than relying on constitutive Foxp2 mutations, we combine activity-dependent labeling, circuit-specific manipulation, and behavioral analyses to examine how vmPFC circuits and Foxp2 expression relate to USV production. These approaches allow us to examine the relationship between cortical activity, Foxp2 expression, and vocal behavior without relying solely on developmental genetic models. We also added a paragraph of Discussion on Page 21 "*Previous studies reported that call number and acoustic features may be influenced by developmental delays or alterations in motor activity rather than direct disruption of vocal circuits (Gaub et al., 2010; Pranic et al., 2021). Additionally, region-specific Foxp2 deletions do not fully recapitulate the USV deficits observed in global Foxp2 mutants, suggesting that the relationship between Foxp2 expression and vocal behavior is complex (Urbanus et al., 2020). Our findings provide a complementary perspective on this issue. Rather than relying on developmental genetic models, we manipulated neuronal activity in vmPFC circuits during early postnatal development. Chronic activation of vmPFC neurons increased Foxp2 expression and partially rescued USV production in Foxp2$^{+/-}$ mice. Moreover, Foxp2 expression levels across animals positively correlated with the degree of USV rescue. In addition to changes in call number, we also observed alterations in vocal sequence structure in Foxp2$^{+/-}$ mice. Therefore, chronic activation of vmPFC neurons can modulate USV syntax in both wildtype and Foxp2$^{+/-}$ mice. Notably, such syntax changes were also evident in Foxp2$^{+/-}$ mice following vmPFC activation, suggesting that cortical activity may influence multiple dimensions of vocal behavior, including both call production and vocal sequence organization. These results raise the possibility that Foxp2 functions*

*not only as a developmental transcription factor but also as a mediator of activity-dependent circuit plasticity, thereby influencing vocal communication."*

***TRAP experiments***

***(3) The conclusion that the authors could "tag USV-recruited cell ensembles with reporter gene without significant confounding populations of non-USV cells" is not substantiated. The authors do not include experiments to establish what proportion of the labeled neurons in different regions are related to USVs vs. not USVs, which would be needed to support this statement. It is important to note that neurons labeled in their experimental pups could be activated in relation to any number of things that occur in the iso session and/or after the iso session (USV production, decreased body temperature, increased behavioral arousal, stress of being in a plastic cup, changes in maternal behavior after pup is returned to the nest, etc.). The language here should be modified.***

**Reply:** We thank the reviewer for raising this important point. We agree that neurons labeled during the isolation session could be activated by multiple factors associated with isolation, and not exclusively by USV production. To address this concern, we performed additional validation experiments. First, in wildtype pups we examined the relationship between USV production and c-Fos activation in the vmPFC. We found that the density of c-Fos-positive neurons in vmPFC positively correlated with the number of USV calls produced during the isolation session (new Fig. 1I), supporting the association between vmPFC neuronal activation and USV production. We added this set of experiment on Page 7 *"To further examine the relationship between vocal output and vmPFC neuronal activation, we quantified c-Fos expression in the vmPFC following isolation-induced USV emission. The density of c-Fos+ cells in the vmPFC showed a strong positive correlation with the total number of USV calls across animals (Fig. 1I). This relationship provides independent support that vmPFC neuronal activity is tightly associated with USV emission, indicating the involvement of this region in USV production."*

Second, we performed an activity-dependent reactivation experiment in the setting of the TRAP2 experiment. Neurons activated during the initial USV session were tagged, and their reactivation was assessed during a subsequent USV session. We observed a significantly higher proportion of c-Fos reactivation in previously tagged neurons compared with controls (new Dataset EV1D-F), indicating that neurons recruited during USV production are preferentially reactivated. We added this set of experiment on Page 6 "*To assess whether the tagged neurons represent ensembles that can be reactivated during subsequent vocalization, we performed a reactivation assay (Dataset EV1D-F). At P7, mice received 4-OHT and were separated from the dam 4 hr later to induce isolation stress-induced USVs and label activated neurons. Littermates that remained with the dam served as non-separated controls. On the following day (P8), both groups were subjected to maternal separation to induce USV emission again, and brains were collected 90 min later for c-Fos immunostaining. We observed EGFP and c-Fos-double-positive neurons in the vmPFC of the reactivated group, indicating reactivation of previously tagged neuronal ensembles. Quantification showed that the proportion of c-Fos-positive (+) cells within the EGFP+ population was significantly higher in the reactivated group compared with controls (Dataset EV1D–F).*" Based on these new experiments, we have revised the text to avoid implying that the tagged neurons are exclusively related to USV production and now describe them more conservatively as USV-associated neuronal ensembles throughout the manuscript.

***(4) Related to the above point, why was 4-OHT administered 4 hours before the isolation session? This seems different relative to the optimal time course established in the original TRAP paper. If the authors claim that this timing gave them optimal GFP labeling, they need to show quantification of labeling obtained with different timings of 4-OHT delivery relative to the behavior session. The inclusion of such data would strengthen their claim that their timing of 4-OHT delivery gave optimal neuronal labeling.***

**Reply:** We thank the reviewer for this critical suggestion. To directly address this point, we performed an additional experiment to examine how the timing of 4-OHT administration relative to the

behavioral session affects activity-dependent labeling. Specifically, we injected 4-OHT and then performed the USV isolation session 2, 4, or 6 hours later, followed by quantification of EGFP-positive neurons in the vmPFC. We found that labeling efficiency was significantly higher when the behavioral session occurred 4 hours after 4-OHT injection than in the 2-hour condition, whereas the 4-hour and 6-hour conditions showed comparable labeling efficiency. These data indicate that a 4-hour interval between 4-OHT administration and the behavioral session provides efficient activity-dependent labeling, supporting the timing used in our experiments. These new data are now included in the new Dataset EV1A-C. We added these sets of experiments on Page 6 *"Retrograde AAV2rg-hSyn-DIO-EGFP viruses were injected into the striatum to label the activated upstream neurons of the striatum during maternal separation (Fig. 1A, B). To determine the optimal temporal window for activity-dependent labeling, 4-hydroxytamoxifen (4-OHT) was administered at different intervals relative to USV exposure (2, 4, or 6 hr; Dataset EV1A). Consistent with the short half-life of 4-OHT (6 hr in adult mice), EGFP labeling was markedly increased at the 4-hr interval compared with 2 hr and showed a slight reduction at 6 hr (Dataset EV1B-C). Therefore, 4-OHT was administered 4 hr before the USV assay in subsequent experiments to tag USV-recruited neuronal ensembles with minimal confounding activity."*

***(5) The authors show that the number of GFP-labeled vmPFC neurons was higher in experimental pups than in controls, but this finding does not fully support their conclusion that the labeled neurons are USV-related. As mentioned above, many things differ between the experimental and control groups. The authors should include an analysis within the experimental group, to test whether the number of labeled vmPFC neurons is positively correlated to the number of iso USVs produced across different experimental pups. Such an analysis would substantially strengthen the authors' claim that the labeled neurons are related to USV production.***

**Reply:** We thank the reviewer for this helpful suggestion. To address this point, we performed an additional analysis examining the relationship between USV production and vmPFC neuronal activation across individual pups. Specifically, in wildtype pups we quantified c-Fos-positive neurons in the vmPFC following the isolation session and tested whether their number correlated with the number of USV calls produced by each pup. We found that the density of c-Fos-positive neurons in the vmPFC was positively correlated with the number of USV calls (new Fig. 1I; $r = 0.82$, $P = 0.0071$), indicating that pups producing more USVs showed stronger vmPFC activation. These results support the association between vmPFC neuronal activity and USV production, consistent with the interpretation that neurons recruited during the isolation session are related to vocal behavior. We add this set of experiment on Page 7 *"To further examine the relationship between vocal output and vmPFC neuronal activation, we quantified c-Fos expression in the vmPFC following isolation-induced USV emission. The density of c-Fos+ cells in the vmPFC showed a strong positive correlation with the total number of USV calls across animals (Fig. 1I). This relationship provides independent support that vmPFC neuronal activity is tightly associated with USV emission, indicating the involvement of this region in USV production."*

***(6) The authors should validate their findings from the TRAP labeling experiment with a Fos staining? Is Fos expression in vmPFC-striatal neurons positively correlated with iso USV rates in experimental pups? The time window of TRAP2 labeling is really long (as noted by the authors, many hours) compared to the time resolution of Fos immunohistochemistry.***

**Reply:** We thank the reviewer for this important issue. To provide an independent validation with higher temporal resolution, we performed an additional experiment examining c-Fos activation in the vmPFC following the isolation-induced USV session. Wildtype pups were subjected to the USV isolation assay and perfused 90 minutes after the behavioral session, followed by quantification of c-Fos-positive neurons in the vmPFC. We then tested whether neuronal activation correlated with the number of USV calls produced by each pup. We found that vmPFC c-Fos-positive cell density positively correlated with USV call number (new Fig. 1I; $r = 0.82$, $P = 0.0071$), indicating that pups producing more USVs showed stronger vmPFC activation. Because c-Fos immunostaining provides

a shorter temporal integration window compared with TRAP2 labeling, these data independently support the association between vmPFC neuronal activity and USV production.

***(7) The included cortical regions are large. Which and how many planes of section were considered and/or averaged in these quantifications (Fig. 1D)? The inclusion of these details is critical to evaluate the data.***

**Reply:** We thank the reviewer for pointing this out. *"Specifically for the TRAP experiment, coronal brain sections were cut at 20 μm thickness from the frontal cortex posteriorly to the level at which the septum disappears (approximately corresponding to the emergence of the hippocampal commissure), spanning about 2 to 2.3 mm along the rostro-caudal axis depending on brain size. Sections were collected sequentially into 12 parallel series (inter-section interval of 240 μm within each series). One systematically spaced series was selected for immunostaining to ensure unbiased sampling across the full rostro-caudal extent of the region of interest. As a result, approximately 8-10 sections per brain were quantified for analysis."* The description was added in the Materials and Methods section in the revised manuscript.

***Fiber photometry***

***(8) Previous work has found that rates of pup iso USVs are correlated with the intensity of body movements (Pranic et al., 2021). Were videos collected of the pup's behavior during fiber photometry sessions? Can you rule out the possibility that vmPFC signals are related to body movements/behavioral arousal?***

**Reply:** We thank the reviewer for raising this important point. Video recordings were not collected during the fiber photometry sessions. We acknowledge that isolation-induced USVs in neonatal pups are typically accompanied by body movements, making complete dissociation of vocalization from arousal or motor output challenging. The photometry signals were processed using the manufacturer's motion artifact correction pipeline, which includes isosbestic regression-based correction of movement-related fluctuations. This reduces the likelihood that the observed USV-aligned signal reflects gross mechanical artifacts. However, we recognize that motion correction does not exclude the possibility that vmPFC activity may be related to motor execution or behavioral arousal associated with vocalization. Moreover, the calcium dynamics were temporally aligned to USV onset and required at least 2 seconds of preceding silence, minimizing contamination from ongoing activity. Nevertheless, without simultaneous behavioral tracking, we cannot fully dissociate vocal-related neural activity from movement-related components. We have now explicitly discussed this limitation in the revised manuscript on page 24 *"Another limitation is the absence of simultaneous behavioral tracking during experimental sessions, which prevented direct quantification of movement or general arousal states. Because our analyses focused on vocalization-aligned neural dynamics and USV output, we cannot fully exclude the possibility that some observed effects may partially reflect motor- or arousal-related processes associated with vocal behavior. Notably, following chemogenetic manipulation, we did not observe overt changes in general behavioral responsiveness, posture, or body condition during testing sessions compared with the control group, suggesting that the primary effects were related to vocal output rather than gross alterations in overall activity or physical state."*

***(9) The authors should include GFP controls, to rule out the possibility that some signals resulted from movement artifacts. The shuffled control group does not allow the authors to test this point or the previous point.***

**Reply:** We thank the reviewer for this suggestion. As a control for potential motion-related artifacts, we performed a new set of GFP-only recordings under identical experimental conditions (Dataset EV2A). In these animals, no USV-aligned fluorescence changes were observed, indicating that the peri-USV signals detected in GCaMP6 recordings are unlikely to reflect nonspecific optical or motion artifacts. In addition, photometry signals were processed using isosbestic regression-based motion artifact correction to reduce movement-related fluctuations prior to analysis. We agree that shuffled controls alone cannot fully exclude movement-related neural contributions. Rather, the shuffled

analysis was included to demonstrate the temporal specificity of USV-aligned dynamics. Together with the GFP control and motion correction procedures, these data support the conclusion that the observed signals are not driven by gross movement artifacts. We added this experiment on Page 8 *"As a control for potential fluorescence artifacts, fiber photometry recordings were performed in mice injected with AAV9-CaMKII-EGFP in the same neuronal population. GFP signals showed no change during USV production, indicating that the observed GCaMP signals reflect calcium-dependent neuronal activity (Dataset EV2A-A')."*

***(10) The authors should include representative traces of raw data (example periods of photometry signal along with either a spectrogram of USVs or a raster plot showing the timing of USVs). Without seeing any raw data, it is difficult to understand to what degree vmPFC-striatal activity was selectively related to USV production vs. behavioral events that occur outside of USV times.***

**Reply:** We appreciate the reviewer's request to include representative raw traces. We have now added an about 20-second example of fiber photometry signal aligned with a USV raster plot (New Fig. 2B). This example illustrates that increases in vmPFC calcium activity are temporally associated with USV production rather than nonspecific elevation. Importantly, during adjacent non-USV periods within the same recording segment, similar ramping dynamics are not observed. In addition, the event-aligned analyses (Fig. 2C-G) quantitatively demonstrate that peri-USV activity differs significantly from shuffled controls, further supporting that the observed activity patterns are selectively related to USV production rather than general behavioral fluctuations. The manuscript was also revised accordingly on Page 8 *"The vmPFC calcium dynamics were then aligned to USV onset, revealing temporally structured changes in activity surrounding vocalization events (Fig. 2B). To avoid overlap between adjacent vocalization-related signals, we restricted the data analysis to USV events with inter-event intervals longer than 2 seconds from the preceding one (red-labeled events in Fig. 2B)."*

***(11) I'm not following the purpose of the PCA/t-Sne analyses of photometry signals collected around USV times vs. non-USV times. The non-USV times would be expected to contain a random smattering of transients (presumably, there are some transients outside of USV times, see previous point) and non-transients. One would expect USV times vs. non-USV times to look different, but why is that interesting?***

**Reply:** We appreciate the reviewer's question regarding the conceptual purpose of the PCA and nonlinear embedding analyses. Our goal was not simply to demonstrate that USV and non-USV periods differ in mean amplitude, which would indeed be expected. Rather, we aimed to determine whether peri-USV calcium dynamics exhibit a structured and reproducible temporal signature that can be distinguished from randomly occurring transients outside vocalization periods. If the observed USV-related signal merely reflected random and spontaneous calcium transients, we would not expect the peri-event traces to cluster in low-dimensional space or to support reliable classification. Instead, PCA revealed consistent temporal components accounting for most of the variance, and both unsupervised embedding (UMAP/t-SNE) and supervised decoding (LDA in Fig. 2J, K and SVM in New Fig. 2L) demonstrated separable structure between USV and non-USV periods. These analyses therefore support the interpretation that vmPFC activity during USV production represents a distinct dynamic state rather than a stochastic accumulation of unrelated calcium events.

***(12) In Fig. 2B, the summary data should be the average of the averages for the n = 4 mice. It appears from the description that the authors have instead averaged across all USV onsets (n = 170 evens). This is a flawed way to perform the analysis, as the average shown could be the result of one mouse with a great relationship between USVs and vmPFC activity and 3 mice with a poor/weak relationship.***

**Reply:** We appreciate the reviewer's concern regarding the appropriate unit of analysis. We agree

that the mouse is the independent experimental unit for between-animal inference. Accordingly, we now exchanged the order of figure and explicitly present mouse-level quantification (Fig. 2E, F, G; n = 4 mice) in which each data point represents the mean response per mouse (i.e., the average across that mouse's USV events). These mouse-level analyses confirm the same effect observed in the event-level distributions. We retain the event-level plots (Fig. 2E', F', G'; n = 171 events) to illustrate within-mouse variability and the distribution across individual USV events, but we clarify in the text and figure legend that statistical inference regarding reproducibility across animals is based on the mouse-level analyses. In addition, we now show the USV-aligned calcium traces from all four individual mice (Fig. 2C'), demonstrating that the USV-associated vmPFC activity is consistently observed across animals rather than being driven by a single subject. Now the manuscript was revised on Page 8 "*We observed a sharp increase in calcium transients immediately before USV emission (Fig. 2C-D). To confirm the specificity of population-level responses in response to USV emission, we generated a shuffled control dataset by randomly assigning timestamps and analyzing their corresponding calcium transients. This shuffled control group allowed us to compare the observed calcium transients with those that would otherwise occur by chance (Fig. 2C-D). We found that the slope of calcium traces (± 0.25 sec from onset) was significantly higher in the aligned USV group compared to the shuffled control group (Fig. 2E-E'). As for the change in neuronal activity before and after USV onset, both the area under curve (1 sec from onset, AUC) of the calcium transient activity and the peak fluorescence activity following USV onset were significantly higher than those preceding USV (Fig. 2F-G').*"

***(13) It is somewhat unexpected that the averaged USV-aligned photometry signal decays in Fig. 2B and 2B' begins to decay so rapidly. Pups often produce bouts of iso USVs. It would strengthen this results section to separately analyze photometry signals aligned to onsets of single iso USVs (i.e., iso USVs that are preceded by and followed by silence) vs. bouts of USVs (multiple USVs back-to-back) to understand whether the temporal dynamics of the photometry signals differ according to bout length.***

Reply: We thank the reviewer for raising this important point regarding bout structure and signal dynamics. In our analysis, we restricted inclusion to USV events that were preceded by at least 2 seconds of silence. This criterion was applied to prevent contamination of baseline ΔF/F calculations by preceding vocalization-related activity. Importantly, in neonatal mice, USV bouts are operationally defined by inter-event intervals longer than 2000 ms (Möhrle et al., 2023). Thus, our inclusion criterion effectively isolates the first USV of each bout. Therefore, the photometry traces shown in the New Fig. 2B predominantly reflects activity aligned to bout-onset USVs rather than to intra-bout calls. The rapid post-onset decay observed in the averaged signal likely reflects the transient neural response associated with bout initiation rather than sustained activity across subsequent calls within the bout. We have clarified this definition in the revised Methods and figure legend to make this distinction explicit.

***Acute chemogenetic manipulations of activity***

***(14) In Figure 3C, why does the viral labeling in the mCherry group look so sparse compared to the labeling in the hM3Dq group? It is also concerning that nearly every mCherry-positive neuron expresses Fos in the control group. This shouldn't be the case.***

**Reply:** The apparent difference in labeling density between the mCherry control and the hM3Dq-mCherry group reflects differences in subcellular localization. The control construct expresses cytosolic mCherry, whereas hM3Dq-mCherry is membrane-bound, which results in prominent labeling of neuronal processes and the boundary of soma. This membrane-associated fluorescence increases the apparent signal density and can visually exaggerate the extent of labeling, even when the number of infected somata is comparable. In the original figure, we used lower-magnification images to illustrate the overall distribution of c-Fos-positive activated neurons. To avoid potential misinterpretation, we have now replaced these panels with higher-magnification images that allow clearer visualization of individual cells (New Fig. EV3B1-C5). Regarding the concern that nearly

every mCherry-positive neuron appears Fos-positive in the control group, our re-examination of the images indicates that this is not the case. Multiple mCherry-positive neurons do not express cFos in the control condition. To prevent misinterpretation, we have revised the figure and now indicate representative mCherry-positive/Fos-negative neurons with white arrowheads in both groups. The figure legend has also been updated to clarify this point.

***(15) Were videos collected? Can the authors rule out the possibility that changes in iso USV rates were secondary to changes in movement and/or behavioral arousal?***

**Reply:** We thank the reviewer for raising this issue again. We did not collect video recordings during these experiments. Therefore, we cannot directly quantify movement or behavioral arousal. However, isolation-induced USVs in neonatal pups are intrinsically linked to motor output, and we did not observe overt differences in gross behavioral state across groups during testing. Importantly, the acute chemogenetic manipulations selectively altered USV production without altering overall behavioral responsiveness. We acknowledge that detailed behavioral quantification would strengthen this conclusion and now explicitly note this as a limitation on Page 24 *"Another limitation is the absence of simultaneous behavioral tracking during experimental sessions, which prevented direct quantification of movement or general arousal states. Because our analyses focused on vocalization-aligned neural dynamics and USV output, we cannot fully exclude the possibility that some observed effects may partially reflect motor- or arousal-related processes associated with vocal behavior. Notably, following chemogenetic manipulation, we did not observe overt changes in general behavioral responsiveness, posture, or body condition during testing sessions compared with the control group, suggesting that the primary effects were related to vocal output rather than gross alterations in overall activity or physical state."*

***(16) I understand that optogenetics is more challenging than chemogenetics to implement in pups. However, the conclusion that vmPFC activation promotes USVs in a relatively direct fashion would be strengthened if the authors could show that vmPFC opto activation elicits USVs (during/following laser-on periods).***

**Reply:** We thank the reviewer for this important suggestion. We agree that temporally precise optogenetic activation would provide stronger evidence for the sufficiency of vmPFC activation in driving USV production. However, implementing optogenetic stimulation in neonatal mice presents substantial technical and ethical challenges. Neonatal cranial implantation carries a high surgical failure and mortality rate, particularly beyond the first few postnatal days when hypothermia anesthesia is no longer sufficient and reliable anesthetic monitoring becomes difficult. Indeed, even our fiber photometry experiments required a large number of pups to obtain stable and analyzable datasets, underscoring the fragility of this preparation. In addition, to our knowledge, there are currently no established or validated optogenetic stimulation protocols specifically optimized for neonatal pups, including systematic validation of neuronal responsiveness, light spread, and effective stimulation parameters in the developing brain. Without such validation, interpretation of stimulation-induced behavioral effects would be uncertain. Developing and validating these methodologies would require extensive additional experimentation beyond the scope of the present study. We have now acknowledged this limitation in the Discussion and clarified that future studies using temporally precise manipulations will be important for further delineating the causal dynamics of vocal circuits. *"Finally, the present study did not employ temporally precise manipulations, such as optogenetic activation, which is technically challenging in neonatal brains, to test whether acute vmPFC stimulation is sufficient to trigger USVs. Future studies using temporally controlled approaches will be necessary to further delineate the causal dynamics of this circuit."* on Page 25.

***Chronic chemogenetic activation/inhibition vs. corticostriatal innervation, VGlut1 and FoxP2 expression***

***(17) In Figure 4B, left-most panel, is the number of USVs in the hM4Di group significantly different from controls, as well as from the hM3Dq group? If not, rather than saying "hM4Di***

***inhibition decreased USV emissions compared to the hM3Dq group", the authors should say that hM4Di inhibition did not change USV emissions relative to controls.***

**Reply:** We thank the reviewer for this helpful comment. We reanalyzed the USV data using an updated USV analysis pipeline and revised the corresponding text accordingly. The updated analysis shows that chronic vmPFC activation significantly increased the total number of calls and sequences, as well as the mean number of calls and sequences within bouts, compared with control mice. In contrast, chronic inhibition of vmPFC neurons did not significantly alter USV emissions relative to controls, but showed lower values compared with the activation group. We have revised the Results section to clarify this point and to avoid the impression that hM4Di inhibition reduces USV emissions relative to controls. The revised text is now on Page13 *"Chronic vmPFC activation significantly increased the total number of calls and sequences, as well as the mean number of calls and sequences within bouts, compared with control mice. In contrast, chronic inhibition of vmPFC neurons reduced USV output relative to the activation group and decreased the probability of short calls (Fig. 4B; Dataset EV4A). No significant difference was observed between the hM4Di and control groups. These data suggest that manipulating the activity of neonatal vmPFC neurons during early development alters subsequent isolation-stress-induced USV in neonatal mice."*

***(18) The results subheading "Chronic activation of layer V vmPFC neurons during postnatal development increases corticostriatal innervation" looks like a mistake, given that this results section only describes effects of chronic chemogenetic manipulations of USV production.***

**Reply:** We thank the reviewer for pointing out this inconsistency. The subheading has been corrected in the revised manuscript to accurately reflect the content of the section.

***(19) Were videos collected? Are the non-vocal behaviors of pups that have experienced chronic activation or chronic inhibition of vmPFC different from controls?***

**Reply:** We thank the reviewer for raising this issue again. Video recordings were not obtained for systematic behavioral comparisons in these experiments. We did not observe qualitative differences in general behavioral state, posture, or reactivity between groups during recording sessions.

To further address this concern, we re-analyzed the chronic DREADD dataset, focusing on vocal call structure/classification and transitions. While we cannot fully exclude effects on general motor output, permutation analyses at the individual-animal level revealed significant changes in entropy rate, self-transition structure, and PCA-based separation of per-mouse transition probability. These measures reflect the statistical organization of vocal sequences rather than simple changes in vocal output quantity. We have now added this data to Figure 4. Nevertheless, we acknowledge that comprehensive behavioral tracking would be required to fully exclude subtle changes in non-vocal behaviors, and this is now discussed as a limitation of the study on Page 24 *"Another limitation is the absence of simultaneous behavioral tracking during experimental sessions, which prevented direct quantification of movement or general arousal states. Because our analyses focused on vocalization-aligned neural dynamics and USV output, we cannot fully exclude the possibility that some observed effects may partially reflect motor- or arousal-related processes associated with vocal behavior. Notably, following chemogenetic manipulation, we did not observe overt changes in general behavioral responsiveness, posture, or body condition during testing sessions compared with the control group, suggesting that the primary effects were related to vocal output rather than gross alterations in overall activity or physical state."*

***(20) The sample size is low for the hM4Di group relative to the others. The number of USVs in the hM4Di group looks like it tends to be lower than controls, and I wonder if the experiment is underpowered.***

**Reply:** We thank the reviewer for raising this concern. To address the potential issue of limited statistical power, we increased the sample size, especially the hM4Di group, by adding additional litters, resulting in 11-14 animals per group in the revised analysis. In addition, to more comprehensively characterize potential differences in vocal behavior among groups, we reanalyzed

the USV recordings using the VocalMat software, which allows automated annotation of call types and extraction of detailed acoustic features. This approach enabled us to perform call-type classification and Markov transition analyses of vocal sequences. Using this refined analysis, we identified additional differences in vocal structure and transition probabilities between groups, particularly between the hM3Dq and hM4Di conditions. These results are now included in the revised manuscript (new Fig. 4B-D) on Page 13 *"To determine whether developmental manipulation of vmPFC activity also alters the syntactic organization of vocalizations across multiple hierarchical levels, we analyzed the sequential structure of USV calls using a first-order Markov chain-based transition analysis (Dataset EV4B). Transition networks constructed from individual call types showed broadly similar connectivity patterns across groups (Fig. 4C; Dataset EV4C). Consistent with this observation, per-mouse permutation testing revealed no significant differences in overall transition patterns among groups. To further quantify global sequence organization, we calculated the entropy rate of the transition matrix, which reflects the diversity of call-to-call transitions. Chronic activation of vmPFC neurons significantly increased the entropy rate compared with controls after FDR correction for multiple comparisons (Fig. 4D), representing a transition toward a more diverse and less predictable vocal repertoire. To test whether total call number confounds the entropy rate analysis, we performed a multiple linear regression with total call number included as a covariate. The group effect remained significant after controlling for total call number ($P = 0.037$), indicating that the increase in entropy was not simply due to increased vocal output. We next examined the self-transition ratio of call sequences, which reflects the probability that consecutive calls are of the same type and therefore captures the tendency of sequences to repeat individual call types. In contrast to the entropy analysis, no significant differences in self-transition ratio were detected between groups after FDR correction for multiple comparisons, suggesting that vmPFC manipulation primarily altered the diversity of call-to-call transitions rather than the repetition of individual call types. To visualize how specific call-to-call transitions contributed to these changes, we examined differential transition probabilities ($\Delta P$) between groups. This analysis revealed distributed increases and decreases across multiple call transitions in the activation group relative to controls (Fig. 4E), consistent with the increased transition diversity reflected by the entropy analysis. Finally, we performed PCA-based comparisons of the global transition probability distribution. PCA of transition matrices revealed significant separation of group centroids in PC space (permutation test, $P = 0.0183$; Dataset EV4D), further supporting that developmental vmPFC manipulation alters the global structure of USV syntax. Together, these results suggest that vmPFC neuronal activity during early development modulates the sequential organization of neonatal vocalizations."* These analyses provide a more detailed characterization of how chronic vmPFC neuronal manipulation affects vocal production and syntax.

***(21) It seems problematic to me that the analyses in Figure 5 were performed at the level of individual cells, rather than by animal. How can we know whether the changes in VGlut1 expression are robust across animals in the hM3Dq experimental group?***

**Reply:** We thank the reviewer for raising this point. In the original analysis, Vglut1 signal was quantified at the single-cell level because only neurons that were directly contacted by mCherry-positive axonal terminals were included in the analysis. This approach allowed us to specifically measure Vglut1 signal at putative synaptic sites associated with axons from manipulated neurons. To address the reviewer's concern regarding statistical independence across animals, we performed an additional analysis using animal-level averages, in which Vglut1 signal was first averaged within each mouse and then compared across groups. The results of the animal-level analysis are now shown in Figure 5E. Consistent with the cell-level analysis, hM3Dq animals showed a higher Vglut1 signal than controls, whereas the hM4Di group showed a tendency toward a reduction that did not reach statistical significance. The new set of analysis is now described on Page 15 *"After chronic hM3Dq activation of vmPFC neurons, we found increased Vglut1 immunoreactivity in DARPP32+ striatal neurons innervated by mCherry+ terminals (Fig. 5C2, E, F) compared to the control group (Fig. 5C1, E, F) at the mouse and cell levels."*

***(22) In Figure 5G, the counts of neurons are too small, and the analysis seems underpowered. Given the small numbers of neurons and the trend that mirrors the findings in Fig. 5E/F (hM3Dq > controls and hM4Di), I don't think the authors' claim that the changes in VGlut expression are selective for striatal neurons innervated by mCherry axons is well supported.***
**Reply:** We thank the reviewer for raising this concern. To address potential limited statistical power, we increased the number of animals analyzed, resulting in a larger number of quantified neurons in the revised dataset. With the expanded dataset, the overall pattern remained consistent with the original observations. Specifically, hM3Dq activation showed a higher level compared with controls, whereas hM4Di-mediated inhibition showed only a tendency toward reduction and did not reach statistical significance. The updated quantification and revised description are included in the revised manuscript.

***(23) The results seem to state that chronic inhibition of vmPFC neurons causes a downregulation of FoxP2 relative to controls. However, Fig. 6E seems to show that hM4Di does not differ from controls. The authors should adjust the language to ensure that the conclusions correspond to the data.***
**Reply:** We thank the reviewer for pointing out this inconsistency. We agree that the wording in the original manuscript was misleading and was not supported by the data shown in old Fig. 6E. We have now revised the manuscript on Page 16 *"The results showed that Foxp2 immunofluorescent signals were significantly upregulated in the hM3Dq group in MOR1+ striosomal neurons innervated by mCherry-expressing axonal terminals (Fig. 6B-F). Note that the Foxp2+ cell density was unchanged, suggesting the reduced intensity of Foxp2 may result from activity-dependent regulation of its expression levels, rather than neuronal loss or altered cell fate (Fig. 6E)."*

***Chronic chemogenetic activation in FoxP2 hets***
***(24) The authors state that they manipulate vmPFC-striatal neurons, but the strategy used in this experiment (Rbp4-Cre) is not specific to that cell type.***
**Reply:** We thank the reviewer for pointing out this issue. We agree that *Rbp4-Cre* labels a subset of layer V pyramidal neurons that are not exclusively corticostriatal projection neurons, and therefore the original wording could have overstated the projection specificity of this manipulation. To address this concern, we performed an additional intersectional retrograde viral experiment in which *AAV2rg-Cre* was injected into the striatum and a Cre-dependent DREADD virus was injected into the vmPFC, thereby restricting DREADD expression to vmPFC neurons projecting to the striatum (new Fig. 3E-G). Manipulation of these neurons produced behavioral effects on USV production similar to those observed in the *Rbp4-Cre* experiments. Now the added experiment is described on Page 12 *"Although a high percentage of Cre-expressing neurons were labeled in layer V projection neurons in the Rbp4-Cre mice (Gerfen et al., 2013; Munz et al., 2023), only a subset of layer V neurons project to the striatum (Reiner et al., 2010). To directly test whether corticostriatal projection neurons contribute to USV production, we next selectively manipulated vmPFC neurons that project to the striatum using an intersectional retrograde viral strategy. AAV2rg-Cre was injected into the striatum of wildtype mice, together with Cre-dependent DREADD viruses delivered to the vmPFC to specifically target corticostriatal projection neurons (Fig. 3E). Robust mCherry expression was observed in vmPFC, confirming successful labeling of corticostriatal neurons (Fig. 3F). Activation of these neurons significantly increased the total number of USV calls, sequences, and bouts compared with control animals, whereas inhibition showed no significant effect (Fig. 3G; Dataset EV3E). Activation also increased bout span. In addition, the sequence span and the mean number of calls per sequence were significantly higher in the hM3Dq group than in the hM4Di group. These results demonstrate that selective activation of vmPFC corticostriatal projection neurons is sufficient to increase USV production and alter its temporal organization."*
In addition, we have revised the text throughout the manuscript to avoid describing the *Rbp4-Cre* manipulation as specific to corticostriatal neurons, and we now explicitly note this limitation in the Discussion on Page 24 *"One limitation of the present study is that c-Fos is not a perfect indicator of neuronal activity, as some active neurons may not express Fos (false negatives), and Fos*

*expression can occasionally occur in the absence of strong neuronal firing or be unrelated to the behaviors we studied (false positives). The TRAP2 experiment should therefore be interpreted as identifying candidate neuronal populations engaged during USV production rather than definitively labeling all neurons involved in vocalization."*

***(25) Were videos collected? In addition to promoting USV production, does chronic chemogenetic activation of vmPFC neurons alter the non-vocal behaviors of FoxP2 mutant mice?***

**Reply:** We thank the reviewer for this critical issue. We did not perform systematic behavioral scoring of non-vocal behaviors in Foxp2 mutant pups following chronic chemogenetic activation. During testing sessions, however, we did not observe overt differences in posture, mobility, or general behavioral reactivity between groups. The primary measurable effect of vmPFC activation was an increase in USV production. We acknowledge that subtle alterations in non-vocal behaviors cannot be ruled out without comprehensive behavioral tracking, which represents a limitation of the current study. Future experiments incorporating detailed behavioral quantification will be required to address this question directly. We added a description of the limitations in the revised manuscript on Page 24 *"Another limitation is the absence of simultaneous behavioral tracking during experimental sessions, which prevented direct quantification of movement or general arousal states. Because our analyses focused on vocalization-aligned neural dynamics and USV output, we cannot fully exclude the possibility that some observed effects may partially reflect motor- or arousal-related processes associated with vocal behavior. Notably, following chemogenetic manipulation, we did not observe overt changes in general behavioral responsiveness, posture, or body condition during testing sessions compared with the control group, suggesting that the primary effects were related to vocal output rather than gross alterations in overall activity or physical state."*

***(26) Does the degree of iso USV rescue correlate with the degree of FoxP2 expression level rescue across animals in the hM3Dq group? If so, this would strengthen the claim that FoxP2 expression might be related in some way to the rescue of iso USV rates.***

**Reply:** We thank the reviewer for this helpful suggestion. To address this point, we examined whether the degree of USV rescue correlated with Foxp2 expression levels across individual animals. Specifically, Foxp2 protein intensity was quantified at the single-cell level in vmPFC neurons from *Foxp2$^{+/-}$;mCherry* and *Foxp2$^{+/-}$;hM3Dq* mice, and Pearson correlation analysis was performed between Foxp2 expression levels and the total number of USV calls produced by each mouse. We found that Foxp2 expression levels were positively correlated with USV call number (new Fig. 7K; r = 0.90, P = 0.0003, n = 10 mice). These results indicate that animals with higher Foxp2 expression levels exhibited stronger rescue of USV production, supporting the association between Foxp2 expression and the behavioral rescue induced by vmPFC activation. We added this experiment on page 19 "*Next, we detected and quantified Foxp2 expression levels in striatal neurons innervated by vmPFC-striatal axonal terminals using immunohistochemistry. We found that Foxp2 immunoreactivity in MOR1$^+$ striosomal neurons innervated by RFP/mCherry+ terminals was increased in Foxp2$^{+/-}$;hM3Dq group compared to Foxp2$^{+/-}$;mCherry group (Fig. 7C-F). Additionally, to determine whether the partial rescue of USV production was associated with increased Foxp2 expression, we examined the relationship between Foxp2 protein levels and vocal output. Foxp2 protein intensity was quantified at the single-cell level in Foxp2$^{+/-}$;mCherry and Foxp2$^{+/-}$;hM3Dq mice, and Pearson correlation analysis was performed between Foxp2 expression and the total number of USV calls per mouse. We found that Foxp2 expression levels were positively correlated with USV call number (Fig. 7K).*"

*Minor*

***(1) The sentence in the abstract that includes "chronic activation of vmPFC-striatal neurons not only increased FoxP2...but also VGlut1-labeled synapses" would be clearer if the authors modified the language to clarify where (in what brain region) these changes were observed.***

**Reply:** We thank the reviewer for this helpful suggestion. We have revised the corresponding

sentence in the Abstract to explicitly specify the brain region in which these changes were observed. The sentence now clarifies that the increases in FoxP2 expression and VGlut1-labeled synapses were detected in the striatum. *"Interestingly, chronic activation of vmPFC neurons not only increased Foxp2, a gene implicated in childhood speech apraxia, but also Vglut1-labeled synapses in the striatum, suggesting that activity-dependent increases in Foxp2 may promote corticostriatal synaptogenesis." on Page 2.*

***(2) In the first sentence of the Intro, I'm not sure what is meant by "vocal plurality".***
**Reply:** We thank the reviewer for pointing out the ambiguity in the original phrasing. We have revised the first sentence of the Introduction to improve clarity. The sentence now reads: *"Vocal communication is generated from complex neural circuits that confer vocal diversity, facilitating intraspecific social bonding and emotional expression in mammals."*

***(3) Based on the findings, I find the title to be misleading, as I don't think the authors have demonstrated that vmPFC-striatal neurons are part of a vocalization circuit***
**Reply:** We appreciate the reviewer's suggestion and have revised the title to better reflect the functional and developmental focus of the study while avoiding overly specific anatomical terminology. We have revised the title to "*Activity-dependent development of vocal circuits in the neonatal rodent forebrain.*"

***(4) In the TRAP experiments, were the subjects males, females, or both?***
**Reply:** We thank the reviewer for pointing out the lack of clarity regarding the sex of the animals. Both male and female pups were included in the TRAP experiments, as now specified in the revised Materials and Methods section. *"Both male and female pups were included in all experiments."*

***(5) Were the measurements of Foxp2 and VGlut1 expression conducted in the same hM3Dq mice, or in two different sets?***
**Reply:** We thank the reviewer for this important clarification. The protein-level measurements of Foxp2 and VGlut1 were conducted in the same cohort of hM3Dq mice. In contrast, mRNA-level analyses, including in situ hybridization and qRT-PCR, were performed in independent cohorts. These experiments were designed to independently validate transcriptional and protein-level changes while avoiding potential confounds from tissue processing requirements.

***(6) In the Discussion, the authors say "Due to the low level of USV events in Rbp4-Cre mice, we did not find a significant reduction in USV emissions in pups with chronic inhibition." I don't find this reasoning to be sound, as the USV rates look high enough to be able to detect a significant decrease with a sufficiently powered dataset.***
**Reply:** We thank the reviewer for this comment. In the original manuscript, we speculated that the absence of a significant reduction in USV production following chronic inhibition might be related to the relatively low baseline USV rates observed in the Rbp4-Cre background. We agree that this explanation was not sufficiently supported by the data. To address this issue, we increased the sample size and reanalyzed the USV recordings using the VocalMat analysis pipeline, which enabled a more detailed characterization of vocal behavior. In the expanded dataset, chronic hM4Di-mediated inhibition did not significantly reduce overall call number compared with controls, although the variability across animals remained relatively high. However, several vocal parameters and syntax differed between the hM4Di and hM3Dq groups. We have therefore revised the Results and Discussion to remove the previous explanation and to describe the findings more conservatively.

***(7) In the Discussion, the authors cite Zheng et al., 2022 and state that vmPFC projects to the vocal motor organ in Alston's singing mice. It is important to clarify that that study used polysynaptic tracing, and that the vmPFC does not project directly to the larynx.***
**Reply:** We thank the reviewer for this important clarification. Zheng et al. (2022) used polysynaptic tracing, which does not demonstrate a direct projection from vmPFC to the larynx. To avoid this

misunderstanding, we have revised the text to read: *"However, a later study using polysynaptic tracing revealed that the prelimbic area of vmPFC is part of the cortical network connected to the vocal motor organ in Alston's singing mouse, suggesting a potential role for vmPFC in vocal production (Zheng et al., 2022)."* on Page 23.

Dear Dr. Kuo,

Thank you for the submission of your revised manuscript. We have now received the enclosed reports from the referees and I am ahppy to say that all support its publication now. Only a few editorial requests will need to be addressed before we can proceed with the official acceptance of your manuscript:

- Please add a "Disclosure and Competing Interest Statement" to the ms file.

- Please add an author list with affiliations and corr. author info on the title page with the next submission.

- The REFERENCE format needs to be corrected: et al needs to be used after 10 author names. Please use the EMBO reports reference format.

- Please upload with your next submission a completed author checklist, which you can download from our author guidelines: https://link.springer.com/journal/44319/submission-guidelines. The completed author checklist will also be part of the transparent peer-review file.

- FUNDING INFO: the following should be in the system as separate funders, via the More Funders option - the Featured Areas Research Center Program of the Ministry of Education, Taiwan, the Students in Direct Pursuit of a PhD Degree Scholarship, NSTC Graduate Research Fellowship-NSTC Selection. The text in the Comments box needs to be removed.

- Please include Acknowledgments in the ms file at the next submission.

- All EV figures need to be provided as separate production quality Figure files and their legends should be provided in the ms file, after the main figure legends. The correct nomenclature should be Figure EV1-Figure EV5 in all places. Your Appendix Fig 1 can be Fig EV6 instead. The Appendix table 1 can be Table EV1 but Appendix table 2 needs to be called Dataset EV1.

- "Suppl. Table 1" is not a correct callout, please correct.

- The Source Data needs to be re-organized into one zip folder per figure (upldd separately). Very large Source Data can be uploaded on the BioStudies website instead, but we need the direct and accessible URL provided in the Data Availability Section.

* Figure Legends - Comments *

- Please note that the exact p values are not provided in the legends of figures 2E', F', G'; 5F, G; 6F, K. Please provide exact values as reasonable.
- Please note that information related to n is missing in the legends of figures 1H, 2E-F; 3D, G; 4B, D
- Please note that the error bars are not defined in the legends of figures 1D, H; 2E-F; 3D, G; 4B, D; 5F, G, 6F, K.
- Please note that the dashed lines are not defined in the legend of figure 2A. This needs to be rectified.
- Please note that the yellow arrow heads are not defined in the legend of figure 5C. This needs to be rectified.

I would like to suggest some minor changes to the abstract that needs to be written in present tense. Do you agree with this:

Vocal communication is fundamental for social interaction across species, yet the neural mechanisms that shape vocal circuit development remain poorly understood despite their relevance to neurodevelopmental disorders. Here, we investigate vocal circuit development in mice using isolation-induced ultrasonic vocalizations (USV) in neonates. An activity-tagging approach identifies the ventromedial prefrontal cortex (vmPFC) as a cortical region strongly activated during USV emission. We find a predictable temporal correlation between vmPFC activity and USV emission using in vivo fiber photometry. Selective activation and inhibition of vmPFC neurons establishes a causal role of vmPFC in vocalization. Interestingly, chronic activation of vmPFC neurons not only increases Foxp2, a gene implicated in childhood speech apraxia, but also Vglut1-labeled synapses in the striatum, suggesting that activity-dependent increases in Foxp2 may promote corticostriatal synaptogenesis. Consistent with this finding, neonatal vmPFC activation partially rescues USV deficits in Foxp2 heterozygous mutant mice. Collectively, our results identify the vmPFC-striatal circuit as a key regulator of neonatal vocalization and suggest that Foxp2 may mediate activity-dependent development of vocal circuits.

EMBO press papers are accompanied online by A) a short (1-2 sentences) summary of the findings and their significance, B) 2-3 bullet points highlighting key results and C) a synopsis image that is exactly 550 pixels wide and 300 pixels high. The synopsis image should provide a sketch of the major findings, like a graphical abstract. Please note that text needs to be readable at the

final size. Please send us this information along with the final manuscript.

I look forward to seeing a final version of your manuscript as soon as possible.
Esther

Esther Schnapp, PhD
Senior Editor
EMBO reports

Referee #3:

The authors correctly addressed all my concerns. I suggest publication without further revision.

Referee #4:

In general, the authors have added new data and analyses that adequately address the concerns I raised.

All minor editorial requests have been addressed by the authors.

Dr. Hsiao-Ying Kuo
National Yang Ming Chiao Tung University
Institute of Anatomy and Cell Biology
Taiwan

Dear Dr. Kuo,

I am very pleased to accept your manuscript for publication in the next available issue of EMBO reports. Thank you for your contribution to our journal.

Your manuscript will be processed for publication by EMBO Press. It will be copy edited and you will receive page proofs prior to publication. Please note that you will be contacted by Springer Nature Author Services to complete licensing and payment information.

You may qualify for financial assistance for your publication charges - either via a Springer Nature fully open access agreement or an EMBO initiative. Check your eligibility: https://link.springer.com/journal/44319/how-to-publish-with-us

Should you be planning a Press Release on your article, please get in contact with embo_production@springernature.com as early as possible in order to coordinate publication and release dates.

If you have any questions, please do not hesitate to contact the Editorial Office. Thank you for your contribution to EMBO Reports.

Best regards,
Esther

Esther Schnapp, PhD
Senior Editor
EMBO reports

------------------------------------------------

>>> Please note that it is EMBO Reports policy for the transcript of the editorial process (containing referee reports and your response letter) to be published as an online supplement to each paper. If you do NOT want this, you will need to inform the Editorial Office via email immediately. More information is available here: https://link.springer.com/partners/embo-press/editorial-policies#Peer%20review

## EMBO Press Author Checklist

| | |
|---|---|
| Corresponding Author Name: Hsiao-Ying Kuo | |
| Journal Submitted to: EMBO Reports | |
| Manuscript Number: EMBOR-2025-62179V2 | |

**USEFUL LINKS FOR COMPLETING THIS FORM**
The EMBO Journal - Author Guidelines
EMBO Reports - Author Guidelines
Molecular Systems Biology - Author Guidelines
EMBO Molecular Medicine - Author Guidelines

**Reporting Checklist for Life Science Articles (updated January**
This checklist is adapted from Materials Design Analysis Reporting (MDAR) Checklist for Authors. MDAR establishes a minimum set of requirements in transparent reporting in the life sciences (see Statement of Task: 10.31222/osf.io/9sm4x). Please follow the journal's guidelines in preparing your
**Please note that a copy of this checklist will be published alongside your article.**

**Abridged guidelines for figures**
**1. Data**
The data shown in figures should satisfy the following conditions:
- the data were obtained and processed according to the field's best practice and are presented to reflect the results of the experiments in an accurate and unbiased manner.
- ideally, figure panels should include only measurements that are directly comparable to each other and obtained with the same assay.
- plots include clearly labeled error bars for independent experiments and sample sizes. Unless justified, error bars should not be shown for technical
- if n<5, the individual data points from each experiment should be plotted. Any statistical test employed should be justified.
- Source Data should be included to report the data underlying figures according to the guidelines set out in the authorship guidelines on Data

**2. Captions**
Each figure caption should contain the following information, for each panel where they are relevant:
- a specification of the experimental system investigated (eg cell line, species name).
- the assay(s) and method(s) used to carry out the reported observations and measurements.
- an explicit mention of the biological and chemical entity(ies) that are being measured.
- an explicit mention of the biological and chemical entity(ies) that are altered/varied/perturbed in a controlled manner.
- the exact sample size (n) for each experimental group/condition, given as a number, not a range;
- a description of the sample collection allowing the reader to understand whether the samples represent technical or biological replicates (including how many animals, litters, cultures, etc.).
- a statement of how many times the experiment shown was independently replicated in the laboratory.
- definitions of statistical methods and measures:
  - common tests, such as t-test (please specify whether paired vs. unpaired), simple χ2 tests, Wilcoxon and Mann-Whitney tests, can be unambiguously identified by name only, but more complex techniques should be described in the methods section;
  - are tests one-sided or two-sided?
  - are there adjustments for multiple comparisons?
  - exact statistical test results, e.g., P values = x but not P values < x;
  - definition of 'center values' as median or average;
  - definition of error bars as s.d. or s.e.m.

**Please complete ALL of the questions below.**
**Select "Not Applicable" only when the requested information is not relevant for your study.**

### Materials

| Newly Created Materials | Information included in the manuscript? | In which section is the information available? (Reagents and Tools Table, Materials and Methods, Figures, Data Availability Section) |
|---|---|---|
| New materials and reagents need to be available; do any restrictions apply? | Not Applicable | |

| Antibodies | Information included in the manuscript? | In which section is the information available? (Reagents and Tools Table, Materials and Methods, Figures, Data Availability Section) |
|---|---|---|
| For **antibodies** provide the following information:<br>- Commercial antibodies: RRID (if possible) or supplier name, catalogue number and or/clone number<br>- Non-commercial: RRID or citation | Yes | Materials and Methods |

| DNA and RNA sequences | Information included in the manuscript? | In which section is the information available? (Reagents and Tools Table, Materials and Methods, Figures, Data Availability Section) |
|---|---|---|
| **Short novel DNA or RNA including primers, probes**: provide the sequences. | Yes | Materials and Methods |

| Cell materials | Information included in the manuscript? | In which section is the information available? (Reagents and Tools Table, Materials and Methods, Figures, Data Availability Section) |
|---|---|---|
| **Cell lines:** Provide species information, strain. Provide accession number in repository **OR** supplier name, catalog number, clone number, and/**OR** RRID. | Not Applicable | |
| **Primary cultures:** Provide species, strain, sex of origin, genetic modification status. | Not Applicable | |
| Report if the cell lines were recently **authenticated** (e.g., by STR profiling) and tested for mycoplasma contamination. | Not Applicable | |

| Experimental animals | Information included in the manuscript? | In which section is the information available? (Reagents and Tools Table, Materials and Methods, Figures, Data Availability Section) |
|---|---|---|
| **Laboratory animals or Model organisms:** Provide species, strain, sex, age, genetic modification status. Provide accession number in repository **OR** supplier name, catalog number, clone number, **OR** RRID. | Yes | Materials and Methods |
| **Animal observed in or captured from the field:** Provide species, sex, and age where possible. | Not Applicable | |
| Please detail **housing and husbandry conditions**. | Yes | Materials and Methods |

| Plants and microbes | Information included in the manuscript? | In which section is the information available? (Reagents and Tools Table, Materials and Methods, Figures, Data Availability Section) |
|---|---|---|
| **Plants:** provide species and strain, ecotype and cultivar where relevant, unique accession number if available, and source (including location for collected wild specimens). | Not Applicable | |
| **Microbes:** provide species and strain, unique accession number if available, and source. | Not Applicable | |

| Human research participants | Information included in the manuscript? | In which section is the information available? (Reagents and Tools Table, Materials and Methods, Figures, Data Availability Section) |
|---|---|---|
| If collected and within the bounds of privacy constraints report on age, sex and gender or ethnicity for all study participants. | Not Applicable | |

| Core facilities | Information included in the manuscript? | In which section is the information available? (Reagents and Tools Table, Materials and Methods, Figures, Data Availability Section) |
|---|---|---|
| If your work benefited from core facilities, was their service mentioned in the acknowledgments section? | Not Applicable | |

### Design

| **Study protocol** | **Information included in the manuscript?** | **In which section is the information available?** (Reagents and Tools Table, Materials and Methods, Figures, Data Availability Section) |
|---|---|---|
| If study protocol has been **pre-registered, provide DOI in the manuscript**. For clinical trials, provide the trial registration number **OR** cite DOI. | Yes | Materials and Methods |
| Report the **clinical trial registration number** (at ClinicalTrials.gov or equivalent), where applicable. | Not Applicable | |

| **Laboratory protocol** | **Information included in the manuscript?** | **In which section is the information available?** (Reagents and Tools Table, Materials and Methods, Figures, Data Availability Section) |
|---|---|---|
| Provide DOI OR other citation details if **external detailed step-by-step protocols** are available. | Not Applicable | |

| **Experimental study design and statistics** | **Information included in the manuscript?** | **In which section is the information available?** (Reagents and Tools Table, Materials and Methods, Figures, Data Availability Section) |
|---|---|---|
| Include a statement about **sample size** estimate even if no statistical methods were used. | Not Applicable | |
| Were any steps taken to minimize the effects of subjective bias when allocating animals/samples to treatment (e.g. **randomization procedure**)? If yes, have they been described? | Yes | Materials and Methods |
| Include a statement about **blinding** even if no blinding was done. | Yes | Materials and Methods |
| Describe **inclusion/exclusion criteria** if samples or animals were excluded from the analysis. Were the criteria pre-established?<br><br>If sample or data points were omitted from analysis, report if this was due to attrition or intentional exclusion and provide justification. | Yes | Materials and Methods |
| For every figure, are **statistical tests** justified as appropriate? Do the data meet the assumptions of the tests (e.g., normal distribution)? Describe any methods used to assess it. Is there an estimate of variation within each group of data? Is the variance similar between the groups that are being statistically compared? | Yes | Materials and Methods; Figure Legend; Appendix Table |

| **Sample definition and in-laboratory replication** | **Information included in the manuscript?** | **In which section is the information available?** (Reagents and Tools Table, Materials and Methods, Figures, Data Availability Section) |
|---|---|---|
| In the figure legends: state number of times the experiment was **replicated** in laboratory. | Yes | Figure Legend |
| In the figure legends: define whether data describe **technical or biological replicates**. | Yes | Materials and Methods; Figure Legend |

## Ethics

| **Ethics** | **Information included in the manuscript?** | **In which section is the information available?** (Reagents and Tools Table, Materials and Methods, Figures, Data Availability Section) |
|---|---|---|
| Studies involving **human participants**: State details of **authority granting ethics approval** (IRB or equivalent committee(s), provide reference number for approval. | Not Applicable | |
| Studies involving **human participants**: Include a statement confirming that **informed consent** was obtained from all subjects and that the experiments conformed to the principles set out in the WMA Declaration of Helsinki and the Department of Health and Human Services Belmont Report. | Not Applicable | |
| Studies involving **human participants:** For publication of **patient photos**, include a statement confirming that consent to publish was obtained. | Not Applicable | |
| Studies involving experimental **animals**: State details of **authority granting ethics approval** (IRB or equivalent committee(s), provide reference number for approval. Include a statement of compliance with ethical regulations. | Yes | Materials and Methods |
| Studies involving **specimen and field samples:** State if relevant **permits** obtained, provide details of authority approving study; if none were required, explain why. | Not Applicable | |

| **Dual Use Research of Concern (DURC)** | **Information included in the manuscript?** | **In which section is the information available?** (Reagents and Tools Table, Materials and Methods, Figures, Data Availability Section) |
|---|---|---|
| Could your study fall under dual use research restrictions? Please check biosecurity documents and list of **select agents and toxins** (CDC): https://www.selectagents.gov/sat/list.htm | Not Applicable | |
| If you used a select agent, is the security level of the lab appropriate and reported in the manuscript? | Yes | Materials and Methods |
| If a study is subject to dual use research of concern regulations, is the name of the **authority granting approval and reference number** for the regulatory approval provided in the manuscript? | Not Applicable | |

## Reporting

The MDAR framework recommends adoption of discipline-specific guidelines, established and endorsed through community initiatives. Journals have their own policy about requiring specific guidelines and recommendations to complement MDAR.

| **Adherence to community standards** | **Information included in the manuscript?** | **In which section is the information available?** (Reagents and Tools Table, Materials and Methods, Figures, Data Availability Section) |
|---|---|---|
| State if relevant guidelines or checklists (e.g., **ICMJE, MIBBI, ARRIVE, PRISMA**) have been followed or provided. | Not Applicable | |
| For **tumor marker prognostic studies,** we recommend that you follow the **REMARK** reporting guidelines (see link list at top right). See author guidelines, under 'Reporting Guidelines'. Please confirm you have followed these guidelines. | Not Applicable | |
| For **phase II and III randomized controlled trials**, please refer to the **CONSORT** flow diagram (see link list at top right) and submit the CONSORT checklist (see link list at top right) with your submission. See author guidelines, under 'Reporting Guidelines'. Please confirm you have submitted this list. | Not Applicable | |

## Data Availability

| **Data availability** | **Information included in the manuscript?** | **In which section is the information available?** (Reagents and Tools Table, Materials and Methods, Figures, Data Availability Section) |
|---|---|---|
| Have **primary datasets** been deposited according to the journal's guidelines (see 'Data Deposition' section) and the respective accession numbers provided in the Data Availability Section? | Yes | Materials and Methods |
| Were **human clinical and genomic datasets** deposited in a public access-controlled repository in accordance to ethical obligations to the patients and to the applicable consent agreement? | Not Applicable | |
| Are **computational models** that are central and integral to a study available without restrictions in a machine-readable form? Were the relevant accession numbers or links provided? | Not Applicable | |
| If publicly available data were reused, provide the respective **data citations in the reference list**. | Not Applicable | |
